# Structural Landscape of α-Acetamidocinnamic Acid Cocrystals with Bipyridine-Based Coformers: Influence of Crystal Packing on Their Thermal and Photophysical Properties

**DOI:** 10.1021/acs.cgd.3c01374

**Published:** 2024-02-09

**Authors:** Daniel Ejarque, Teresa Calvet, Mercè Font-Bardia, Josefina Pons

**Affiliations:** † Departament de Química, Universitat Autònoma de Barcelona, 08193-Bellaterra, Barcelona, Spain; ‡ Departament de Mineralogia, Petrologia i Geologia Aplicada, Universitat de Barcelona, Martí i Franquès s/n, 08028 Barcelona, Spain; § Unitat de Difracció de Raig-X, Centres Científics i Tecnològics de la Universitat de Barcelona (CCiTUB), Universitat de Barcelona, Solé i Sabarís, 1-3, 08028 Barcelona, Spain

## Abstract

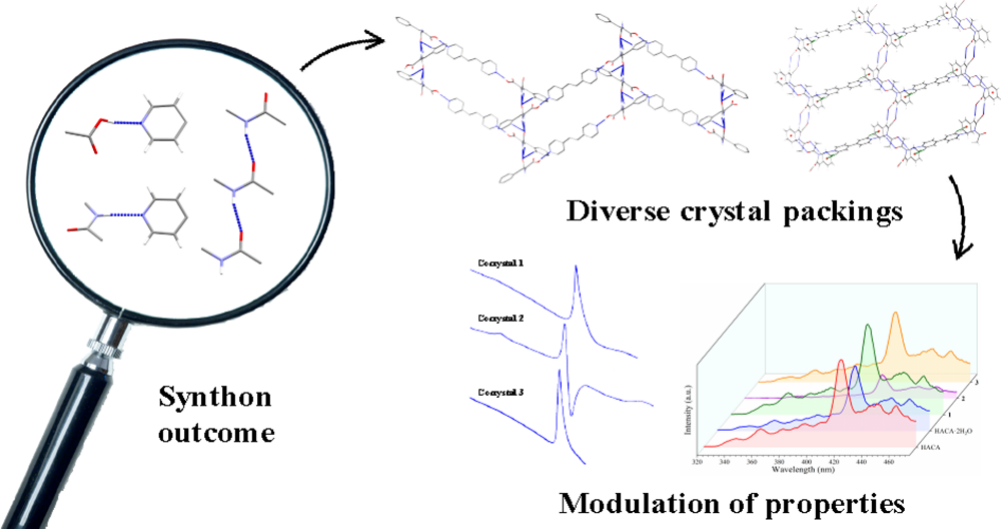

Controlling the supramolecular synthon outcome in systems with different functionalities has been a key factor for the design of supramolecular materials, which also affected their physicochemical properties. In this contribution, we have analyzed the structural landscape of α-acetamidocinnamic acid (HACA) aiming to find its synthon outcome from the competitivity between its acidic and amidic groups. We prepared four multicomponent forms including one dihydrate (HACA·2H_2_O) and three cocrystals bearing different bipyridine coformers with formulas (HACA)_2_(1,2-bpe) (**1**), (HACA)_2_(4,4′-azpy) (**2**), and (HACA)_2_(4,4′-bipy)_3_ (**3**) (1,2-bpe = 1,2-*bis*(4-pyridyl)ethylene; 4,4′-azpy = 4,4′-azopyridine; 4,4′-bipy = 4,4′-bipyridine). First, we applied a virtual screening approach to assess the feasibility of cocrystal formation. Then, we synthesized the cocrystals, *via* liquid-assisted grinding (LAG) (**1** and **2**) or solvothermal (**3**) techniques, and single crystals of HACA, and their four multicomponent forms were obtained showing different synthons and crystal packings. Besides, a Cambridge Structural Database (CSD) search of the cocrystals presenting bipyridine-type coformers and molecules with acid and amide functionalities was performed, and the observed synthon occurrences as well as the possibility of synthon modification by tuning the H-donor/H-acceptor propensity of the acidic and amidic groups were shown. Finally, we measured their thermal and photophysical properties, which were correlated with their structural features.

## Abstract 2

Different crystalline forms containing α-acetamidocinnamic acid (HACA), including one dihydrate and three bipyridine-based cocrystals, have been prepared to study the role of their acidic and amidic functionalities in different environments. Their thermal and photophysical properties have been correlated with their structural features.

## Introduction

The cocrystallization of relevant molecules with carefully selected complementary coformers has become an interesting route for the development of new materials with improved applications in the fields of pharmaceuticals,^1,2^ energetics,^3,4^ and photophysics,^5,6^ among others.^7,8^ Researchers initially focused on the discovery of reliable associations between functional groups, denoted as supramolecular synthons,^9^ from which the field has been growing, allowing the recent formation of more sophisticated cocrystals including ternary,^10,11^ quaternary,^12,13^ or even cocrystals with higher complexity^14,15^ prepared using different strategies including, *inter alia,* graded synthon hierarchies,^16,17^ shape and size mimicry,^18,19^ or cooperativity and anticooperativity approaches.^20,21^

In this context, the synthon competitivity between functional groups within a same molecule has been a major concern,^22−24^ and even though a myriad of cocrystals with multifunctional molecules have been reported,^25−29^ the control of their synthons remains poorly understood, and additional investigations are necessary aiming to find hierarchical combinations of supramolecular synthons referred to as supramolecular orthogonality^30−33^ toward the design of advanced functional supramolecular materials.^34−37^ Within this frame, our group has previously studied some cocrystals based on the acid···amide,^38^ and acid···pyridine^39,40^ heterosynthons. In addition, we recently applied virtual screening techniques to assess their feasible formation.^40^ Following these works, in this contribution, we will focus on the study of the structural landscape of α-acetamidocinnamic acid (HACA), which contains an amide and a carboxylic acid moiety as functional groups. To date, only one dihydrate and one cocrystal have been reported.^40−42^ The dihydrate structure connected the HACA molecules through masked synthons^43^ of water clusters supported by acid···amide interactions,^41,42^ whereas the cocrystal contained 2-pyridone (Pdon), presenting Pdon···acid and amide···amide synthons.^40^ However, previous studies performed in the group with coordination compounds suggested a tendency of the HACA molecules toward being ordered by amide···amide patterns, leading to supramolecular chains or cycles depending on the synthetic conditions.^44−46^

Therefore, in this work, we will evaluate the behavior of HACA when combined with bipyridine-type coformers benefiting from the well-known acid···pyridine heterosynthon.^23,47^ We have assessed the feasibility of cocrystal formation of the selected combinations using a virtual screening methodology based on the molecular electrostatic potential (MEP) surfaces.^48,49^ Next, three cocrystals have been successfully obtained in powder and crystalline forms, which enabled the study of their crystal structures yielding (HACA)_2_(1,2-bpe) (**1**), (HACA)_2_(4,4′-azpy) (**2**), and (HACA)_2_(4,4′-bipy)_3_ (**3**) (1,2-bpe = 1,2-*bis*(4-pyridyl)ethylene; 4,4′-azpy = 4,4′-azopyridine; 4,4′-bipy = 4,4′-bipyridine), showing an unusual behavior of the HACA synthons in **3**, where the acid···pyridine is combined with an uncommon amide···pyridine heterosynthon. In addition, we have been able to obtain crystals of the HACA form, which was unreported, and its dihydrate (HACA·2H_2_O), which was revisited,^41,42^ for comparing the HACA behavior in these scenarios. Finally, the thermal and photophysical properties of HACA, HACA·2H_2_O, and cocrystals **1**–**3** have been measured and their structure–property relationships investigated.

## Experimental Section

### Materials and General Details

α-Acetamidocinnamic acid (HACA), 1,2-*bis*(4-pyridyl)ethylene (1,2-bpe), 4,4′-azopyridine (4,4′-azpy), 4,4′-bipyridine (4,4′-bipy), and methanol (MeOH), dichloromethane (CH_2_Cl_2_), and diethyl ether (Et_2_O) as solvents were purchased from Sigma-Aldrich. The water used for all the experiments was Milli-Q. Deuterated methanol (CD_3_OD) was used for the NMR experiments and was purchased from Eurisotop. All of them were used without further purification. All the reactions and manipulations involving temperatures higher than room temperature (RT) were done in a Digiheat-TFT furnace using sealed vials under autogenous pressure. Powder X-ray diffraction (PXRD) patterns were measured with a Panalytical X’pert PRO MPD apparatus using a monochromatic Cu Kα radiation with a λ = 1.5406 Å. All of them were recorded from 2θ = 5 to 30° with a step scan of 0.01671°. Melting points (Mp's) were measured on a Stuart Melting Point Apparatus SMP30 using a 2.0 °C/min step rate from RT to 200 °C. Elemental analyses (C, H, N) were carried out on a Thermo Scientific Flash 2000 CHNS Analyzer. FTIR-ATR spectra were recorded on a PerkinElmer spectrometer equipped with an attenuated total reflectance (ATR) accessory (model MKII Golden Gate) with diamond window in the range 4000–500 cm^–1^. ^1^H, ^13^C{^1^H}, and DEPT-135 NMR spectra were recorded on a Bruker Ascend 300 MHz spectrometer in CD_3_OD solutions at RT. All chemical shifts (δ) are given in ppm relative to tetramethylsilane (Me_4_Si) as internal standard. Simultaneous thermogravimetric/differential thermal analysis (TG/DTA) determinations were carried out using 78.8 mg (HACA·2H_2_O), 49.1 mg (**1**), 64.0 mg (**2**), and 56.2 mg (**3**) in a Netzsch STA 409 instrument with an aluminum oxide powder (Al_2_O_3_) crucible and heating at 5 °C·min^–1^ from RT to 330 °C under a nitrogen atmosphere with a flow rate of 80 mL·min^–1^. Al_2_O_3_ (PerkinElmer 0419-0197) was used as standard. Solid-state UV–vis spectra were acquired using a Cary 4000 spectrophotometer between 200 and 800 nm. Solid-state photoluminescence measurements were recorded using a Varian Cary Eclipse Fluorescence spectrophotometer. The CIE 1931 chromaticity diagram was generated using the Origin Pro 2019b software.

### Obtention of Single Crystals of HACA and HACA·2H_2_O

During the experimental trials for obtaining single crystals suitable for X-ray diffraction of a cocrystal composed by HACA and 4,4′-bipy, it was found that, in some experiments, crystalline samples corresponding to HACA·2H_2_O and HACA were isolated. Single crystals of the HACA form were successfully obtained when 20.0 mg of HACA was dissolved in a mixture of 5.5 mL of nitromethane and 8.0 μL of pyridine, and the resulting solution was left to slowly evaporate at RT for 1 month. For the obtention of single crystals of HACA·2H_2_O, 200 mg of HACA was dissolved in 6.0 mL of Milli-Q water and 0.8 mL of EtOH, and the solution was kept at 80 °C during 12 h at autogenous pressure. Then, it was allowed to cool down to RT, obtaining single crystals. Phase purity of both samples was verified by PXRD (SI Figures S1 and S2).

HACA. Mp 189–190 °C. Elemental analysis calcd (%) for C_11_H_11_NO_3_ (205.21): C 64.38; H 5.40; N 6.83; found: C 64.25; H 5.37; N 6.74. FTIR-ATR (wavenumber, cm^–1^): 3247(m) [ν_st_(N–H)], 3132–2721 (br) [ν_st_(O–H)_HACA_ + ν_st_(C–H)_ar_ + ν_st_(C–H)_alk_ + ν_st_(C–H)_al_], 2699–2310 [ν_st_(N/O–H···N/O)], 1683(m) [ν_st_(C=O)_COOH_], 1650(s) [ν_st_(C=O)_CONH_], 1640(s) [ν_st_(C=C/C=N)], 1510(m), 1490(w), 1451(w), 1428(w), 1373(w), 1330(w), 1318(w), 1295(m), 1264(s) [δ(C=C/C=N)], 1207(m), 1188(w), 1131(m), 1079(w), 1040(w), 1010(w), 999(w), 981(w), 930(m) [δ_ip_(C–H)], 907(w) [δ_ip_(C–H)], 843(w), 772(m) [δ_oop_(C–H)], 747(w), 726(w), 690(s) [δ_oop_(C–H)], 653(m), 614(s), 548(m), 517(s). UV–vis: λ_max-Abs_ = 246, 294, 390 nm. Fluorescence: λ_exc_ = 250 nm → λ_em_ = 420 nm.

HACA·2H_2_O. Isolated yield: 186 mg (79.1%) (based on HACA). Mp 189–190 °C. Elemental analysis calcd (%) for C_11_H_15_NO_5_ (241.24): C 54.77; H 6.27; N 5.81; found: C 54.65; H 6.08; N 5.69. FTIR-ATR (wavenumber, cm^–1^): 3623–2848(br) [ν_st_(O–H)_water_ + ν_st_(N–H) + ν_st_(C–H)_ar_ + ν_st_(C–H)_alk_ + ν_st_(C–H)_al_], 3515(m) [ν_st_(O–H)_water_], 3307(s) [ν_st_(N–H)], 2682–2130(br) [ν_st_(O–H)_HACA_], 2045–1738(br) [ν_st_(N/O–H···N/O)], 1685(w) [ν_st_(C=O)_COOH_], 1650(s) [ν_st_(C=O)_CONH_], 1629(s) [ν_st_(C=C/C=N)], 1508(m), 1492(m), 1449(w), 1373(w), 1322(w), 1291(m), 1268(m) [δ(C=C/C=N)], 1203(m), 1184(w), 1164(w), 1125(w), 999(w) [δ_ip_(C–H)], 979(w) [δ_ip_(C–H)], 956(w), 876(w), 841(w), 774(m) [δ_oop_(C–H)], 733(w), 688(m) [δ_oop_(C–H)], 663(w), 647(w), 599(w), 577(w), 540(m), 523(w). UV–vis: λ_max-Abs_ = 252, 324, 400 nm. Fluorescence: λ_exc_ = 250 nm → λ_em_ = 420 nm.

### Synthesis of (HACA)_2_(1,2-bpe) (**1**) and (HACA)_2_(4,4′-azpy) (**2**)

Both cocrystals were prepared placing 50.0 mg of HACA (0.244 mmol) and 0.122 mmol of 1,2-bpe (22.3 mg, **1**) or 4,4′-azpy (22.5 mg, **2**) in an agate mortar and then grinding the mixture in the presence of 100 μL of MeOH until a homogeneous powder was obtained. Phase purity of both samples was verified by PXRD (SI Figures S3 and S4).

For the obtention of single crystals suitable for X-ray diffraction, 77.0 mg of HACA (0.375 mmol) and 0.188 mmol of 1,2-bpe (34.8 mg, **1**) or 4,4′-azpy (34.6 mg, **2**) were weighted on a glass vial. Then, a mixture of 3.0 mL of Milli-Q water and 51.4 μL of EtOH (**1**) or 4.5 mL of Milli-Q water and 77.8 μL of EtOH (**2**) was added. The vials were placed in an oven at 90 °C during 12 h, obtaining yellow (**1**) and reddish (**2**) solutions, which were allowed to cool down to RT, obtaining single crystals suitable for X-ray diffraction.

**1**. Isolated yield: 68.5 mg (94.9%) (based on HACA) Mp 194–195 °C. Elemental analysis calcd (%) for C_34_H_32_N_4_O_6_ (592.64): C 68.91; H 5.44; N 9.45; found: C 68.70; H 5.27; N 9.32. FTIR-ATR (wavenumber, cm^–1^): 3227(m) [ν_st_(N–H)], 3173–3024(w) [ν_st_(C–H)_ar_ + ν_st_(C–H)_alk_], 2797(w) [ν_st_(C–H)_al_], 2669–2145(br) [ν_st_(O–H)_HACA_], 2132–1758(br) [ν_st_(N/O–H···N/O)], 1687(sh) [ν_st_(C=O)_COOH_], 1654(sh) [ν_st_(C=O)_CONH_], 1630(s), 1601(s) [ν_st_(C=C/C=N)], 1521(m), 1491(m), 1445(w), 1421(m) [δ(C=C/C=N)], 1369(m), 1329(w), 1315(w), 1288(m), 1236(m), 1192(s), 1180(s), 1128(m), 1067(m), 1011(s) [δ_ip_(C–H)], 976(s) [δ_ip_(C–H)], 957(m), 939(m), 864(w), 839(w), 824(s) [δ_oop_(C–H)], 775(s) [δ_oop_(C–H)], 741(w), 710(m), 690(s) [δ_oop_(C–H)], 608(w), 584(w), 550(s), 519(m). ^1^H NMR (300 MHz; CD_3_OD; Me_4_Si, 298 K): δ = 8.56 [4H, br, *o*-*H*_1,2-bpe_], 7.67 [4H, ^3^*J* = 5.7 Hz, *m*-*H*_1,2-bpe_], 7.57 [4H, dd, ^3^*J* = 7.8 Hz, ^4^*J* = 1.5 Hz, *o*-*H*_HACA_], 7.51 [2H, s, C*H*=C*H*_1,2-bpe_], 7.48 [2H, s, NH–C–C*H*_HACA_], 7.38 [6H, m, *m*-*H*_HACA_ + *p*-*H*_HACA_], 2.10 [6H, s, C*H*_3,HACA_]. ^13^C{^1^H} NMR (75 MHz; CD_3_OD; Me_4_Si, 298 K): δ = 173.2 [NH-*C*O_HACA_], 168.3 [*C*OOH_HACA_], 150.4 [*o*-*C*_1,2-bpe_], 146.4 [N–CH-CH-*C*_1,2-bpe_], 135.4 [HN-C-*C*H_HACA_], 135.1 [HN-C–CH-*C*_HACA_], 132.2 [Py-*C*H_1,2-bpe_], 130.9 [*o*-*C*_HACA_], 130.6 [*p*-*C*_HACA_], 129.7 [*m*-*C*_HACA_], 127.2 [HOOC-*C*_HACA_], 123.2 [*m*-*C*_1,2-bpe_], 22.5 [CO-*C*H_3,HACA_]. DEPT-135 NMR (75 MHz; CD_3_OD; Me_4_Si, 298 K): δ = 150.4 [*o*-*C*_1,2-bpe_], 135.4 [HN-C-*C*H_HACA_], 132.2 [Py-*C*H_1,2-bpe_], 130.8 [*o*-*C*_HACA_], 130.5 [*p*-*C*_HACA_], 129.6 [*m*-*C*_HACA_], 123.1 [*m*-*C*_1,2-bpe_], 22.5 [CO-*C*H_3,HACA_]. UV–vis: λ_max-Abs_ = 252, 322 nm. Fluorescence: λ_exc_ = 250 nm → λ_em_ = 420 nm.

**2**. Isolated yield: 69.3 mg (95.7%) (based on HACA) Mp 180–181 °C. Elemental analysis calcd (%) for C_32_H_30_N_6_O_6_ (594.62): C 67.83; H 5.34; N 9.89; found: C 67.58; H 5.13; N 9.75. FTIR-ATR (wavenumber, cm^–1^): 3281(m) [ν_st_(N–H)], 3109–3030(br) [ν_st_(C–H)_ar_ + ν_st_(C–H)_alk_], 2771(w) [ν_st_(C–H)_al_], 2680–2098(br) [ν_st_(O–H)_HACA_], 2025–1740(br) [ν_st_(N/O–H···N/O)], 1675(sh) [ν_st_(C=O)_COOH_], 1657(s) [ν_st_(C=O)_CONH_], 1634(s), 1595(m) [ν_st_(C=C/C=N)], 1512(s), 1489(s), 1443(w), 1412(m) [δ(C=C/C=N)], 1362(w), 1337(w), 1321(w), 1281(s), 1261(s), 1242(s), 1223(m), 1200(s), 1188(s), 1128(w), 1084(w), 1051(w), 1034(w), 1013(w) [δ_ip_(C–H)], 1001(w), 986(w) [δ_ip_(C–H)], 941(w), 915(w), 864(w), 839(s) [δ_oop_(C–H)], 775(s) [δ_oop_(C–H)], 754(w), 739(w), 720(w), 689(s) [δ_oop_(C–H)], 608(w), 582(w), 569(m), 542(s), 519(m). ^1^H NMR (300 MHz; CD_3_OD; Me_4_Si, 298 K): δ = 8.85 [4H, br, *o*-*H*_4,4′-azpy_], 7.91 [4H, d, ^3^*J* = 5.8 Hz, *m*-*H*_4,4′-azpy_], 7.57 [4H, ^3^*J* = 7.8 Hz, ^4^*J* = 1.5 Hz, *o*-*H*_HACA_], 7.48 [2H, s, NH–C–C*H*_HACA_], 7.38 [6H, m, *m*-*H*_HACA_ + *p*-*H*_HACA_], 2.10 [6H, s, C*H*_3,HACA_]. ^13^C{^1^H} NMR (75 MHz; CD_3_OD; Me_4_Si, 298 K): δ = 173.2 [NH-*C*O_HACA_], 168.2 [*C*OOH_HACA_], 158.5 [N–CH-CH-*C*_4,4′-azpy_], 152.3 [*o*-*C*_4,4′-azpy_], 135.5 [HN-C-*C*H_HACA_], 135.0 [HN-C–CH-*C*_HACA_], 130.9 [*o*-*C*_HACA_], 130.6 [*p*-*C*_HACA_], 129.7 [*m*-*C*_HACA_], 127.2 [HOOC-*C*_HACA_], 118.1 [*m*-*C*_4,4′-azpy_], 22.5 [CO-*C*H_3,ACA_]. DEPT-135 NMR (75 MHz; CD_3_OD; Me_4_Si, 298 K): δ = 152.2 [*o*-*C*_4,4′-azpy_], 135.5 [HN-C-*C*H_HACA_], 130.8 [*o*-*C*_HACA_], 130.5 [*p*-*C*_HACA_], 129.6 [*m*-*C*_HACA_], 118.1 [*m*-*C*_4,4′-azpy_], 22.5 [CO-*C*H_3,ACA_]. UV–vis: λ_max-Abs_ = 259, 314, 390, 492 nm. Fluorescence: λ_exc_ = 250 nm → λ_em_ = 420 nm.

### Synthesis of (HACA)_2_(4,4′-bipy)_3_ (**3**)

In a glass vial, 100 mg of HACA (0.487 mmol) and 119 mg of 4,4′-bipy (0.762 mmol) were dissolved in 2 mL of CH_2_Cl_2_ and 100 μL of MeOH, and then, the reaction mixture was kept at 50 °C for 5 h. Afterward, the solution was left evaporating at RT until the obtention of a solid, which was washed twice with 2 mL of Et_2_O. Phase purity of the sample was verified by PXRD (SI Figure S5). Single crystals suitable for X-ray diffraction were harvested from the solution of the reaction after slow evaporation at RT for 3 days.

**3**. Isolated yield: 202 mg (94.3%) (based on HACA) Mp 169–170 °C. Elemental analysis calcd (%) for C_52_H_46_N_8_O_6_ (878.97): C 71.06; H 5.27; N 12.75; found: C 70.89; H 5.16; N 12.57. FTIR-ATR (wavenumber, cm^–1^): 3217(w) [ν_st_(N–H)], 3140–3036(w) [ν_st_(C–H)_ar_ + ν_st_(C–H)_alk_], 2980–2789(w) [ν_st_(C–H)_al_], 2685–2141(br) [ν_st_(O–H)_HACA_], 2135–1769(br) [ν_st_(N/O–H···N/O)], 1711(sh) [ν_st_(C=O)_COOH_], 1682(s) [ν_st_(C=O)_CONH_], 1641(m), 1593(m) [ν_st_(C=C/C=N)], 1527(m), 1489(w), 1441(w), 1406(m) [δ(C=C/C=N)], 1367(m), 1329(w), 1284(m), 1256(m), 1223(w), 1203(m), 1140(m), 1097(w), 1067(m), 1041(w), 1013(w) [δ_ip_(C–H)], 991(w) [δ_ip_(C–H)], 962(w), 937(w), 908(w), 874(w), 862(w), 849(w), 804(s) [δ_oop_(C–H)], 758(m) [δ_oop_(C–H)], 735(w), 714(w), 698(s) [δ_oop_(C–H)], 654(w), 615(s), 590(w), 569(w), 555(w), 523(w). ^1^H NMR (300 MHz; CD_3_OD; Me_4_Si, 298 K): δ = 8.69 [12H, d, ^3^*J* = 5.4 Hz, *o*-*H*_4,4′-bipy_], 7.83 [12H, dd, ^3^*J* = 4.6 Hz, ^4^*J* = 1.6 Hz, *m*-*H*_4,4′-bipy_], 7.57 [4H, d, ^3^*J* = 6.9 Hz, *o*-*H*_HACA_], 7.48 [2H, s, NH–C–C*H*_HACA_], 7.37 [6H, m, *m*-*H*_HACA_ + *p*-*H*_HACA_], 2.10 [6H, s, C*H*_3,HACA_]. ^13^C{^1^H} NMR (75 MHz; CD_3_OD; Me_4_Si, 298 K): δ = 173.2 [NH-*C*O_HACA_], 168.3 [*C*OOH_HACA_], 151.1 [*o*-*C*_4,4′-bipy_], 147.4 [N–CH-CH-*C*_4,4′-bipy_], 135.4 [HN-C-*C*H_HACA_], 135.0 [HN-C–CH-*C*_HACA_], 130.9 [*o*-*C*_HACA_], 130.6 [*p*-*C*_HACA_], 129.7 [*m*-*C*_HACA_], 127.1 [HOOC-*C*_HACA_], 123.2 [*m*-*C*_4,4′-bipy_], 22.5 [CO-*C*H_3,ACA_]. DEPT-135 NMR (75 MHz; CD_3_OD; Me_4_Si, 298 K): δ = 151.1 [*o*-*C*_4,4′-bipy_], 135.4 [HN-C-*C*H_HACA_], 130.9 [*o*-*C*_HACA_], 130.6 [*p*-*C*_HACA_], 129.7 [*m*-*C*_HACA_], 123.2 [*m*-*C*_4,4′-bipy_], 22.5 [CO-*C*H_3,ACA_]. UV–vis: λ_max-Abs_ = 286 nm. Fluorescence: λ_exc_ = 250 nm → λ_em_ = 420 nm.

### X-ray Crystallographic Data

Colorless (HACA, **1**, **3**), yellow (HACA·2H_2_O), and orange (**2**) prism-like specimens were used for the X-ray crystallographic analysis. The X-ray intensity data were measured on a D8 Venture system equipped with a multilayer monochromator (λ = 0.71073 Å). For all the compounds, the frames were integrated using the Bruker SAINT Software Package (version-2018/3). The integration of the data with 0.70 Å (HACA and HACA·2H_2_O), 0.73 Å (**1**), 0.79 Å (**2**), and 0.75 Å (**3**) resolution, of which 3106 (HACA), 3675 (HACA·2H_2_O), 4070 (**1**), 3036 (**2**), and 5542 (**3**) reflections were independent, gave an average redundancy of 8.320 (HACA), 14.586 (HACA·2H_2_O), 1.000 (**1** and **3**), and 10.119 (**2**); completeness of 100.0% (HACA), 99.7% (HACA·2H_2_O), 99.9% (**1**), 98.2% (**2**), and 99.8% (**3**); and *R*_sig_ of 2.14% (HACA), 4.96% (HACA·2H_2_O), 2.76% (**1**), 2.58% (**2**), and 0.31% (**3**), presenting 2699 (86.90%) (HACA), 2709 (73.71%) (HACA·2H_2_O), 3607 (88.62%) (**1**), 2617 (86.20%) (**2**), and 5402 (97.47%) (**3**) reflections greater than 2σ(|*F*|^2^).

For all of them, the final cell constants and volume are based upon refinement of the XYZ-centroids of reflections above 20 σ(*I*). Data were corrected for absorption effects using the multi-scan method (SADABS). Crystal data and additional details of structure refinement for HACA, HACA·2H_2_O, and **1**–**3** are reported in Tables 1 and 2. Complete information about the crystal structure and molecular geometry is available in CIF format *via* CCDC 2308115 (HACA), 2308119 (HACA·2H_2_O), 2308117 (**1**), 2308118 (**2**), and 2308116 (**3**). Molecular graphics were generated using the Mercury 4.3.1 software^50^ using the POV-Ray image package.^51^ The color codes for all the molecular graphics are as follows: red (O), light blue (N), gray (C), and white (H). The topological analysis was done using the ToposPro 5.3.3.4 program,^52^ considering as H-bonds those interactions fulfilling the following premises: (i) d(H···A) ≤ 2.7 Å; (ii) d(D···A) ≤ 3.5 Å, and (iii) <D-H···A ≥ 120°.

**Table 1:** Crystal Data and Structure Refinement for HACA and HACA·2H_2_O

	HACA	HACA·2H_2_O
empirical formula	C_11_H_11_NO_3_	C_11_H_15_NO_5_
formula weight	205.21	241.24
*T* (K)	100(2)	100(2)
wavelength (Å)	0.71073	0.71073
system, space group	triclinic, P1̅	monoclinic, *P*2_1_/*c*
unit cell dimensions		
*a* (Å)	4.7420(2)	11.2636(12)
*b* (Å)	9.2342(5)	6.0648(5)
*c* (Å)	12.3019(6)	17.9814(19)
α (°)	71.777(2)	90
β (°)	82.641(2)	105.344(4)
γ (°)	87.300(2)	90
*V* (Å^3^)	507.44(4)	1184.6(2)
*Z*	2	4
*D*_calc_ (mg/m^3^)	1.343	1.353
μ (mm^–1^)	0.099	0.107
*F* (000)	216	512
crystal size (mm^–3^)	0.298 × 0.173 × 0.037	0.284 × 0.111 × 0.086
*hkl* ranges	–6 ≤ *h* ≤ 6	–16 ≤ *h* ≤ 16
	–13 ≤ *k* ≤ 13	–8 ≤ *k* ≤ 8
	–17 ≤ *l* ≤ 17	–25 ≤ *l* ≤ 25
θ range (°)	1.755 to 30.539	1.875 to 30.700
reflections collected/unique/[*R*_int_]	25843/3106/0.0378	53603/3675/0.1184
completeness to θ (%)	100.0	100.0
absorption correction	semiempirical from equivalents	semiempirical from equivalents
max and min transmission	0.7461 and 0.7199	0.7461 and 0.6472
refinement method	full-matrix least-squares on |*F*|^2^	full-matrix least-squares on |*F*|^2^
data/restrains/parameters	3106/0/137	3675/6/168
goodness-on-fit on *F*^2^	1.067	1.089
final *R* indices [*I* > 2σ(*I*)]	*R*_1_ = 0.0392, wR_2_ = 0.1035	*R*_1_ = 0.0700, wR_2_ = 0.1449
*R* indices (all data)	*R*_1_ = 0.0475, wR_2_ = 0.1112	*R*_1_ = 0.1023, wR_2_ = 0.1608
extinction coefficient	n/a	n/a
largest diff-peak and hole (e. Å^–3^)	0.414 and −0.282	0.366 and −0.378

**Table 2:** Crystal Data and Structure Refinement for **1**–**3**

	**1**	**2**	**3**
empirical formula	C_17_H_16_N_2_O_3_	C_16_H_15_N_3_O_3_	C_26_H_24_N_4_O_3_
formula weight	296.32	297.31	440.49
*T* (K)	100(2)	100(2)	100(2)
wavelength (Å)	0.71073	0.71073	0.71073
system, space group	monoclinic, *P*2_1_/*c*	monoclinic, *P*2_1_/*n*	triclinic, *P*1̅
unit cell dimensions			
*a* (Å)	8.7186(5)	8.6515(3)	10.0255(6)
*b* (Å)	9.4086(5)	9.5819(3)	10.2545(6)
*c* (Å)	19.9361(10)	17.7546(6)	11.2438(5)
α (°)	90	90	83.132(2)
β (°)	112.863(3)	100.252(2)	88.017(2)
γ (°)	90	90	75.299(2)
*V* (Å^3^)	1506.88(14)	1448.32(8)	1110.06(11)
*Z*	4	4	2
*D*_calc_ (mg/m^3^)	1.306	1.363	1.318
μ (mm^–1^)	0.091	0.097	0.088
*F* (000)	624	624	464
crystal size (mm^–3^)	0.187 × 0.058 × 0.038	0.239 × 0.104 × 0.101	0.185 × 0.046 × 0.034
*hkl* ranges	–11 ≤ *h* ≤ 11	–10 ≤ *h* ≤ 10	–13 ≤ *h* ≤ 13
	–12 ≤ *k* ≤ 12	–12 ≤ *k* ≤ 11	–13 ≤ *k* ≤ 13
	–27 ≤ *l* ≤ 27	–22 ≤ *l* ≤ 22	–14 ≤ *l* ≤ 14
θ range (°)	2.217 to 29.187	2.331 to 26.772	1.824 to 28.351
reflections collected/unique/[*R*_int_]	4070/4070/0.0530	30720/3036/0.0529	5542/5542/0.0750
completeness to θ (%)	100.0	100.0	100.0
absorption correction	semiempirical from equivalents	semiempirical from equivalents	semiempirical from equivalents
max and min transmission	0.7458 and 0.6443	0.7454 and 0.6234	0.7457 and 0.6271
refinement method	full-matrix least-squares on |*F*|^2^	full-matrix least-squares on |*F*|^2^	full-matrix least-squares on |*F*|^2^
data/restrains/parameters	4070/3/202	3036/0/207	5542/2/299
goodness-on-fit on *F*^2^	1.186	1.039	1.317
final *R* indices [*I* > 2σ(*I*)]	*R*_1_ = 0.0866, wR_2_ = 0.1748	*R*_1_ = 0.0427, wR_2_ = 0.1052	*R*_1_ = 0.0537, wR_2_ = 0.1003
*R* indices (all data)	*R*_1_ = 0.0958, wR_2_ = 0.1799	*R*_1_ = 0.0507, wR_2_ = 0.1109	*R*_1_ = 0.0551, wR_2_ = 0.1010
extinction coefficient	n/a	n/a	n/a
largest diff-peak and hole (e. Å^–3^)	0.618 and −0.486	0.834 and −0.213	0.248 and −0.363

### Computational Details

To evaluate the feasibility of the cocrystal formation considering our selected components, the virtual screening methodology developed by Hunter et al.^48,49^ was used to obtain the energetic difference (Δ*E*) in interaction site pairing energies between the pure components and the virtual cocrystals with the selected stoichiometries. This methodology was successfully applied in our group in a previous work.^40^ All the structures were energy-minimized using the COMPASS II force field in Materials Studio.^53,54^ Then, geometry optimizations were done using the density functional theory (DFT) with B3LYP/6-31G(+) theory level with the Gaussian09 software version D.01.^55^ The most stable conformations in the gas phase of each component were utilized for the MEP prediction because it has been shown that different conformations do not change significantly the H-donor (α) and H-acceptor (β) propensity values unless intramolecular interactions are possible to occur.^56^ The local minima and maxima from the MEP surfaces were extracted using the Multiwfn software,^57^ whereas their visualization and rendering were done in the VMD program.^58^

Hirshfeld surface analysis and energy frameworks of **1** and **2** were performed with CrystalExplorer 21.5.^59^ The Hirshfeld surfaces of each component of the cocrystals were calculated independently using an isovalue of 0.5 e × au^–3^. Moreover, both cocrystals were analyzed with energy frameworks with TONTO^60^ using a scale factor of 120 and the CE-B3LYP/6-31G(d,p) energy model^61−63^ starting from the corresponding .cif files. Each of the molecules of the cocrystals was confined in a cluster of 20 Å in the unit cell, including those crystallographically independent. The contribution of all the molecular pairs around the selected cluster was considered following a previously reported methodology.^61^ In addition, the calculation of the total energy for each interaction as well as the lattice energy (*E*_latt_) was done following the procedure reported elsewhere.^61−63^

## Results and Discussion

### Synthesis and Characterization

**Scheme 1:**
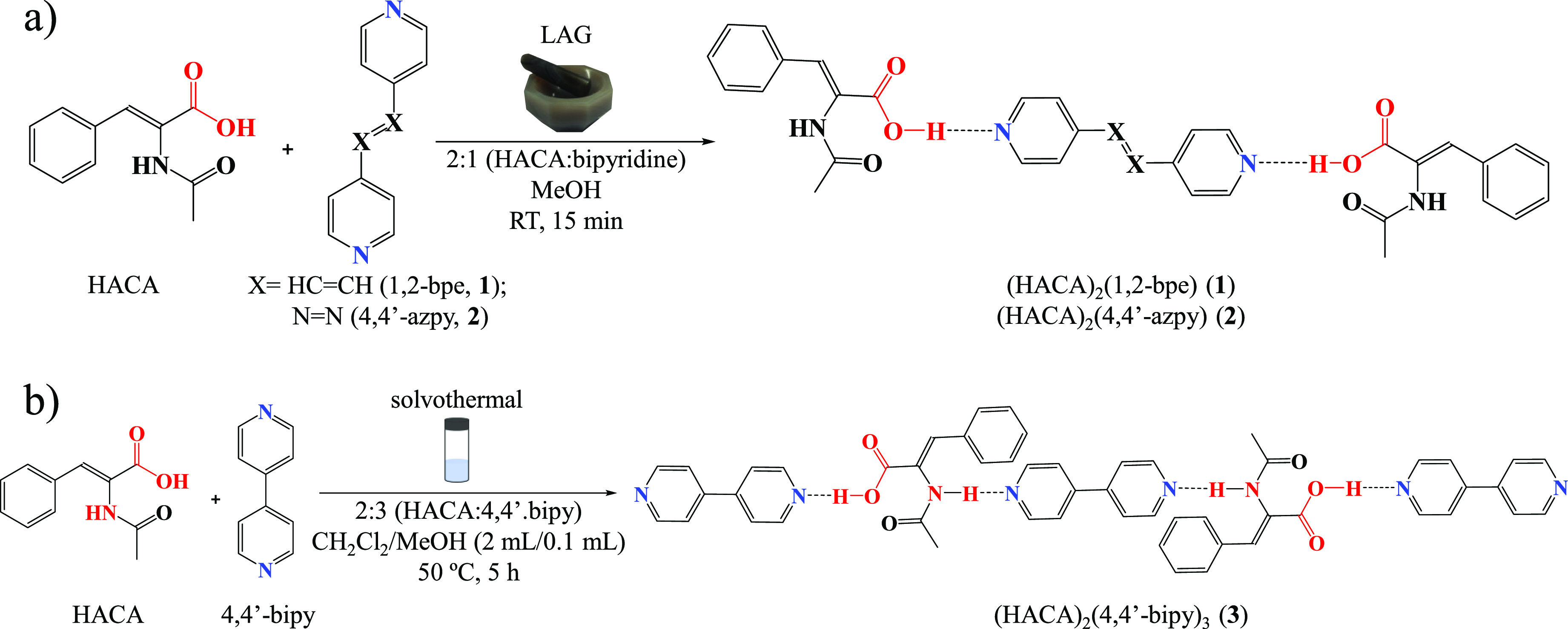
Outline of the Preparation of Cocrystals (a) **1** and **2**, and (b) **3**

Cocrystals **1** and **2** were obtained by liquid-assisted grinding (LAG) of HACA with their corresponding bipyridine coformers (1,2-bpe, **1**; 4,4′-azpy, **2**) using a small quantity of MeOH (Scheme 1a). Otherwise, to isolate cocrystal **3**, it was necessary to carry out the reaction in solvothermal conditions using CH_2_Cl_2_ and a small portion of MeOH as solvents at 50 °C (Scheme 1b). In addition, during the scanning performed for the obtention of single crystals of **3**, it was found that, in some conditions where H_2_O and MeOH were used as solvents, the crystals of the dihydrate form (HACA·2H_2_O) were obtained, whereas when nitromethane was employed, crystalline powders of HACA were isolated. The crystal structure of HACA·2H_2_O was previously elucidated at 295 K.^41,42^ However, we revisited it at 100 K, providing complete crystallographic information that was missing in the previous works. In addition, the crystal structure of HACA form was unreported, and we focus on the preparation of single crystals of this form, which were successfully obtained (see Experimental Section).

The elucidation of the crystal structure of cocrystals **1** and **2** revealed a 2:1 (HACA:dPy) stoichiometry (dPy = pyridine derivative), whereas cocrystal **3** displayed an uncommon 2:3 stoichiometry. Therefore, we extended our virtual assessment initial guessing including the 2:3 molar ratio for the HACA:4,4′-bipy system rather than considering the most common 1:1 and 2:1 stoichiometries for ascertaining which was the most favorable proportion. Initially, the 1:1 and 2:1 stoichiometries were considered for the three selected combinations, and it was observed that the formation of cocrystals was favored over the obtention of the initial components in both ratios (Δ*E* < 0), with the formation of cocrystals with a 2:1 (HACA:dPy) molar ratio being more probable instead of 1:1 (Δ*E*_2:1 ratio_ < Δ*E*_1:1 ratio_), which was in line with the crystal structures of **1** and **2**. Then, by including the Δ*E* calculation using a 2:3 ratio for the HACA:4,4′-bipy system, it was observed that this combination was slightly thermodynamically favored over the 2:1 stoichiometry (Table 3). However, because several factors can influence the structural outcome,^64−67^ we hypothesize that the 2:3 proportion of **3** has been successfully isolated owing to the change in the synthetic conditions.

**Table 3:** Calculated Energetic Difference in Interaction Site Pairing Energies (kJ/mol) of the Virtual Screening for Possible Combinations of the Selected Components in the Present Work and in Our Previous Contribution Containing Bipyridine-Type Ligands^39^

Component combinations	Δ*E*	Experimental outcome^a^
HACA + 1,2-bpe (1:1)	–3.8	cocrystal 2:1 (**1**)
HACA + 1,2-bpe (2:1)	–7.5	
HACA + 4,4′-azpy (1:1)	–4.0	cocrystal 2:1 (**2**)
HACA + 4,4′-azpy (2:1)	–8.0	
HACA + 4,4′-bipy (1:1)	–4.3	cocrystal 2:3 (**3**)
HACA + 4,4′-bipy (2:1)	–8.5	
HACA + 4,4′-bipy (2:3)	–8.8	
HPip +4,4′-bipy (1:1)	–2.4	cocrystal 1:1^39^
HPip +4,4′-bipy (2:1)	–4.2	
HCinn +4,4′-bipy (1:1)	–1.1	cocrystal 2:1^39^
HCinn +4,4′-bipy (2:1)	–1.2	

a The molar ratio of the experimental outcome column is given in the acid/bipyridine order.

**Figure 1:**
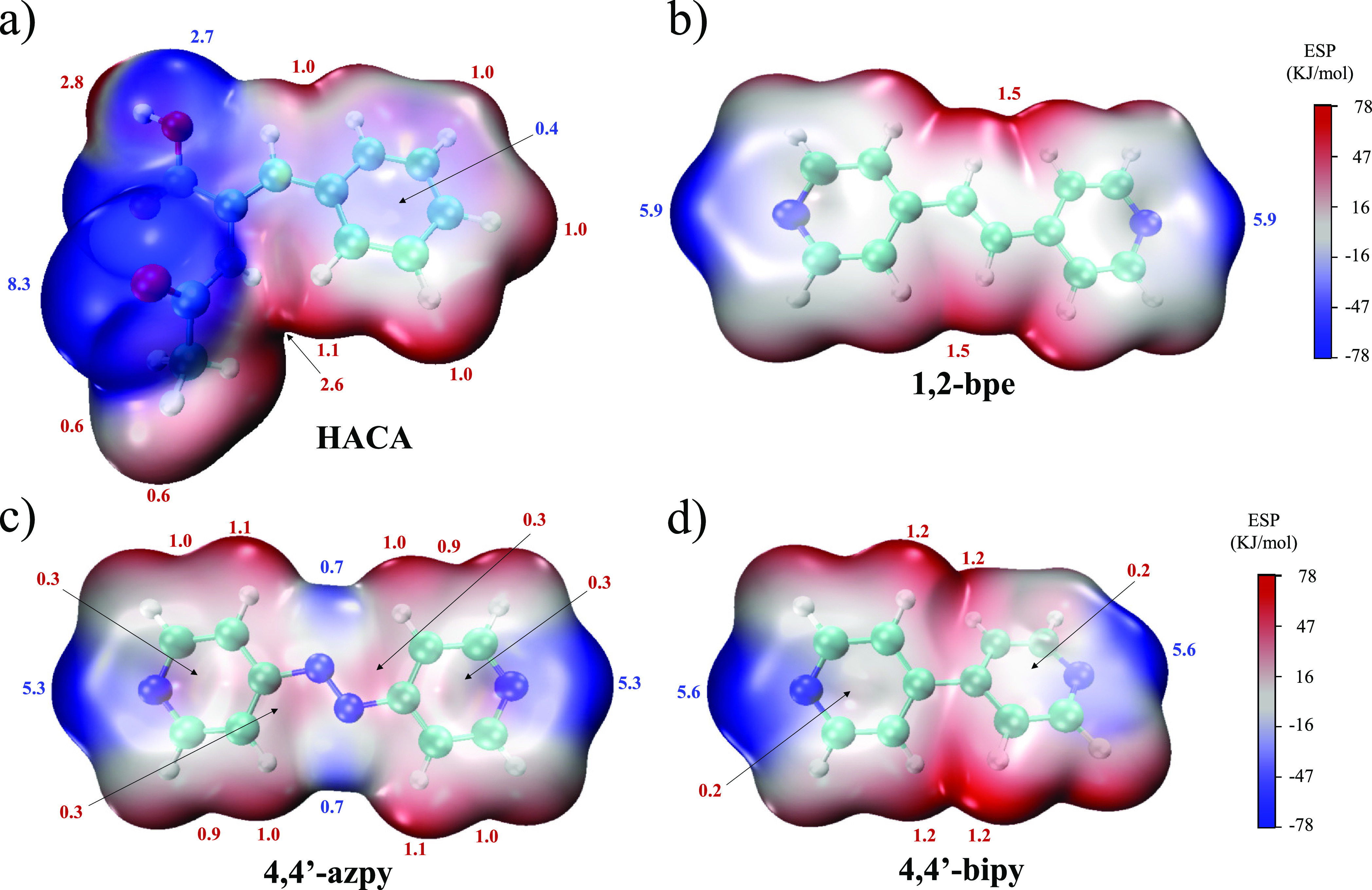
MEP representation of (a) HACA, (b) 1,2-bpe, (c) 4,4′-azpy, and (d) 4,4′-bipy with their α (red) and β (blue) values indicated around each surface.

The comparison of the HACA:4,4′-bipy system with others containing different carboxylic acids and 4,4′-bipy previously reported by us (piperonylic acid, HPip; cinnamic acid, HCinn)^39^ showed that cocrystals based on HACA are thermodynamically favored over HPip and HCinn, following the HACA > HPip > HCinn order, which agrees with the carboxylic acids presenting more H-donors and H-acceptors (Table 3). Furthermore, it was observed that for the combinations present in **1**–**3**, the best H-donor groups corresponded to the carboxylic acid (α = 2.8) and amide (α = 2.6) moieties showing similar H-donor propensities, whereas the best H-acceptor was the carbonyl from the amide group (β = 8.3) followed by the corresponding pyridine groups (β = 5.9, 1,2-bpe; 5.3, 4,4′-azpy; 5.6, 4,4′-bipy) and the carbonyl from the carboxylic acid moiety (β = 2.7) (Figure 1).

Cocrystals **1**–**3** were characterized by powder X-ray diffraction (PXRD); elemental analysis (EA); FTIR-ATR; ^1^H, ^13^C{^1^H}, and DEPT-135 NMR spectroscopies; and single crystal X-ray diffraction method. Phase purity of the ground samples of **1** and **2** and the crystalline powders of HACA, HACA·2H_2_O, and **3** was verified by PXRD (SI Figures S1–S5). The EA agrees with the proposed formulas. The FTIR-ATR spectra presented broad bands attributable to ν_st_(O–H) from HACA in the 2848–2682 cm^–1^ (HACA·2H_2_O), 2669–2145 cm^–1^ (**1**), 2680–2098 cm^–1^ (**2**), and 2685–2141 cm^–1^ (**3**) regions (SI Figures S7–S10), which are shifted to lower wavelengths compared with those of the HACA spectrum (3132–2721 cm^–1^) (SI Figure S6). Furthermore, for the spectrum of HACA·2H_2_O, another broad band appeared in the 3623–2848 cm^–1^ region corresponding to the ν_st_(O–H) from water (SI Figure S7). An additional group of broad bands at 2130–2045 cm^–1^ (HACA·2H_2_O), 2132–1758 cm^–1^ (**1**), 2025–1740 cm^–1^ (**2**), and 2135–1769 cm^–1^ (**3**) suggested the formation of synthons different than the acid···acid homosynthons (2699–2310 cm^–1^ for HACA),^68^ which according to the virtual assessment results should be originated by the formation of acid···pyridine heterosynthons for cocrystals **1**–**3**, whereas for HACA·2H_2_O, they should be related with interactions involving the water molecules, and thus, they have been assigned to the ν_st_(N/O–H···N/O) vibrations.^69^ Furthermore, two types of carbonyl signals have been found, the ν_st_(C=O)_COOH_, which were located at 1683 cm^–1^ (HACA), 1685 cm^–1^ (HACA·2H_2_O), 1687 cm^–1^ (**1**), 1675 cm^–1^ (**2**), and 1711 cm^–1^ (**3**), being in line with the formation of cocrystals (**1**–**3**) and a hydrate (HACA·2H_2_O) instead of salts. Besides, the ν_st_(C=O)_CONH_ were assigned at 1650 cm^–1^ (HACA and HACA·2H_2_O), 1654 cm^–1^ (**1**), 1657 cm^–1^ (**2**), and 1682 cm^–1^ (**3**), suggesting a different behavior of these groups in cocrystal **3** compared with the rest of compounds. Additional signals such as the ν_st_(C=C/C=N) (1640–1593 cm^–1^) or the δ(C=C/C=N) (1421–1264 cm^–1^), δ_ip_(C–H) (1013–907 cm^–1^), and δ_oop_(C–H) (839–688 cm^–1^) bendings have also been found. Further details of the FTIR-ATR spectra and their assignation are provided in the Experimental Section and the SI (Figures S6–S10).

The ^1^H, ^13^C{^1^H}, and DEPT-135 NMR spectra of cocrystals **1**-**3** have been recorded in CD_3_OD. The spectra showed the signals attributable to HACA and their corresponding dPy ligands in the three cocrystals, with a HACA:dPy ratio of 2:1 (**1** and **2**) and 2:3 (**3**) (SI Figures S11–S13). In addition, the ^13^C{^1^H} NMR spectra presented the signals of all the carbon atoms of the corresponding ligands, which have been successfully assigned with the aid of their corresponding DEPT-135 NMR spectra (SI Figures S14–S16).

### Structural Description of HACA and HACA·2H_2_O

**Figure 2:**
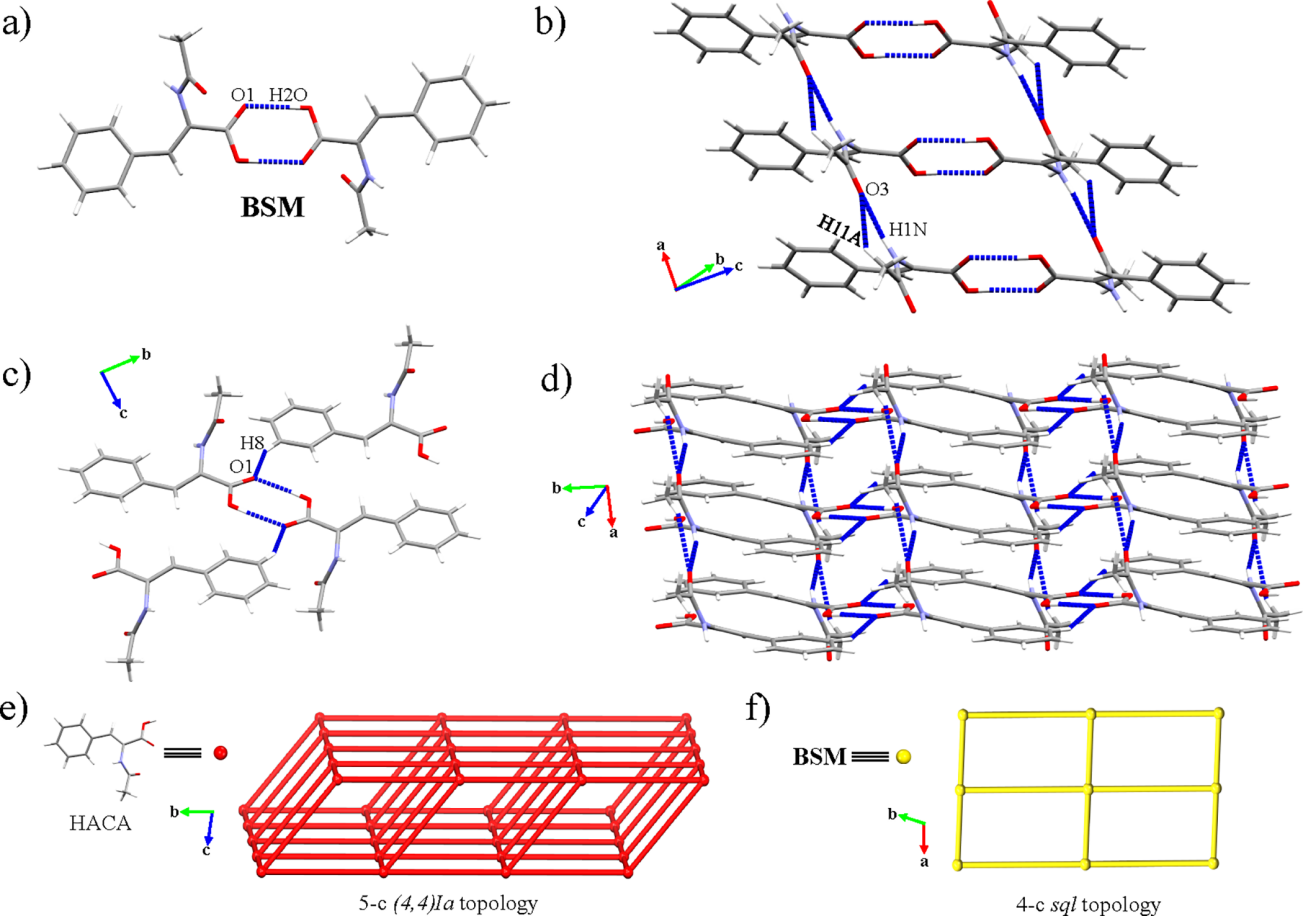
(a) BSM of HACA. (b) 1D chains along the [100] direction. (c) Interactions responsible of the 2D expansion along the (002) plane. (d) General view of the (002) plane. Schematic representation of the topology of the HACA structure considering (e) HACA or (f) their BSMs as nodes.

The crystal structures of HACA and HACA·2H_2_O belong to the triclinic *P*1̅ (HACA) and monoclinic *P*2_1_/*c* (HACA·2H_2_O) space groups. The crystal packing of the HACA form is sustained by acid···acid (O(2)–H(2O)···O(1): 1.78 Å, 176°) and amide···amide (N(1)–H(1N)···O(3): 1.99 Å, 161°) homosynthons as the main interactions (Table 4; SI Figure S17). The acid···acid interactions formed the dimeric basic structural motifs (BSMs) (Figure 2a), whereas the amide···amide synthons, supported by the C(11)–H(11A)···O(3) interaction (2.49 Å, 134°), ordered the BSMs forming double-pillared chains along the [100] direction (Figure 2b). The crystal packing is extended by the C(8)–H(8)···O(1) association (2.50 Å, 146°) between a *m*-H atom and the carboxylate groups (Figure 2c), forming 2D layers with a {4^8^6^2^} point symbol corresponding to a 5-c *(4,4)Ia* underlying topology along the (002) plane (Figure 2d,e), which can be simplified to a 4-c net with a {4^4^6^2^} point symbol corresponding to an a *sql* underlying topology if the BSMs are considered as nodes (Figure 2f).

**Table 4:** Selected Supramolecular Interactions for HACA and HACA·2H_2_O

HACA						
D–H···A	D–H (Å)	H···A (Å)	D···A (Å)	>D-H···A (°)	Associated energy (kJ/mol)	Number of interactions^a^
N(1)–H(1N)···O(3)	0.88	1.99	2.8328(11)	161	–54.4	1
C(11)–H(11A)···O(3)	0.98	2.49	3.2488(13)	134		
O(2)–H(2O)···O(1)	0.84	1.78	2.6208(12)	176	–75.1	2
C(8)–H(8)···O(1)	0.95	2.50	3.3278(14)	146	–14.2	1

HACA·2H_2_O						
D–H···A	D-H (Å)	H···A (Å)	D···A (Å)	>D–H···A (°)	Associated energy (kJ/mol)	Number of interactions^a^
N(1)–H(1)···O(1)	0.88	2.07	2.934(2)	166	–39.7	1
O(1W)–H(1WA)···O(2)	0.90(2)	1.93(2)	2.812(2)	165.5(19)	–20.6	1
O(1W)–H(1WB)···O(3)	0.90(2)	1.77(2)	2.670(2)	178(2)	–25.2	1
O(2)–H(2O)···O(2W)	0.84	1.69	2.515(2)	167	–35.3	1
O(2W)–H(2WA)···O(1W)	0.888(14)	1.885(15)	2.767(2)	172(2)	–25.2	1
O(2W)–H(2WB)···O(1W)	0.89(2)	1.81(2)	2.693(3)	171(3)	–31.1	1
C(3)–H(3)···O(1)	0.95	2.49	3.341(2)	149	–21.7	2
C(5)–H(5)···O(1)	0.95	2.65	3.442(3)	141		
C(7)–H(7)···O(3)	0.95	2.49	3.438(3)	173	–26.0	1*

X–H···Cg(J)	H···Cg(J) (Å)	H-Perp^b^ (Å)	γ^c^ (°)	X···Cg(J) (Å)	X-H, Pi^d^ (°)	Associated energy (kJ/mol)	Number of interactions^a^
C(8)–H(8)···Cg(1)	2.87	2.80	12.71	3.564(3)	48	–26.0	1*

* Indicates the interactions associated with a common interaction energy.

a Number of interactions encompassed in each associated total energy.

b Perpendicular distance of H to ring plane J.

c Angle between the Cg(J)–H vector and ring J normal.

d Angle of the X–H bond with the Pi-plane (perpendicular = 90°, parallel = 0°). HACA·2H_2_O: Cg(1) = C(4) C(5) C(6) C(7) C(8) C(9).

**Figure 3:**
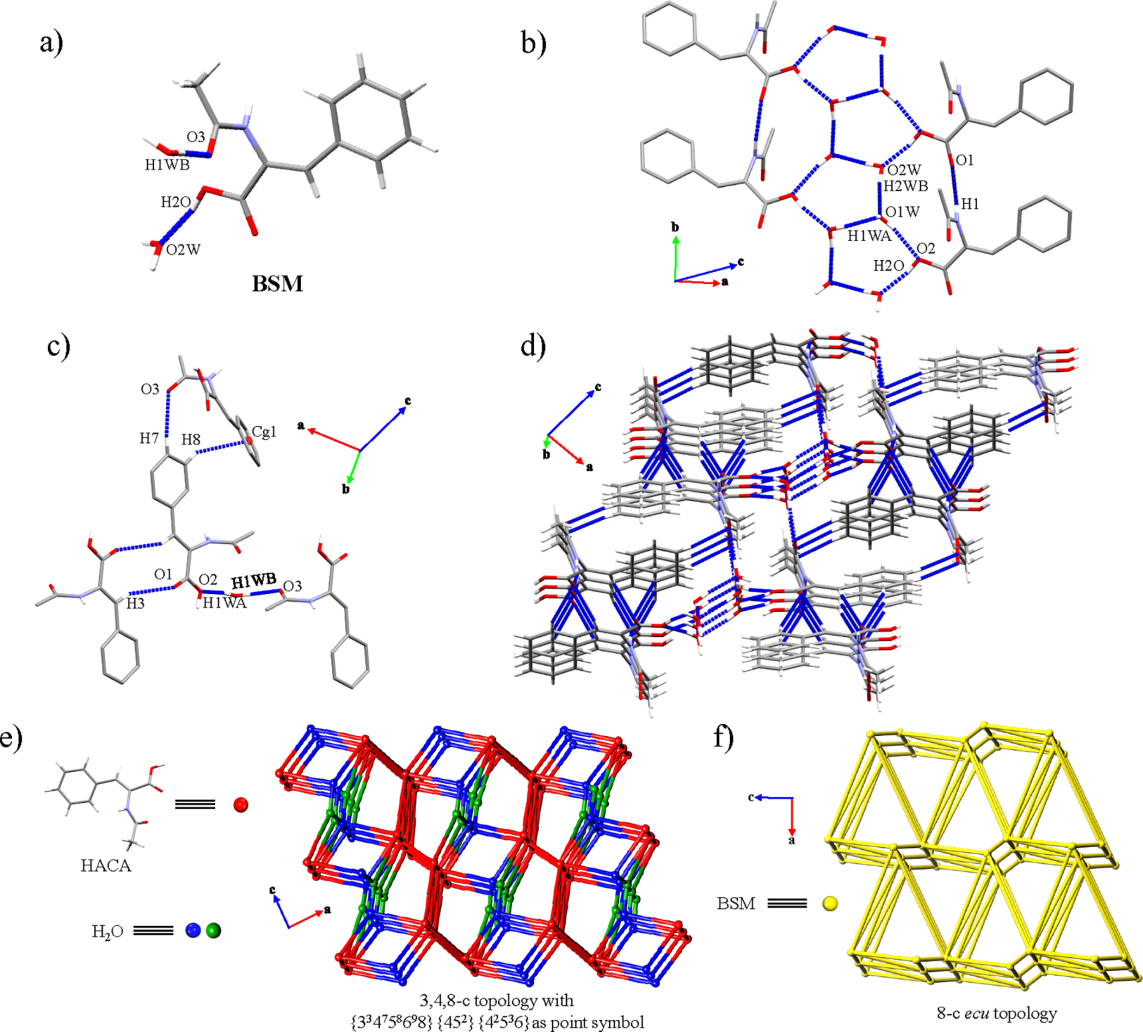
(a) BSM of HACA·2H_2_O. (b) 1D chains along the [010] direction. (c) Interactions responsible of the 3D extension in the HACA·2H_2_O structure. (d) General view of the 3D network of HACA·2H_2_O. Schematic representation of the topology of the HACA·2H_2_O structure considering (e) their former molecules (HACA and H_2_O) and (f) their BSM as nodes. Hydrogen atoms not involved in the highlighted intermolecular interactions have been omitted for clarity in panels (b) and (c).

Otherwise, the introduction of water molecules forming the HACA dihydrate promoted the disruption of the acid···acid homosynthons by the formation of a robust masked synthon^43^ involving water clusters (Table 4; SI Figure S18). Herein, the BSM consisted of trimeric units formed by one HACA and two different water molecules (Figure 3a), which are connected between them, forming 1D channels along the [010] direction (Figure 3b). These channels altered the behavior of the HACA moieties, disrupting not only the acid···acid homosynthon but also the amide···amide homosynthon that is substituted by amide···acid synthons (N(1)–H(1)···O(1): 2.07 Å, 166°) owing to the change of disposition of the HACA molecules toward their association with the water clusters through acid···water and amide···water masked synthons (Table 4, Figure 3c). The interactions involving the water molecules as well as additional C–H···O associations lead to a 3,4,8-c 3D net with {3^3^4^7^5^8^6^9^8}{45^2^}{4^2^5^3^6} as point symbol (Figure 3d,e), whose simplification considering the BSM as a node resulted in a 8-c 3D net with an *ecu* underlying topology (Figure 3f).

### Structural Description of (HACA)_2_(1,2-bpe) (1) and (HACA)_2_(4,4′-azpy) (**2**)

**Figure 4:**
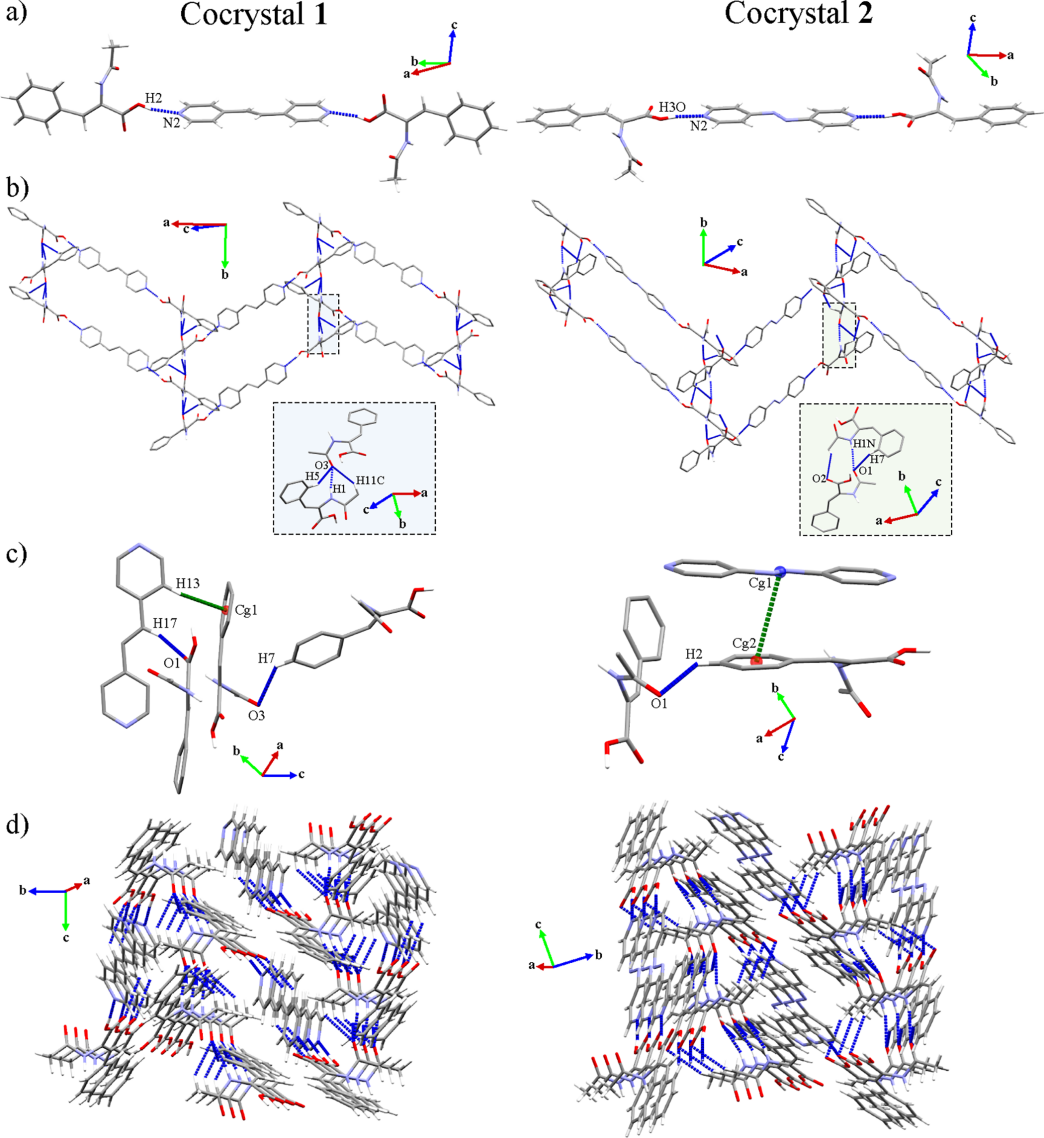
(a) BSMs of cocrystals **1** and **2**. (b) 2D expansion of cocrystals **1** and **2**. (c) Interactions responsible of the 3D expansion in cocrystals **1** and **2**. (d) General view of the 3D networks of cocrystals **1** and **2**. Hydrogen atoms not involved in the highlighted intermolecular interactions have been omitted for clarity in panels (b) and (c).

Cocrystals **1** and **2** belong to the monoclinic *P*2_1_/*c* (**1**) and *P*2_1_/*n* (**2**) space groups. Both consisted of binary cocrystals formed by two HACA and one dPy (1,2-bpe, **1**; 4,4′-azpy, **2**) held together by acid···pyridine heterosynthons (O(2)–H(2)···N(2): 1.79(4) Å, 162(3)° (**1**); O(3)–H(3O)···N(2): 1.72 Å, 172(2)° (**2**)) forming trimeric BSMs (Table 5; Figure 4a). These heterosynthons stand out as the most important connecting agents between the HACA and dPy molecules according to their 2D fingerprint plots (SI Figures S19 and S20). In addition, the position of the HACA molecules with respect to their corresponding dPy in the BSMs showed a remarkable torsion in cocrystal **1** (84.83°) in comparison with cocrystal **2** (22.11°), represented through the angle between the plane of the HACA molecules (green plane) and their corresponding dPy (orange plane) (SI Figure S21). These motifs are extended along the [010] direction through amide···amide homosynthons (N(1)–H(1)···O(3), 1.88 Å, 170° (**1**); N(1)–H(1N)···O(1), 2.063(18) Å, 165.6(16)° (**2**)), presenting a closer interaction length in cocrystal **1** compared with cocrystal **2**. Besides, the 1D expansion is supported by the C(11)–H(11C)···O(3) (2.50 Å, 141°) and C(5)–H(5)···O(3) (2.64 Å, 145°) (**1**), or the C(6)–H(7)···O(1) (2.51 Å, 160°) and C(1)–H(3)···O(2) (2.58 Å, 125°) (**2**) associations. Their 2D fingerprint plots demonstrated these homosynthons as the closer interactions, being the main directors of the crystal packing (SI Figures S19 and S20). Accordingly, these homosynthons orientated the HACA molecules in an alternative sequence, exposing their carboxylic acid moiety at opposite sites with a displacement of 79.68° (**1**) and 71.75° (**2**) between each HACA molecules, which enable the 2D expansion forming herringbone-shaped 2D layers along the (1̅04) (**1**) and (1̅02) (**2**) planes (Figure 4b).

**Table 5:** Selected Supramolecular Interactions for Cocrystals **1** and **2**

**Cocrystal 1**						
D–H···A	D–H (Å)	H···A (Å)	D···A (Å)	>D–H···A (°)	Associated energy (kJ/mol)	Number of interactions^a^
O(2)–H(2)···N(2)	0.88(4)	1.79(4)	2.646(3)	162(3)	–40.7	1
N(1)–H(1)···O(3)	0.88	1.88	2.754(3)	170	–46.2	1
C(11)–H(11C)···O(3)	0.98	2.50	3.325(5)	141		
C(5)–H(5)···O(3)	0.95	2.64	3.463(5)	145		
C(17)–H(17)···O(1)	0.95	2.68	3.438(3)	137	–18.6	1
C(7)–H(7)···O(3)	0.95	2.53	3.268(4)	134	–17.9	1

X–H···Cg(J)	H···Cg(J) (Å)	H-Perp^b^ (Å)	γ^c^ (°)	X···Cg(J) (Å)	X–H, Pi^d^ (°)	Associated energy (kJ/mol)	Number of interactions^a^
C(13)–H(13)···Cg(1)	2.87	2.64	23.22	3.720(5)	70	–20.9	1

**Cocrystal 2**						
D–H···A	D–H (Å)	H···A (Å)	D···A (Å)	>D–H···A (°)	Associated energy (kJ/mol)	Number of interactions^a^
O(3)–H(3O)···N(2)	0.94(3)	1.72(3)	2.6471(18)	172(2)	–46.8	1
N(1)–H(1N)···O(1)	0.882(18)	2.063(18)	2.9254(15)	165.6(16)	–48.0	1
C(6)–H(7)···O(1)	0.95	2.51	3.4118(19)	160		
C(1)–H(3)···O(2)	0.98	2.58	3.2532(19)	125		
C(8)–H(2)···O(1)	0.95	2.56	3.265(2)	132	–17.7	1

Cg(I)···Cg(J)	*d*_Cg-Cg_^e^ (Å)	α^f^ (°)	β, γ^g^ (°)	*d*_plane···plane_^h^ (Å)	*d*_offset_^i^ (Å)	Associated energy (kJ/mol)	Number of interactions^a^
Cg(1)···Cg(2)	3.648	6.0	10.4, 16.4	3.588, 3.499	0.659, 1.032	–28.8	1

a Number of interactions encompassed in each associated total energy.

b Perpendicular distance of H to ring plane J.

c Angle between the Cg(J)–H vector and ring J normal.

d Angle of the X–H bond with the Pi-plane (perpendicular = 90°, parallel = 0°).

e Centroid–centroid distance.

f Dihedral angle between the ring planes.

g Angle between Cg(I)–Cg(J) vector and normal to plane I (β), angle between Cg(I)–Cg(J) vector and normal to plane J (γ).

h Perpendicular distance of Cg(I) on plane J and perpendicular distance of Cg(J) on plane I.

i Horizontal displacement or slippage between Cg(I) and Cg(J). **1**: Cg(1) = C(4) C(5) C(6) C(7) C(8) C(9). **2**: Cg(1) = N(3) N(4); Cg(2) = C(5) C(6) C(7) C(8) C(9) C(10).

**Figure 5:**
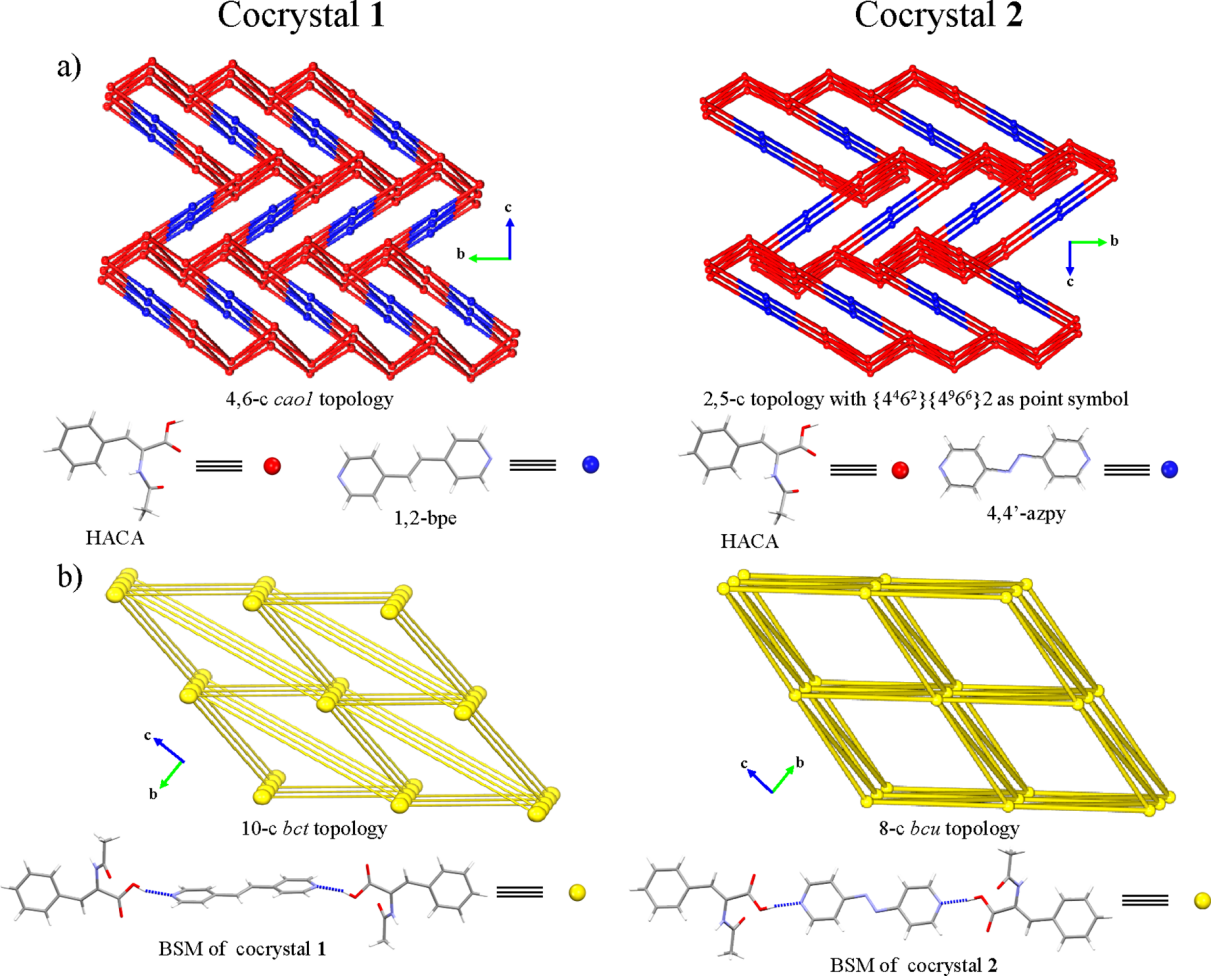
Schematic representation of the topologies of cocrystals **1** and **2** considering (a) their former molecules (HACA and dPy) and (b) their BSMs as nodes.

The crystal packing of cocrystal **1** is completed by the C(17)–H(17)···O(1) (2.68 Å, 137°) and C(7)–H(7)···O(3) (2.53 Å, 134°) associations (Figure 4c), which allow the 3D expansion of the structure leading to a 4,6-c binodal net corresponding to a *cao1* underlying topology, supported by C–H···π interactions (C(13)–H(13)···Cg(1): 2.87 Å) (Table 5, Figures 4d and 5a), which are also suggested by the flat regions of the curvedness representation of the HACA molecules (SI Figure 19b). Otherwise, the C(8)–H(2)···O(1) (2.56 Å, 132°) interaction in cocrystal **2** extended its 3D network forming a 2,5-c binodal net with {4^4^6^2^8^4^}2{8} as point symbol, sustained by azo···π interactions (Cg(1)···Cg(2): 3.648 Å)^70^ (Table 5, Figures 4c,d and 5a), being in agreement with the flat regions of the HACA and 4,4′-azpy regions in their corresponding curvedness representations (SI Figure S20b,e). Further simplification of their topologies considering the BSMs as nodes leads to a 10-c *bct* (**1**) and 8-c *bcu* (**2**) underlying topologies (Figure 5b).

### Structural Description of (HACA)_2_(4,4′-bipy)_3_ (**3**)

**Figure 6:**
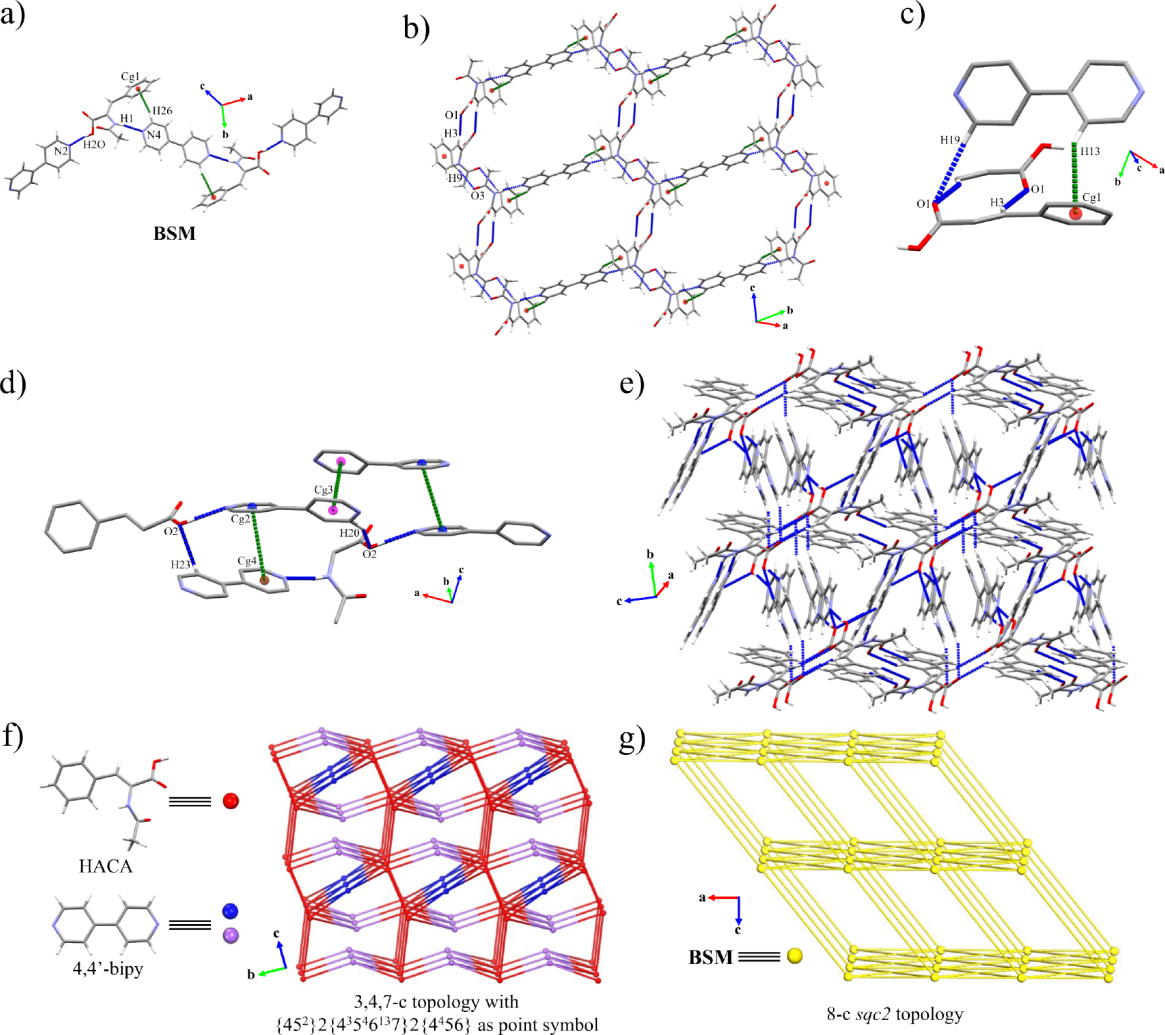
(a) BSM of cocrystal **3**. (b) 2D expansion of cocrystals **3** along the (11̅0) plane. (c, d) Interactions responsible of the 3D extension in cocrystals **3**. (e) General view of the 3D network of cocrystal **3**. Schematic representation of the topology of cocrystal **3** considering (f) their former molecule (HACA and 4,4′-bipy) and (g) their BSMs as nodes. Hydrogen atoms not involved in the highlighted intermolecular interactions have been omitted for clarity in panels (c) and (d).

Cocrystal **3** belongs to the triclinic *P*1̅ space group. It consisted of a binary cocrystal formed by two HACA and three 4,4′-bipy molecules constructing a pentameric array as BSM. The 4,4′-bipy molecules displayed two different behaviors, two of them forming a single acid···pyridine heterosynthon (O(2)–H(2O)···N(2): 1.78 Å, 168°) with a torsion angle of 35.23° between their aromatic rings, whereas the remaining one keeps its torsion angle completely plane, forming a double amide···pyridine heterosynthon (N(1)–H(1)···N(4): 2.07 Å, 170°) supported by C–H···π associations (C(26)–H(26)···Cg(1): 2.63 Å) (Table 6; Figure 6a). The Hirshfeld surface of the former components and their corresponding 2D fingerprint plots showed the acid···pyridine and amide···pyridine synthons as the main contributors to the assembly of the cocrystal (SI Figure S22). The BSMs are connected by C–H···O interactions between nearby HACA molecules forming 2D honeycomb layers along the (11̅0) plane (Figure 6b). The 3D expansion of the cocrystal is completed by additional C–H···O as well as multiple π···π and C–H···π interactions involving the 4,4′-bipy molecules (Table 6, Figure 6c–e, SI Figure S22), resulting in a 3,4,7-c net with {34^3^5^2^}2{3^2^4^6^5^6^6^13^7}2{4^4^56} as point symbol (Figure 6f), whose simplification considering the BSM as a single node led to a 10-c net corresponding to an *sqc2* underlying topology (Figure 6g).

**Table 6:** Selected Supramolecular Interactions for Cocrystal **3**

D–H···A	D–H (Å)	H···A (Å)	D···A (Å)	>D–H···A (°)	Associated energy (kJ/mol)	Number of interactions^a^
O(2)–H(2O)···N(2)	0.84	1.78	2.607(2)	168	–40.4	1
N(1)–H(1)···N(4)	0.88	2.07	2.939(2)	170	–44.8	1*
C(9)–H(9)···O(3)	0.95	2.49	3.335(2)	148	–52.2	2
C(3)–H(3)···O(1)	0.95	2.48	3.352(2)	153	–27.9	2
C(19)–H(19)···O(1)	0.95	2.52	3.437(3)	162	–23.3	2*
C(20)–H(20)···O(2)	0.95	2.51	3.339(3)	146	–12.5	1
C(23)–H(23)···O(2)	0.95	2.53	3.350(3)	144	–20.9	1

X–H···Cg(J)	H···Cg(J) (Å)	H-Perp^b^ (Å)	γ^c^ (°)	X···Cg(J) (Å)	X–H, Pi^d^ (°)	Associated energy (kJ/mol)	Number of interactions^a^
C(13)–H(13)···Cg(1)	2.74	2.72	7.09	3.5889(19)	65	–23.3	2*
C(26)–H(26)···Cg(1)	2.63	2.60	8.00	3.530(2)	61	–44.8	1*

Cg(I)···Cg(J)	*d*_Cg-Cg_^e^ (Å)	α^f^ (°)	β, γ^g^ (°)	*d*_plane···plane_^h^ (Å)	*d*_offset_^i^ (Å)	Associated energy (kJ/mol)	Number of interactions^a^
Cg(2)···Cg(2)	3.7208(11)	0.02(9)	16.5	3.5671(7)	1.058	–16.9	1
Cg(3)···Cg(3)	3.7840(11)	0.03(9)	22.9	3.4866(8)	1.470	–25.6	1
Cg(2)···Cg(4)	3.8540(12)	23.61(10)	7.8, 28.6	3.3826(7), 3.8181(10)	0.523, 1.845	–25.0	1

* Indicate the interactions associated with a common interaction energy.

a Number of interactions encompassed in each associated total energy.

b Perpendicular distance of H to ring plane J.

c Angle between the Cg(J)–H vector and ring J normal.

d Angle of the the X–H bond with the Pi-plane (perpendicular = 90°, parallel = 0°).

e Centroid–centroid distance.

f Dihedral angle between the ring planes.

g Angle between the Cg(I)–Cg(J) vector and normal to plane I (β); angle between the Cg(I)–Cg(J) vector and normal to plane J (γ).

h Perpendicular distance of Cg(I) on plane J and perpendicular distance of Cg(J) on plane I.

i Horizontal displacement or slippage between Cg(I) and Cg(J). Cg(1) = C(4) C(5) C(6) C(7) C(8) C(9); Cg(2) = N(2) C(12) C(13) C(14) C(15) C(16); Cg(3) = N(3) C(17) C(18) C(19) C(20) C(21); Cg(4) = N(4) C(22) C(23) C(24) C(25) C(26).

### Synthon Competitivity between Acid and Amide Groups in Bipyridine-Based Cocrystals

**Figure 7:**
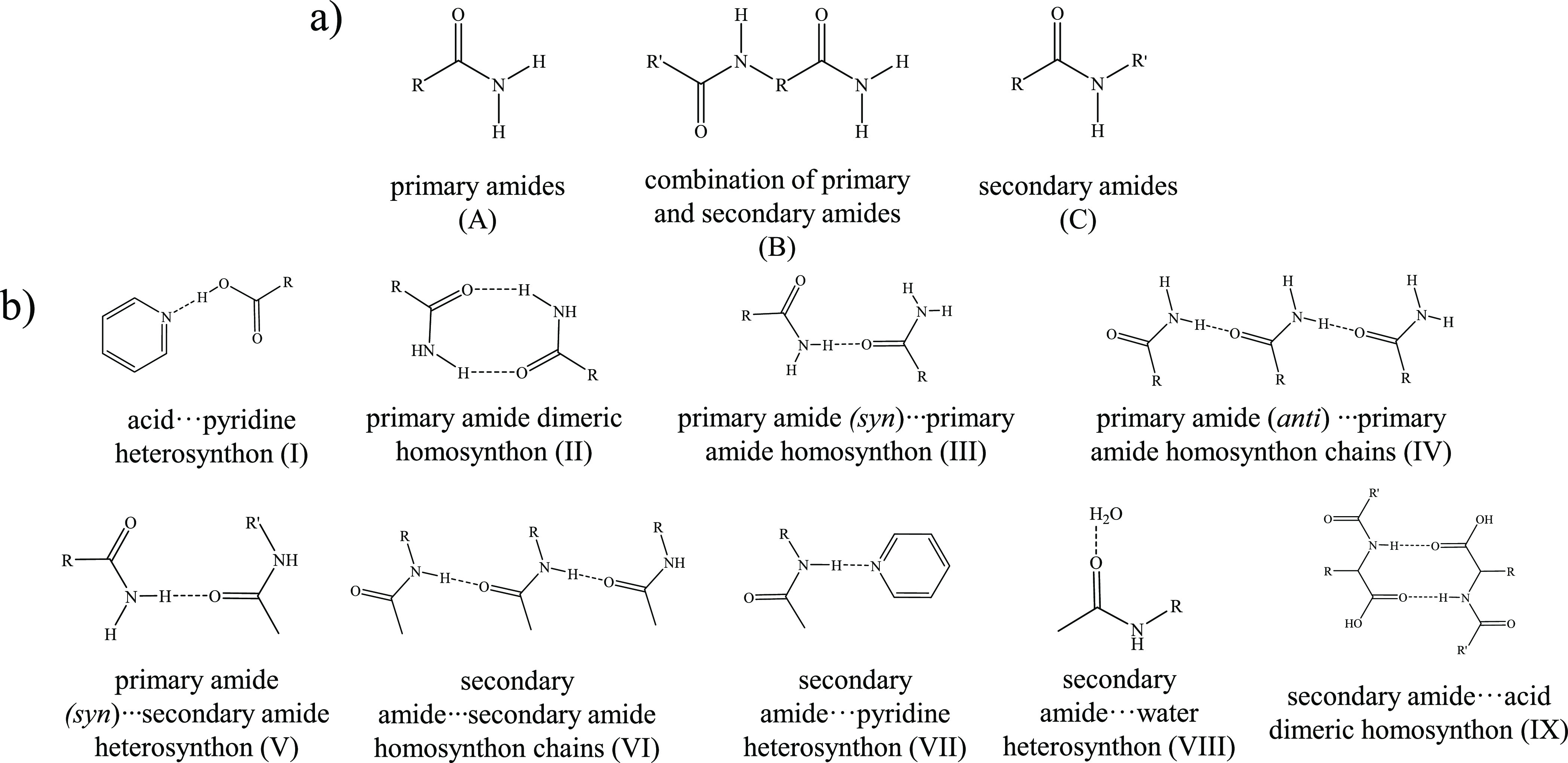
(a) Classification of the hits and (b) types of synthons found for the CSD study.

Aiming to evaluate the synthon competitivity between acid and amide groups with pyridine moieties, we have used the Cambridge Structural Database (CSD)^71^ to search the cocrystal forms containing the following premises: (i) one component with at least an acid and an amide (primary or secondary) groups within the same molecule and (ii) a bipyridine type coformer. This search has been applied to four of the most used bipyridine coformers, including those utilized in this work (1,2-bpe, 4,4′-azpy, 4,4′-bipy, and 1,2-*bis*(4-pyridyl)ethane), yielding 13 hits including cocrystals **1**–**3**. We classified the resulting hits into three groups divided into (A) primary amides (1 hit), (B) combination of primary and secondary amides (1 hit), and (C) secondary amides (11 hits) (Figure 7a). The complete information regarding all the results of this search is provided in the SI (Table S1).

It is worthwhile to mention that when studying the synthon reliability in complex systems, several factors must be considered.^72−75^ However, the general effect of most of them is the modulation of the H-donor/acceptor capability, which we will measure using the α (H-donor propensity) and β (H-acceptor propensity) values. Thus, similar H-donor propensities should result in a possible synthon competitivity, whereas an important disparity should produce supramolecular orthogonality of their synthons. All the MEP surfaces of the acid and bipyridine molecules of this study are provided in the SI (Figures S23 and S24), whereas their relevant α and β values are provided in Table 7.

**Table 7:**
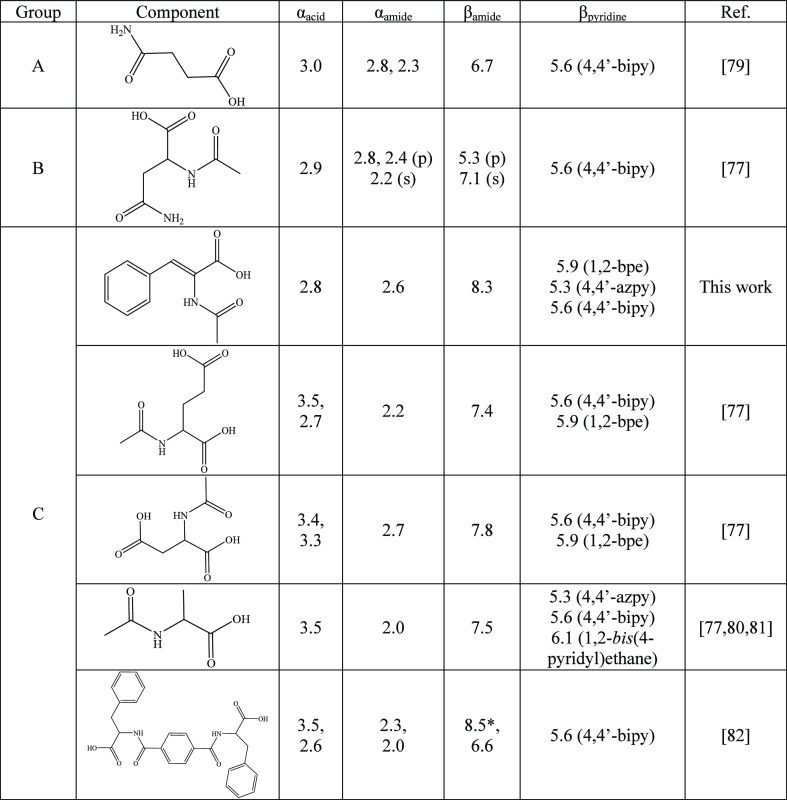
Relevant α and β Values of the Cocrystal Formers of the Literature Containing an Acid and an Amide Group within the Same Molecule^79−82^

Focusing on group C molecules, they showed variable behaviors such as supramolecular chains by synthon type VI (36% of hits), amide···pyridine heterosynthons through synthon type VII (27% of hits), or dimeric motifs between secondary amides and carboxylic acids *via* synthon type IX (36% of hits) (Figure 7b; SI: Table S1). Remarkably, four of the carboxylic acids of this group of molecules presented acetamide moieties in their α position, and it seems that the remaining substituents of this position modulate their H-donor ability (α value). The more electron-withdrawing are the remaining substituents, the lower is the α value of the carboxylic acid. Whereas for aliphatic substituents the effect is poor (α = 3.4–3.5 for R = −CH_3_, −CH_2_COOH, and −CH_2_CH_2_COOH), in the case of a conjugated system such in HACA (where R = CHPh), the H-donor capability of the carboxylic acid gets substantially reduced (α = 2.8). Besides, the components presenting additional carboxylic acid groups also showed a modulation of the second acidic α value relative to their substituent effects. Furthermore, the influence of either the H-donor or the H-acceptor sites of the acetamide moieties is notably influenced by their surrounding H-donor/acceptor groups.^76^ For the H-donor groups, the ability to adopt a conformation where the NH group avoids close contact with the C=O group from the carboxylic acid conferred a slightly better H-donor propensity. At the same time, molecules adopting conformations that enhance the α value of the amidic NH group locate their C=O moiety near the C=O groups from the carboxylic acids, displaying better H-acceptor ability values (β values). This is observed in the structures reported in this work with HACA (α = 2.6, β = 8.3), as well as those with *N*-acetylaspartic acid (α = 2.7, β = 7.8).^77^

After analyzing all the hits, the CSD study showed that secondary amides presented H-donor propensity more modulable than primary amides, being able to present amidic H-donor groups with strengths comparable to them depending on their substituents and environments (Table 7). Accordingly, components presenting more differentiated hierarchies of their H-donor strengths of the acid and amide groups undergo supramolecular chains (type VI) probably driven by polarization effects due to the cooperation of secondary amide interactions^78^ or dimeric (type IX) motifs, whereas those with similar α values (HACA and *N*-acetylaspartic acid) have access to the formation of amide···pyridine associations (type VII) (Figure 7b). The use of components following the strategy reported herein can provide insights toward the preparation of new supramolecular materials with orthogonally assembled synthons, as well as help in the study of supramolecular systems with competitive synthons and the discovery of new recurrent associations.

### Thermal Properties

The thermal behavior of HACA·2H_2_O and cocrystals **1**–**3** was investigated using simultaneous TG/DTA. The thermogram of HACA·2H_2_O showed the loss of two water molecules between 57 and 113 °C followed by a thermally stable region until its final melt degradation, evinced by the endothermic event observed at *T*_onset_ = 184.5 °C and *T*_peak_ = 193.3 °C (SI Figure S25). The three cocrystals also displayed an endothermic event corresponding to their melt degradation following the **1** (*T*_onset_ = 187.8 °C; *T*_peak_ = 195.9 °C) > **2** (*T*_onset_ = 170.9 °C; *T*_peak_ = 177.4 °C) > **3** (*T*_onset_ = 164.9 °C; *T*_peak_ = 170.3 °C) order (SI Figures S26–S28). Besides, in the thermogram of cocrystal **2**, an exothermic event is observed at 188.1 °C after the melting of the product, which is tentatively attributed to the crystallization of a different phase.^83,84^ These values are in line with those determined by the melting point apparatus (189–190 °C, HACA·2H_2_O; 194–195 °C, **1**; 180–181 °C, **2**; 169–170 °C, **3**). After these processes, HACA·2H_2_O and the three cocrystals displayed a continuous loss of mass of all their components with no clear evidence of the decomposition of one component before the other. The comparison of the resulting melting points of cocrystals **1**–**3** with their former components showed that **1** presented a higher melting point than both of its components, which is the less common case, whereas the melting point of cocrystals **2** and **3** lies in between their coformers, as is usual (SI Table S2).^85,86^

To rationalize the thermal stabilities of **1**–**3**, we used energy framework calculations to find the lattice energy (*E*_latt_) of the cocrystals, as well as the energetic components (electrostatic, *E*_ele_; polarization, *E*_pol_; dispersion, *E*_dis_; and repulsion, *E*_rep_) of the different supramolecular interactions forming the crystal packing.^87^ In addition, we have included HACA and HACA·2H_2_O forms to compare with cocrystals **1**–**3**. For the selection of the threshold, we followed the same methodology of our recent work,^40^ which drove us to choose a cutoff energy of −20 kJ/mol (SI Figure S29). Only the interactions above this threshold will be considered because weaker interactions can undergo ruptures or motions when temperature is applied even though the solid state is preserved.^88,89^

From the compiled data, we found that the *E*_latt_ values are not directly correlated with the melting points within the HACA landscape (SI Table S3 and Figure S30). Therefore, we considered individually the total energies (*E*_tot_) of the stronger supramolecular interactions above the selected threshold, bearing in mind their structural parameters^90^ and the directionality provided by themselves (supramolecular cycles, 0D; chains, 1D; or complementary interactions of one of these two types) (Table 8).

**Table 8:** Relevant Information for the Understanding of the Structure–Thermal Stability Correlation^a^

Component	Specific interaction	Propagation of the interaction	H···A	>D–H···A	*E*	Dimensionality^e^
HACA	acid···acid	0D	1.78	176	–75.1^b^	1D
	amide···amide	1D	1.99	161	–54.4^c^	
HACA·2H_2_O	acid···water	0D	1.69	167	–35.3	2D
	acid···water	0D	1.93	166	–20.6	
	amide···water	0D	1.77	178	–25.2	
	amide···acid	1D	2.07	166	–39.7	
	water···water	1D	1.81	171	–31.1	
			1.89	172	–25.1	
Cocrystal **1**	acid···pyridine	0D	1.79	162	–40.7	2D
	amide···amide	1D	1.88	170	–46.2^d^	
Cocrystal **2**	acid···pyridine	0D	1.72	172	–46.8	2D
	amide···amide	1D	2.06	166	–48.0^d^	
Cocrystal **3**	acid···pyridine	0D	1.78	168	–40.4	weak 3D (strong 0D pentameric unit)
	amide···pyridine	0D	2.07	170	–44.8^c^	
	complementary C–H···O	0D	2.49	148	–52.2^b^	
			2.48	153	–27.9^b^	
			2.52	162	–23.3	
			2.53	144	–20.9	

a Interaction lengths are given in Å, angles in °, and energies in kJ/mol.

b Interaction energy associated with reciprocal interactions (both interactions are considered).

c Interaction energy associated with the mentioned interactions and an additional weak interaction.

d Interaction energy associated with the mentioned interactions and two additional weak interactions.

e Dimensionality considering the interactions above the selected energetic threshold.

**Figure 8:**
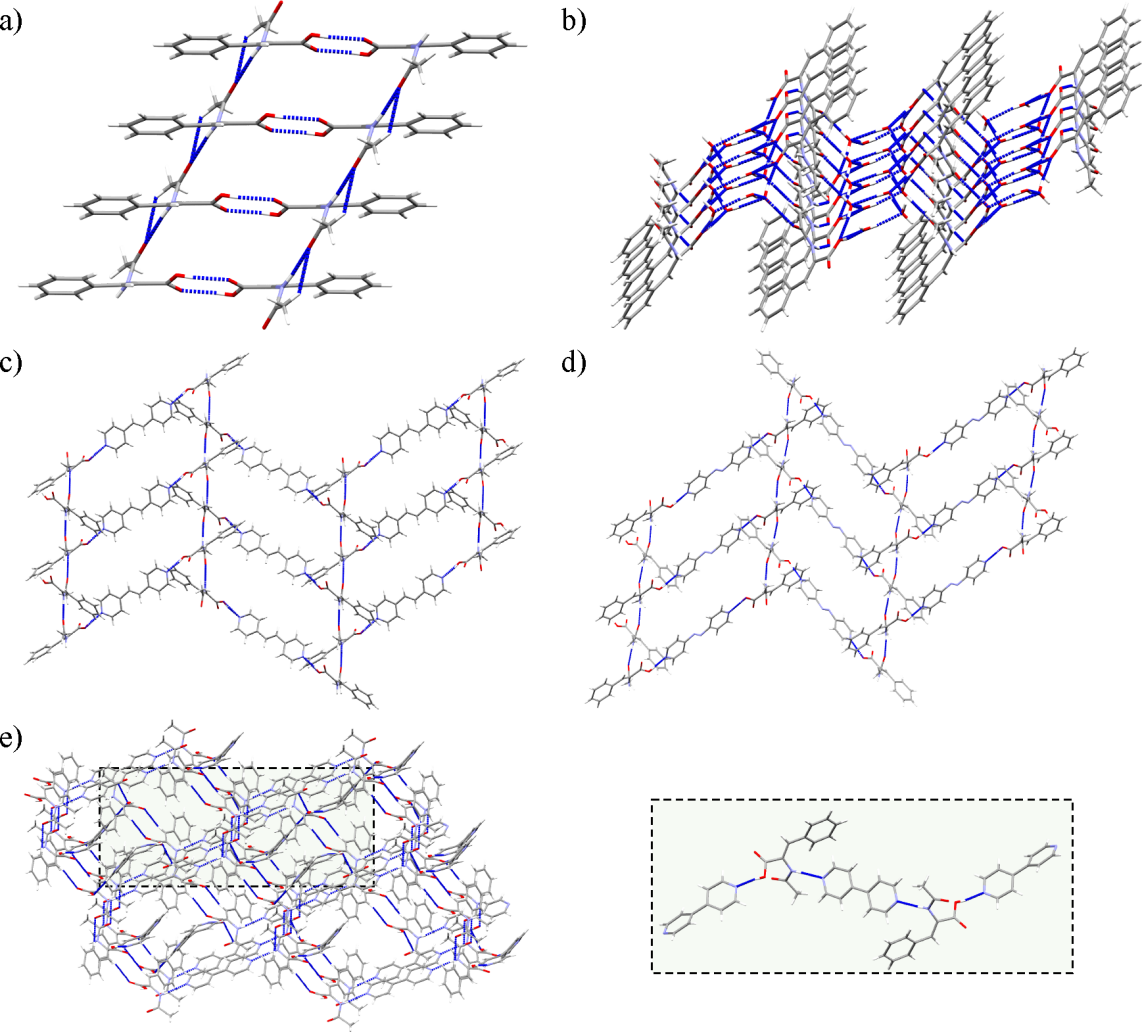
Crystal packing of (a) HACA, (b) HACA·2H_2_O, cocrystals, (c) **1**, (d) **2**, and (e) **3** considering the interactions above the energetic threshold of −20 kJ/mol. Panel (e) contains a detailed view of the main 0D pentameric unit of cocrystal **3** highlighted in green.

We noticed that the different synthons involving the amide moieties, which are responsible for the stronger interactions leading to an extension of the framework, fitted better with the obtained melting point values. The structure of HACA presenting a melting point of 189–190 °C is mainly held together by the acid···acid and amide···amide homosynthons arranging 1D chains (Figure 8a). Conversely, in the structure of HACA·2H_2_O, the previous synthons are disrupted toward the formation of amide···acid synthons and other interactions involving the water molecules, showing a lower strength compared with HACA. Thus, the packing of HACA·2H_2_O leads to 2D layers owing to the directionality of its interactions, consisting of 1D chains mainly formed by the amide···acid synthon joined together by interactions involving water molecules, which form intermediate water cluster chains (Figure 8b). However, it should be considered that cocrystals and pure components are usually more thermally stable than solvates,^91−93^ which is supported by the loss of the two water molecules observed in the thermogram of HACA·2H_2_O (SI Figure S25). Therefore, these 2D layers should be easily disassembled before the melting temperature, leading to 1D chains arranged by amide···acid synthons, which could explain the similar melting points between HACA and HACA·2H_2_O.

When the structure–thermal stability relationship of cocrystals **1**–**3** was analyzed, a clear difference between cocrystals **1** and **2** with respect to **3** in terms of crystal packing was observed. Cocrystals **1** and **2** formed herringbone-shaped 2D layers arranged by acid···pyridine and amide···amide synthons (Figure 8c,d), whereas in cocrystal **3**, the formation of the uncommon amide···pyridine synthon promotes the formation of a pentameric 0D cluster, which is extended to an interpenetrated weak 3D network by C–H···O associations (Table 8; Figure 8e). Herein, the difference between cocrystals **1** and **2** seems to be well correlated with the strength of their amide···amide synthons, being in line with the H-acceptor distance and directionality of these interactions (Table 8),^94−96^ whereas the change of packing of cocrystal **3** to a reduced 0D arrangement extended by weak C–H···O interactions^90^ combined with the longer interactions promoted by the amide···amide synthons agrees with the obtention of the lowest melting point for **3**(39,97,98) (Table 8). Thus, it seems that control of the amide···amide interactions and the crystal packing is an efficient way to tune the thermal stability of HACA-based cocrystals.

### Photophysical Properties

**Figure 9:**
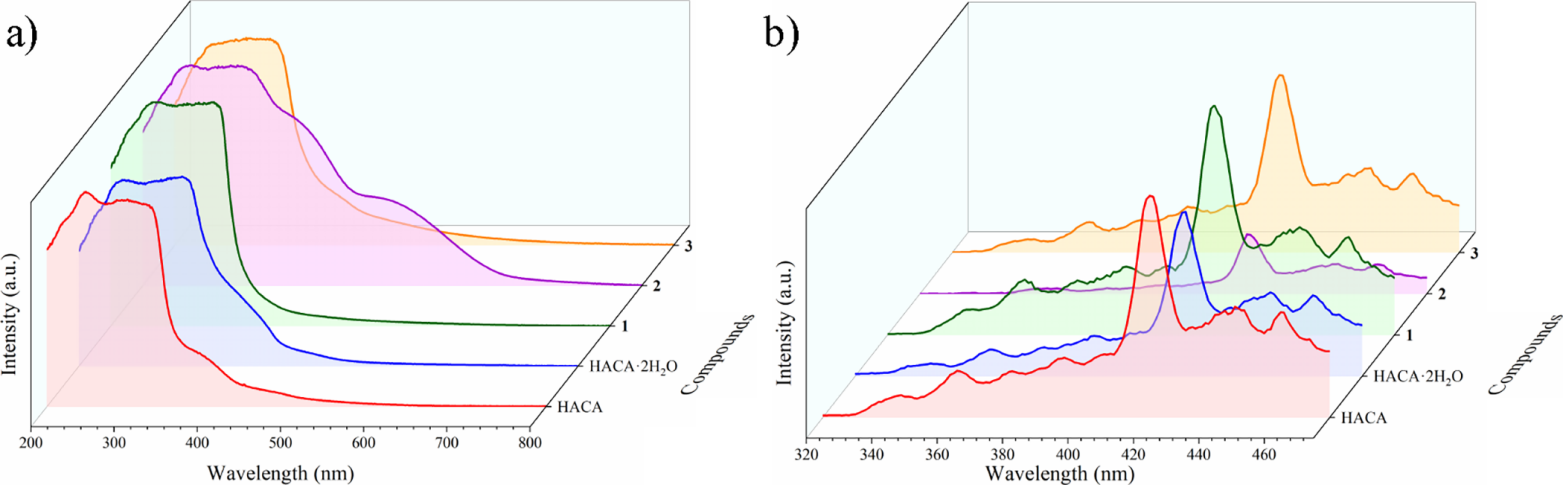
(a) Solid-state UV–vis and (b) solid-state fluorescence irradiated at λ_exc_ = 250 nm of HACA, HACA·2H_2_O, and cocrystals **1**–**3**.

Solid-state UV–vis and fluorescence measurements of HACA, HACA·2H_2_O, and cocrystals **1**–**3** have been recorded. The absorption spectra showed overlapped broad bands with maximums at 246 and 294 nm (HACA), 252 and 324 nm (HACA·2H_2_O), 252 and 322 nm (**1**), 259 and 314 nm (**2**), and 286 nm (**3**). In addition, the spectra of HACA, HACA·2H_2_O, and **2** displayed additional bands around 390 nm (HACA), 400 nm (HACA·2H_2_O), and 390 and 492 nm (**2**) (Figure 9a; SI Table S4). It seems that all the bands corresponded to combinations of absorptions of the π–π* transitions of the former components,^99^ with the exception of the band of 492 nm of **2** that has been assigned to the n−π* transition of the azo chromophore of 4,4′-azpy.^100^ The comparison of HACA and HACA·2H_2_O absorption spectra showed a slight bathochromic shift of HACA·2H_2_O with respect to HACA (SI Figure 31a), as well as for cocrystals **1**–**3** when compared with their pure components (SI Figure 31b–d). Only the band attributed to the azo chromophore of **2** (492 nm) presented a remarkable redshift compared with the same group in 4,4′-azpy (460 nm) (SI: Figure S31c), which should be associated with the double azo···π interactions present in **2**.^101^ Besides, it seems that the different packings of the five crystalline forms allow some modulation of their charge transfers (CTs) following the **1** < HACA < **3** < HACA·2H_2_O < **2** order according to the UV–vis spectra^102^ (Figure 9a).

When the compounds were irradiated at λ_exc_ = 250 nm, all of them showed the same unstructured pattern of bands with a main maximum centered at 420 nm (electric violet color according to the CIE 1931 chromaticity diagram) (SI Figure S32), displaying a 16190 cm^–1^ Stokes shift, which suggests that the fluorescence mainly arises from the HACA component. However, the intensity of the emission spectra is modulated depending on the crystalline form, following the **1** > HACA > **3** > HACA·2H_2_O > **2** order of intensity, being inversely proportional to their degree of CTs (Figure 9b). In addition, it has been reported that when the absorption and emission of a material are overlapped, the quenching of fluorescence is more probable to occur.^103,104^ Therefore, we suggest that in these HACA-based organic systems, the broader is the absorption, the more hindered is the fluorescence emission, which is in line with the overlapped areas between the absorption and emission spectra of the five crystalline forms (SI Figure S33). Thus, we have showed how by constructing different supramolecular assemblies containing the HACA ligand and different bipyridine-based coformers, their CTs can be modulated, affecting their fluorescence intensities.

## Conclusions

Different HACA-based crystalline forms have been successfully prepared and characterized by analytical and spectroscopic techniques. They consisted of the crystalline form of the HACA ligand, their dihydrate (HACA·2H_2_O), and three cocrystals bearing different bipyridine-type coformers ((HACA)_2_(1,2-bpe) (**1**), (HACA)_2_(4,4′-azpy) (**2**), and (HACA)_2_(4,4′-bipy)_3_ (**3**)). The cocrystal forms were initially subjected into a virtual screening methodology using the MEP surfaces of the selected components to ascertain the feasibility of cocrystals formation. Then, they were prepared using LAG (**1** and **2**) or solvothermal techniques (**3**). The elucidation of their crystal structures revealed that the HACA structural landscape showed a synthon competition between the most common acid···pyridine and an uncommon acid···amide synthon, which can be accessed by modifying the synthetic conditions. Cocrystals **1** and **2** showed acid···pyridine and amide···amide synthons that resulted in trimeric BSMs forming strong herringbone-shaped 2D layers extended to 3D networks by weak interactions, whereas for cocrystal **3**, the amide···pyridine combined with acid···pyridine synthons were formed, arranging pentameric BSMs packed into a 3D network by C–H···O associations. Besides, we analyzed the synthon competitivity in cocrystals bearing the most common bipyridine-type coformers with molecules containing acid and amide functionalities using the CSD database, which demonstrates the unusual synthon behavior of cocrystal **3**. In addition, we observed the high versatility of secondary amides and the different synthon outcomes as well as their occurrences, showing the possible modulation of their synthons by tuning the H-donor/H-acceptor capabilities of the acid and amide groups. Finally, the thermal and photophysical properties of the five crystalline forms were studied and correlated with their structural features, and we observed (i) a relationship between the amide synthon strengths and the thermal stability of the products and (ii) a relationship between the degree of CTs of the crystalline forms modulated by the different crystal packings and their fluorescence intensities. This study provides new insights into the possible synthon occurrences of cocrystals and crystalline forms containing carboxylic acids and secondary amides and their effect on the thermal and photophysical properties, which can be useful for extrapolating to other relevant molecules, with special focus to acetylated amino acids due to their structural similarities.

## Supporting Information Available

Additional details about the PXRD diffractograms, FTIR-ATR, ^1^H NMR, ^13^C{^1^H} NMR, DEPT-135 NMR spectra, TG/DTA thermograms, and Hirshfeld surface and energy frameworks analysis of HACA, HACA·2H_2_O, and cocrystals **1**–**3**, as well as complete information about the CSD search and the thermal and photophysical properties of the five crystalline forms (PDF)
