# Correction to “Structural Landscape of α-Acetamidocinnamic Acid Cocrystals with Bipyridine-Based Coformers: Influence of Crystal Packing on Their Thermal and Photophysical Properties”

**DOI:** 10.1021/acs.cgd.4c00270

**Published:** 2024-03-12

**Authors:** Daniel Ejarque, Teresa Calvet, Mercè Font-Bardia, Josefina Pons

The original version of the article contained an erroneously assigned topology for cocrystal (HACA)_2_(4,4′-azpy) (**2**) (HACA = α-acetamidocinnamic acid, 4,4′-azpy = 4,4′-azopyridine) when their molecules were considered as nodes. This affected the structural description of cocrystal **2** (page 1754) and its corresponding figure (Figure 5, page 1756), as well as the X-ray crystallographic data, which was updated with the last version of the ToposPro program and the TopCryst webpage (page 1749). In addition, the acknowledgments were modified after a helpful discussion with Prof. Davide M. Proserpio which led us to realize the mistakes corrected herein (page 1762). The corrections of the article are shown below.

**Figure 5:**
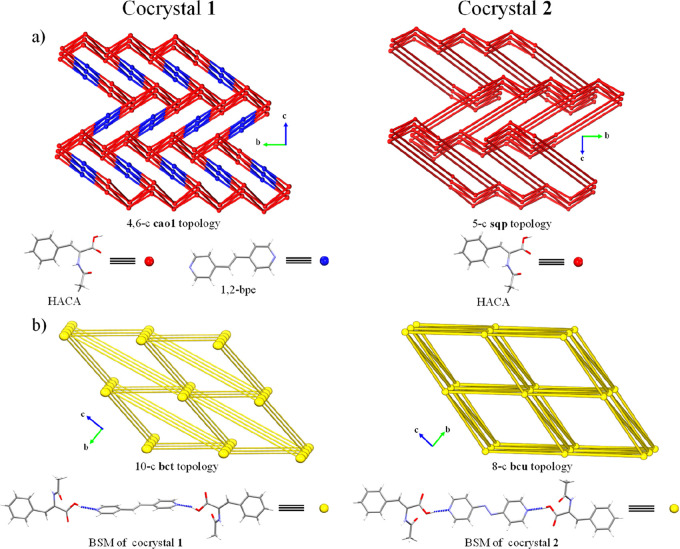
Schematic representation of the topologies of cocrystals **1** and **2** considering (a) their former molecules (HACA and dPy) and (b) their BSMs as nodes. The 4,4′-azpy molecules from cocrystal **2** have been simplified in panel (a) due to the obtention of 2-c nodes which should be removed according to the methodology of reference 52 of the original paper.

**X-ray Crystallographic Data.**

The topological analysis was done employing the ToposPro 5.5.2.1 program and the TopCryst Web site (https://www.topcryst.com/).

**Structural Description of (HACA)_2_(1,2-bpe) (1) and (HACA)_2_(4,4′-azpy) (2).**

Otherwise, the C(8)–H(2)···O(1) (2.56 Å, 132°) interaction in cocrystal **2** extended its 3D network forming a 5-c **sqp** underlying topology, sustained by azo···π interactions (Cg(1)···Cg(2): 3.648 Å)^70^ (Table 5, Figures 4c,d and 5a), being in agreement with the flat regions of the HACA and 4,4′-azpy regions in their corresponding curvedness representations (SI FigureS20b,e).

