# Supplementary material for: Structural Landscape of α-Acetamidocinnamic Acid Cocrystals with Bipyridine-Based Coformers: Influence of Crystal Packing on Their Thermal and Photophysical Properties
Source: Cryst Growth Des. 2024 Feb 9;24(4):1746–65. doi: 10.1021/acs.cgd.3c01374 (PMC10885007; doi:10.1021/acs.cgd.3c01374)
Supplement: Supplementary file 1 — cg3c01374_si_001.pdf [file cg3c01374_si_001.pdf]

Supporting Information

Structural landscape of α-acetamidocinnamic acid cocrystals with bipyridine based coformers: influence of crystal packing on their thermal and photophysical properties

Daniel Ejarque [a], Teresa Calvet [b], Mercè Font-Bardia [c], and Josefina Pons [a,]*

[a]Departament de Química, Universitat Autònoma de Barcelona, 08193-Bellaterra, Barcelona, Spain

[b]Departament de Mineralogia, Petrologia i Geologia Aplicada, Universitat de Barcelona, Martí i Franquès s/n, 08028 Barcelona, Spain

[c]Unitat de Difracció de Raig-X, Centres Científics i Tecnològics de la Universitat de Barcelona (CCiTUB), Universitat de Barcelona, Solé i Sabarís, 1-3, 08028 Barcelona, Spain

*Corresponding author E-mail: josefina.pons@uab.es

PXRD patterns

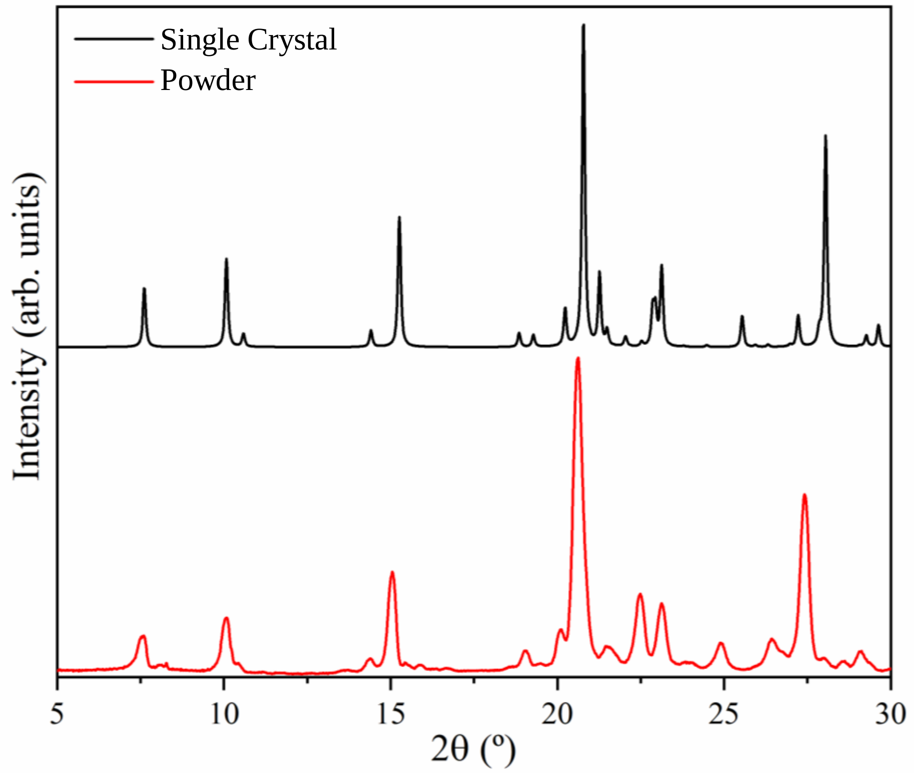

Figure S1. XRD patterns from the single crystal collected data at 100 K and powder XRD pattern at 298 K of HACA.

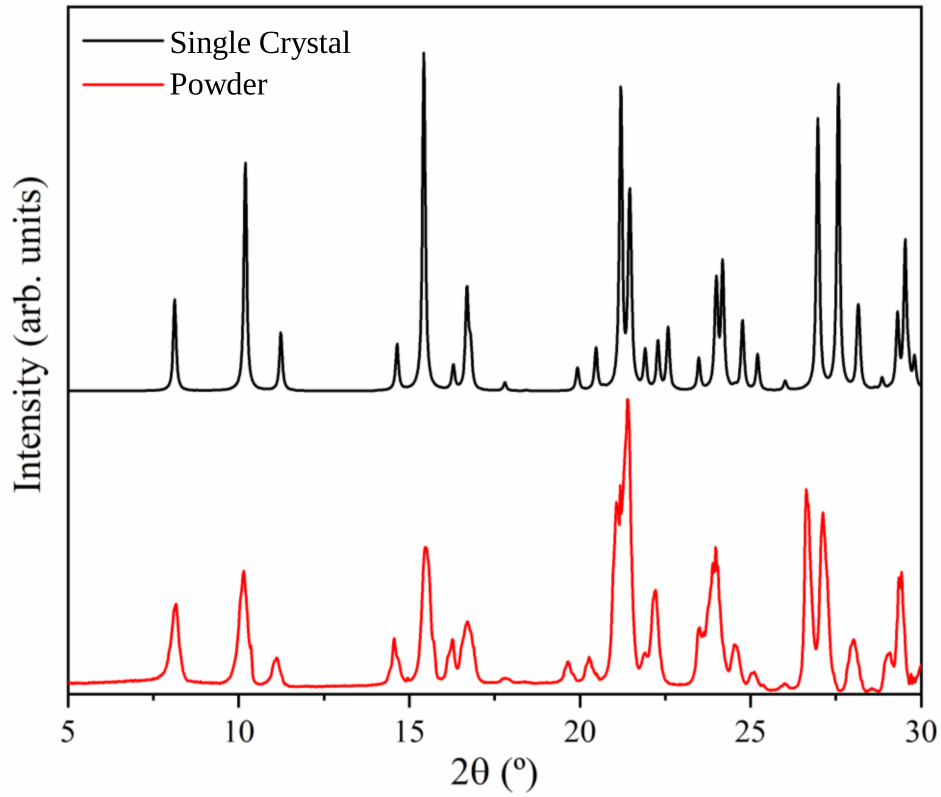

Figure S2. XRD patterns from the single crystal collected data at 100 K and powder XRD pattern at 298 K of HACA·$2H_2O$.

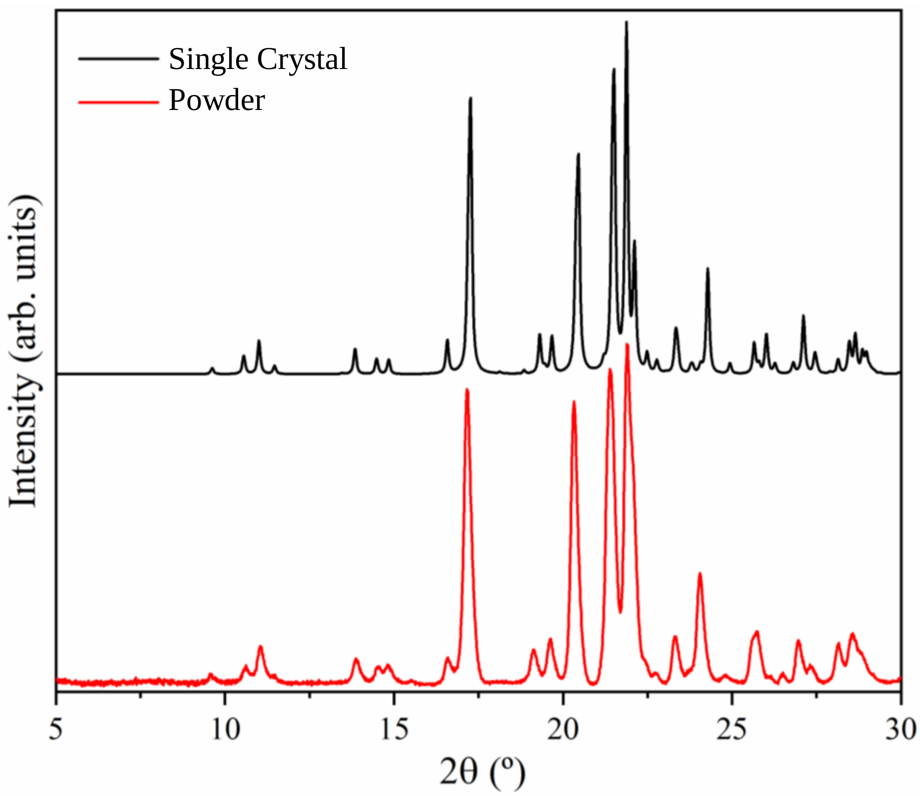

Figure S3. XRD patterns from the single crystal collected data at 100 K and powder XRD pattern at 298 K of cocrystal $(HACA)_2$(1,2-bpe) (**1**).

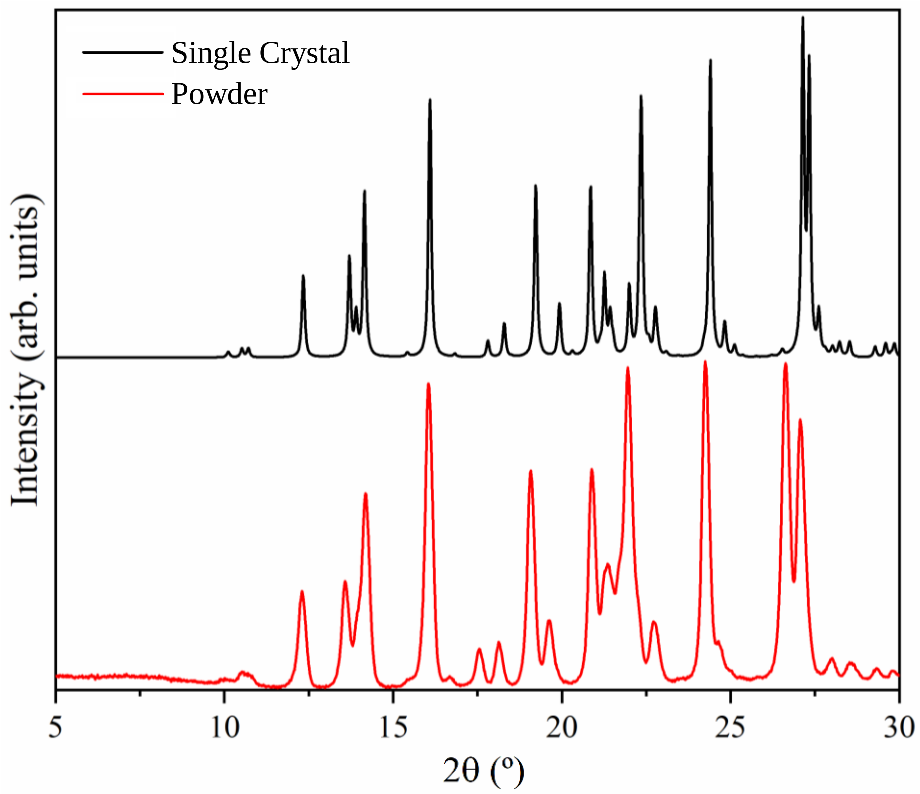

Figure S4. XRD patterns from the single crystal collected data at 100 K and powder XRD pattern at 298 K of cocrystal $(HACA)_2$(4,4'-azpy) (**2**).

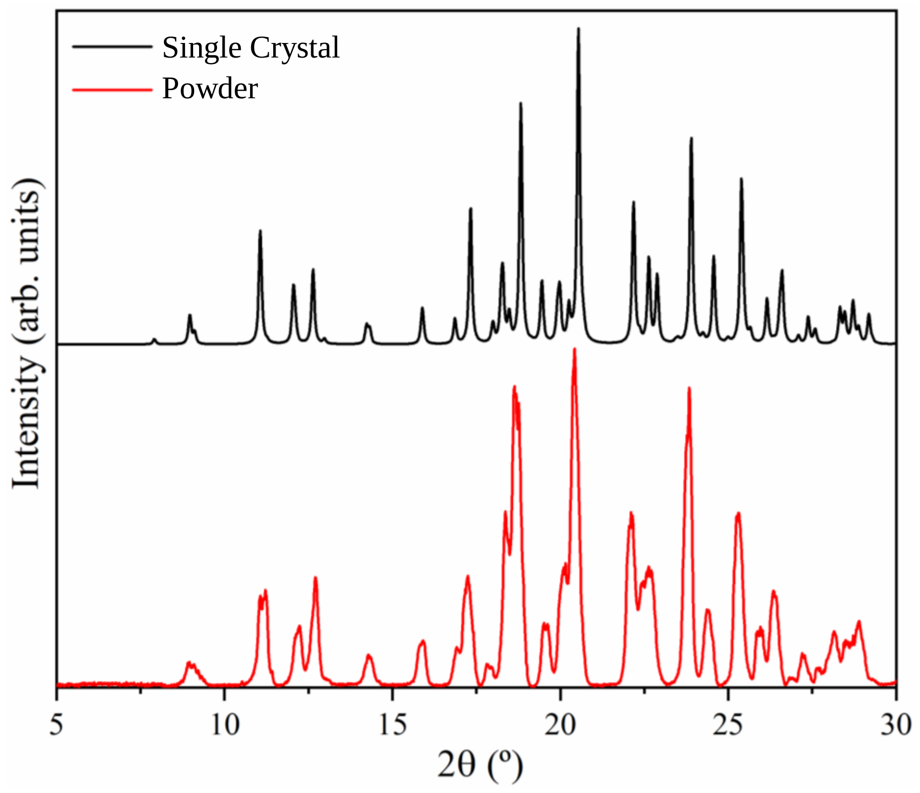

Figure S5. XRD patterns from the single crystal collected data at 100 K and powder XRD pattern at 298 K of cocrystal $(HACA)_2(4,4'\text{-bipy})_3$ (**3**).

FTIR-ATR, ^{1}H, ^{13}C{^{1}H} and DEPT-135 NMR spectroscopies

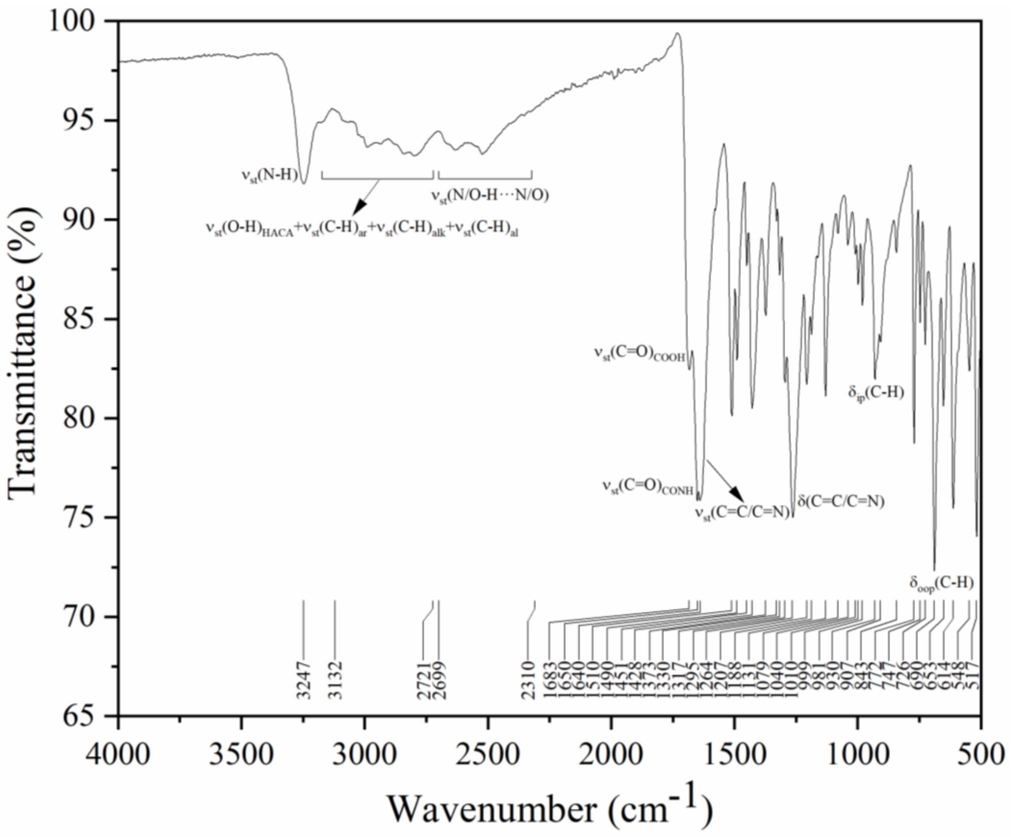

Figure S6. FTIR-ATR spectrum of HACA.

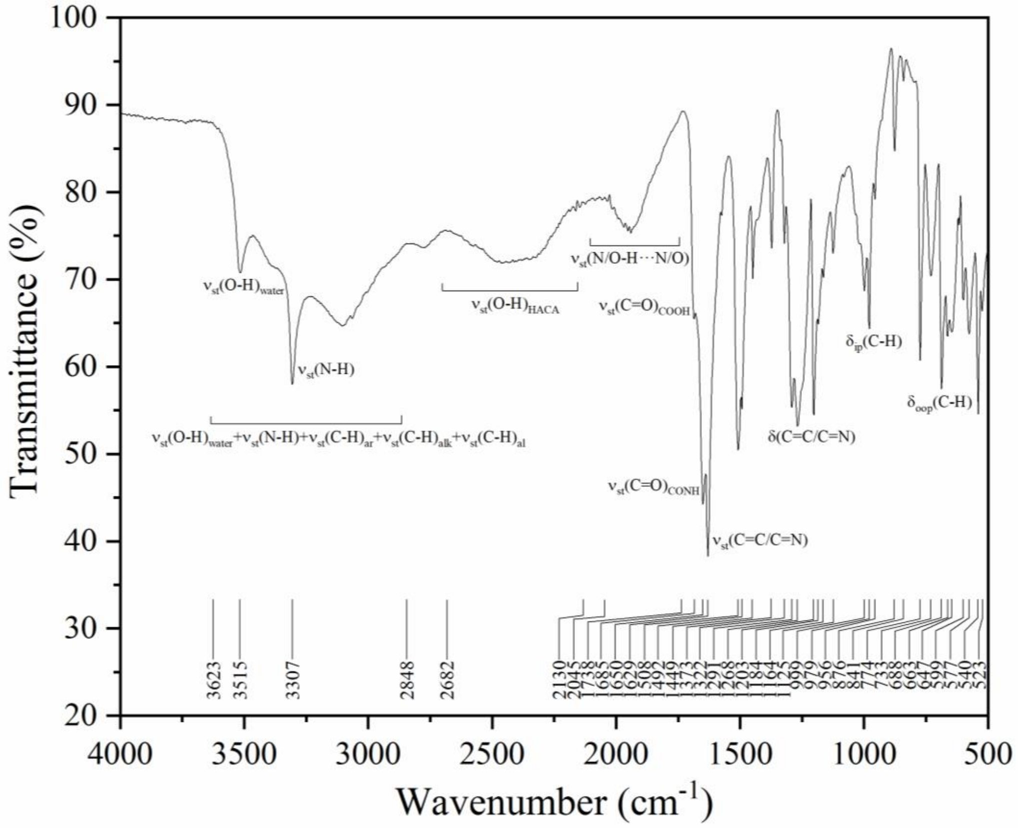

Figure S7. FTIR-ATR spectrum of HACA·$2H_2O$.

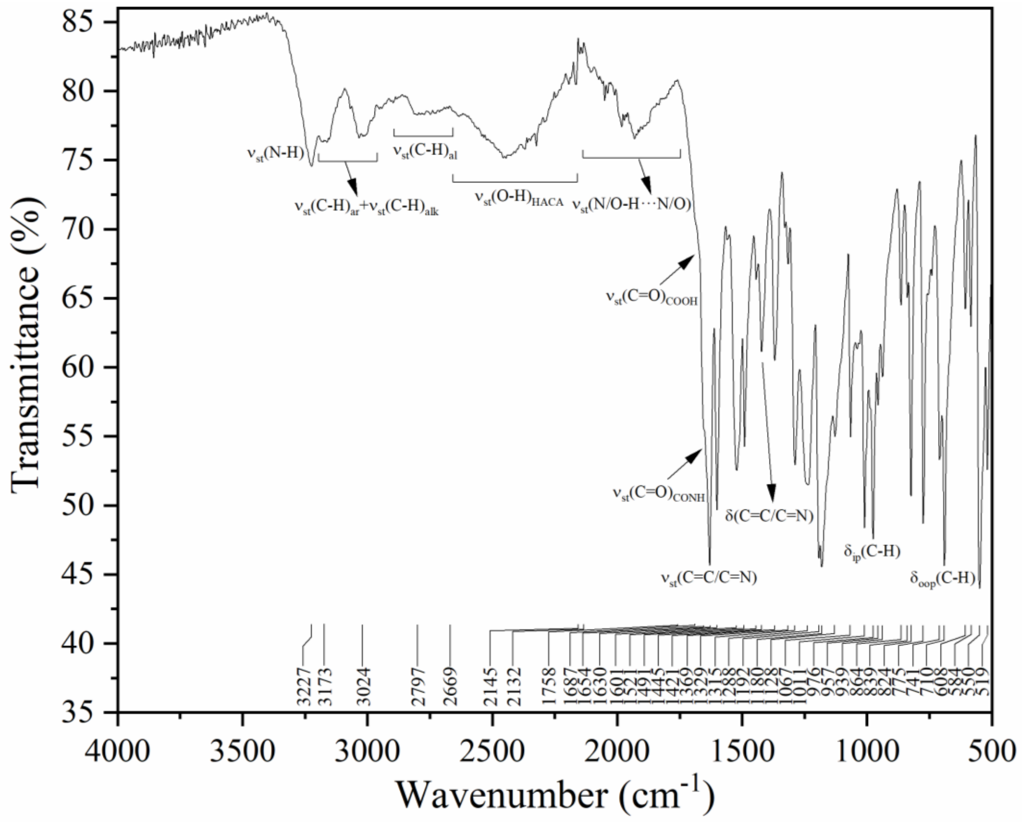

Figure S8. FTIR-ATR spectrum of cocrystal $(HACA)_2$(1,2-bpe) (**1**).

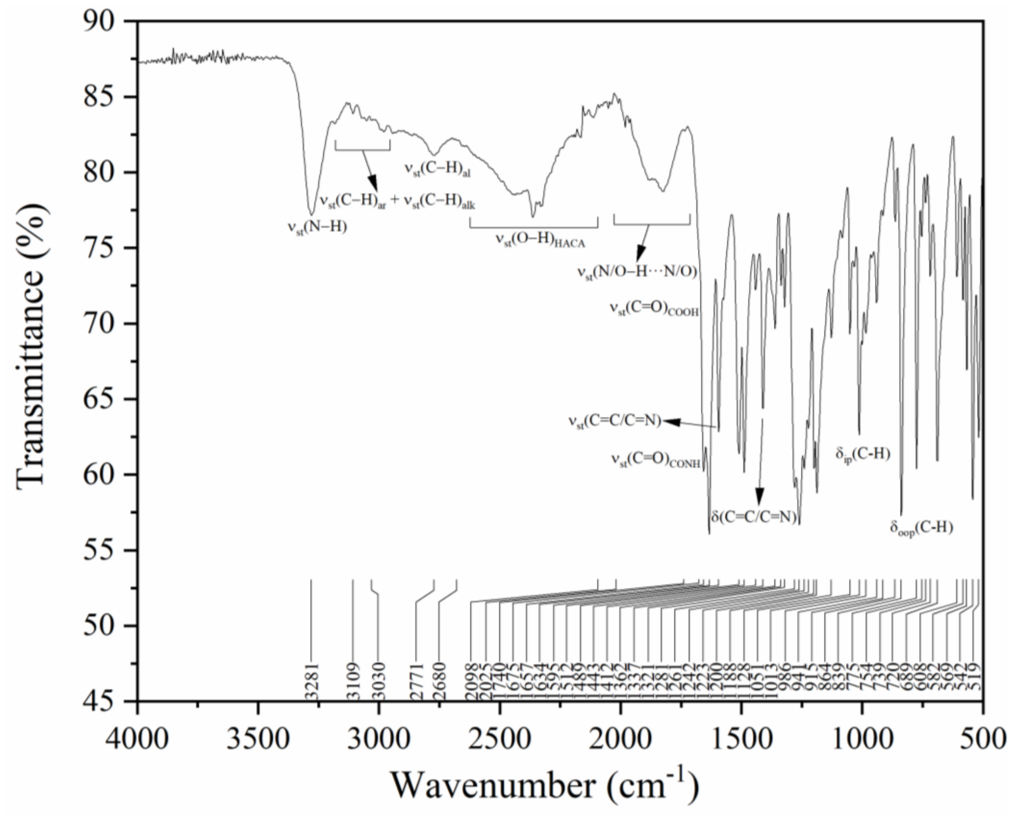

Figure S9. FTIR-ATR spectrum of cocrystal $(HACA)_2$(4,4'-azpy) (**2**).

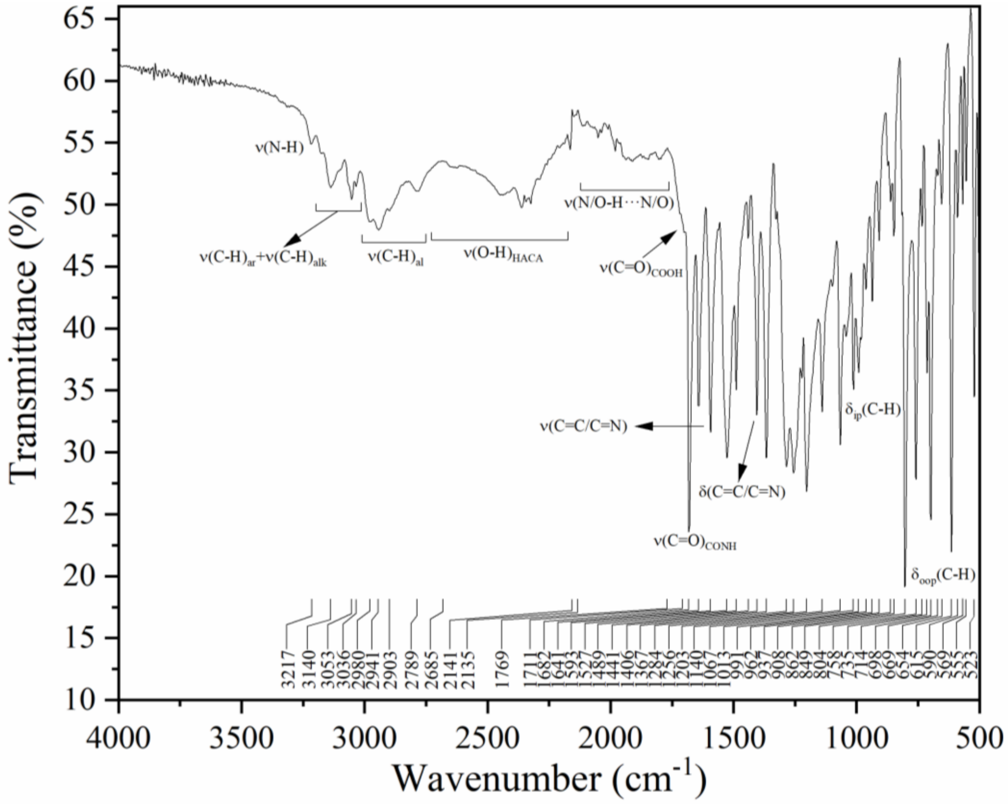

Figure S10. FTIR-ATR spectrum of cocrystal $(HACA)_2$(4,4'-bipy)$_3$ (**3**).

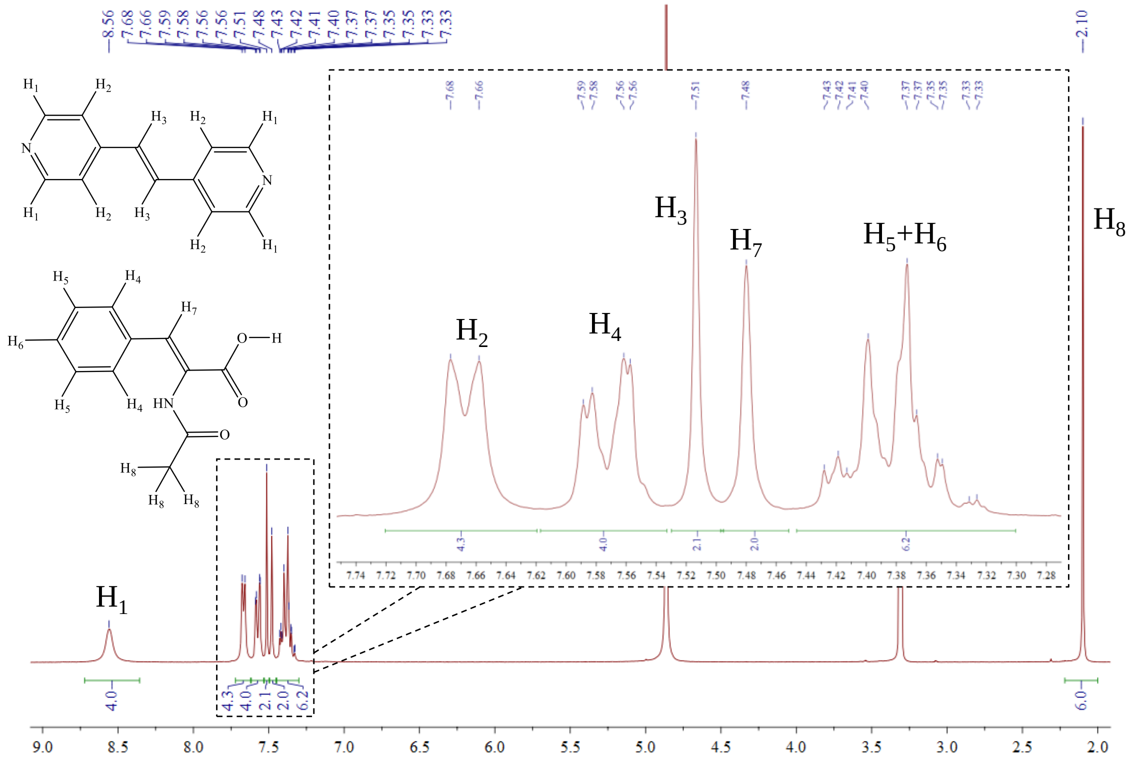

Figure S11. ^{1}H NMR spectrum of cocrystal $(HACA)_2$(1,2-bpe) (**1**) in CD_3OD.

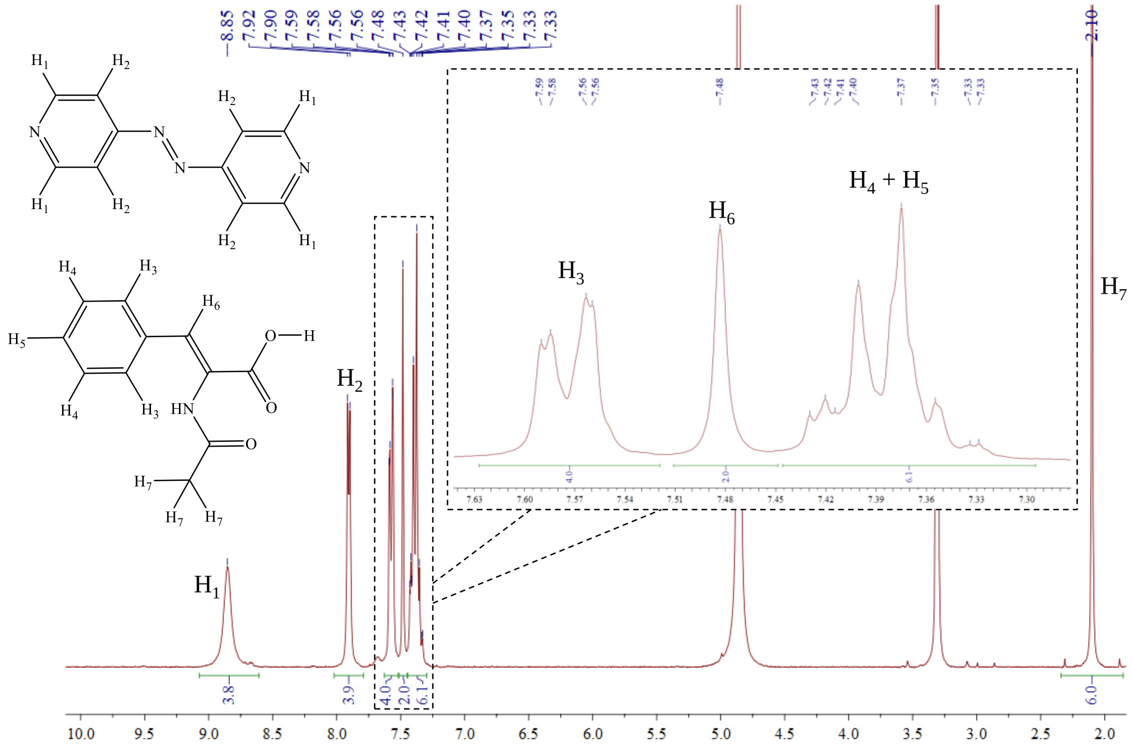

Figure S12. ^{1}H NMR spectrum of cocrystal $(HACA)_2$(4,4’-azpy) (**2**) in CD_3OD.

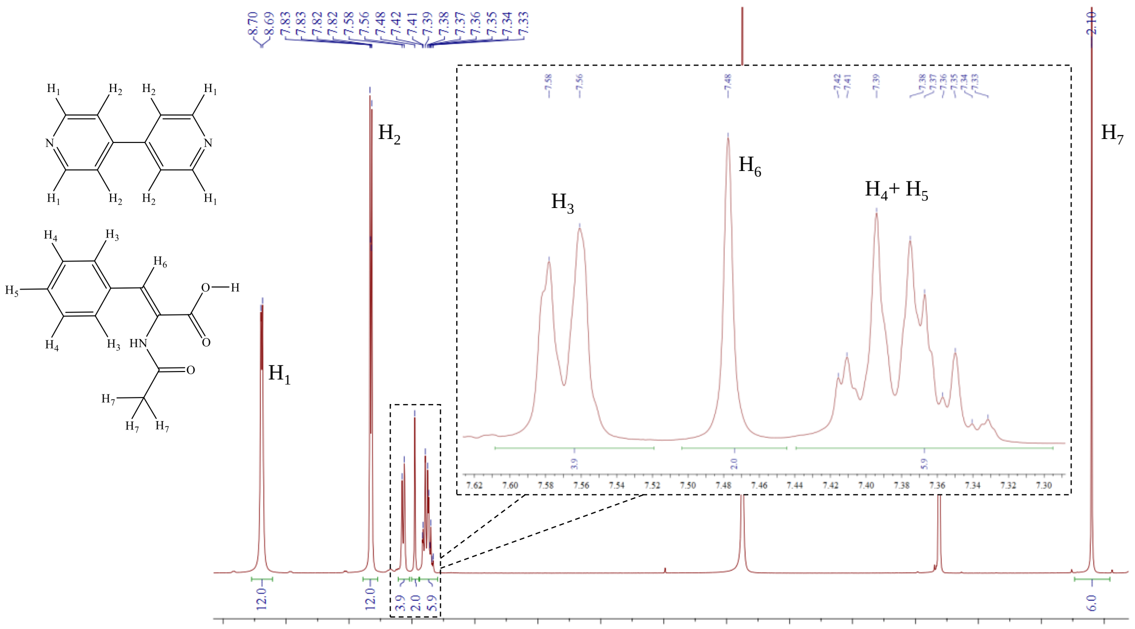

Figure S13. ^{1}H NMR spectrum of cocrystal (HACA)$_2$(4,4'-bipy)$_3$ (**3**) in CD_3OD.

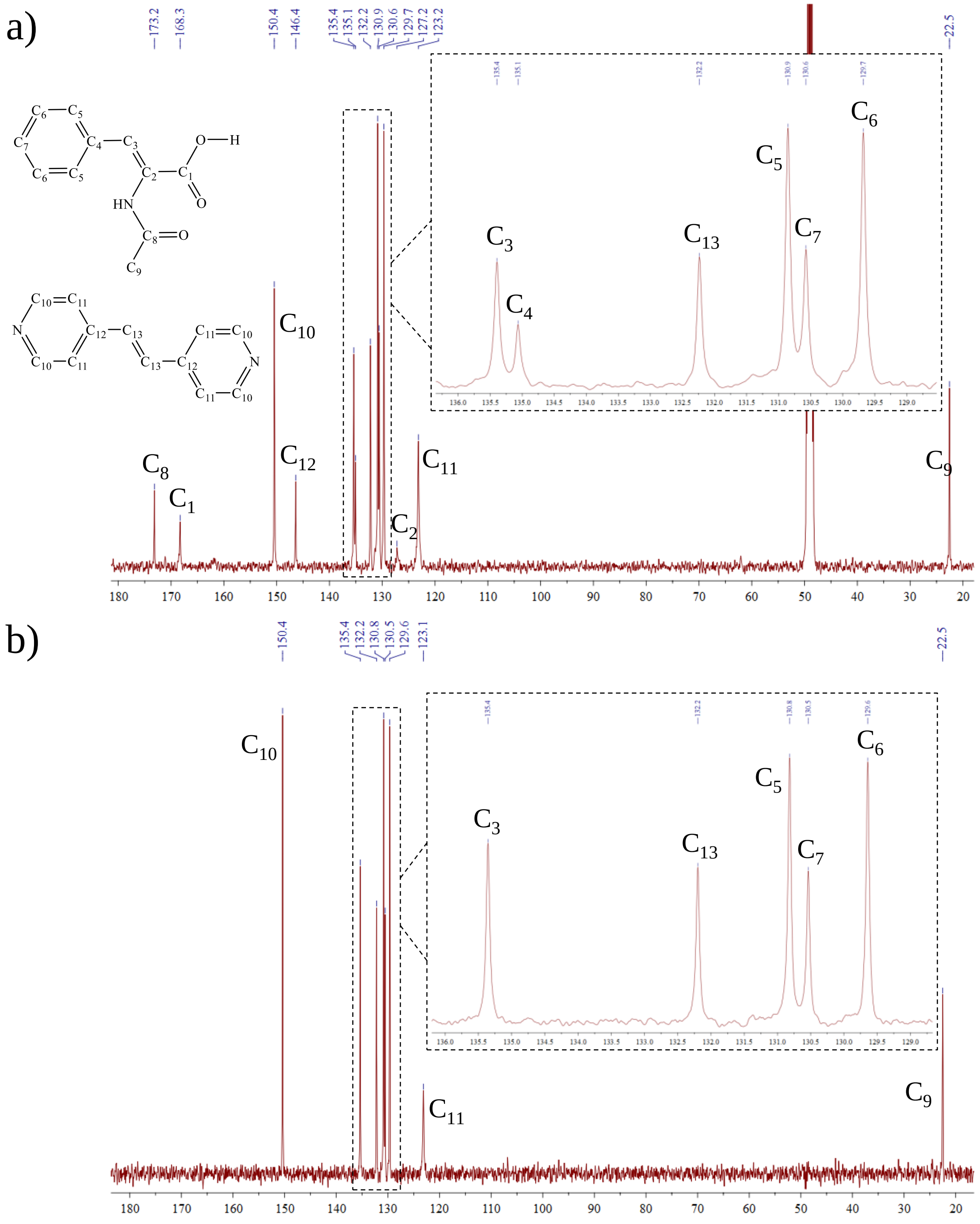

Figure S14. (a) $^{13}C\{^{1}H\}$ and (b) DEPT-135 NMR spectra of cocrystal $(HACA)_2$(1,2-bpe) (**1**) in CD_3OD.

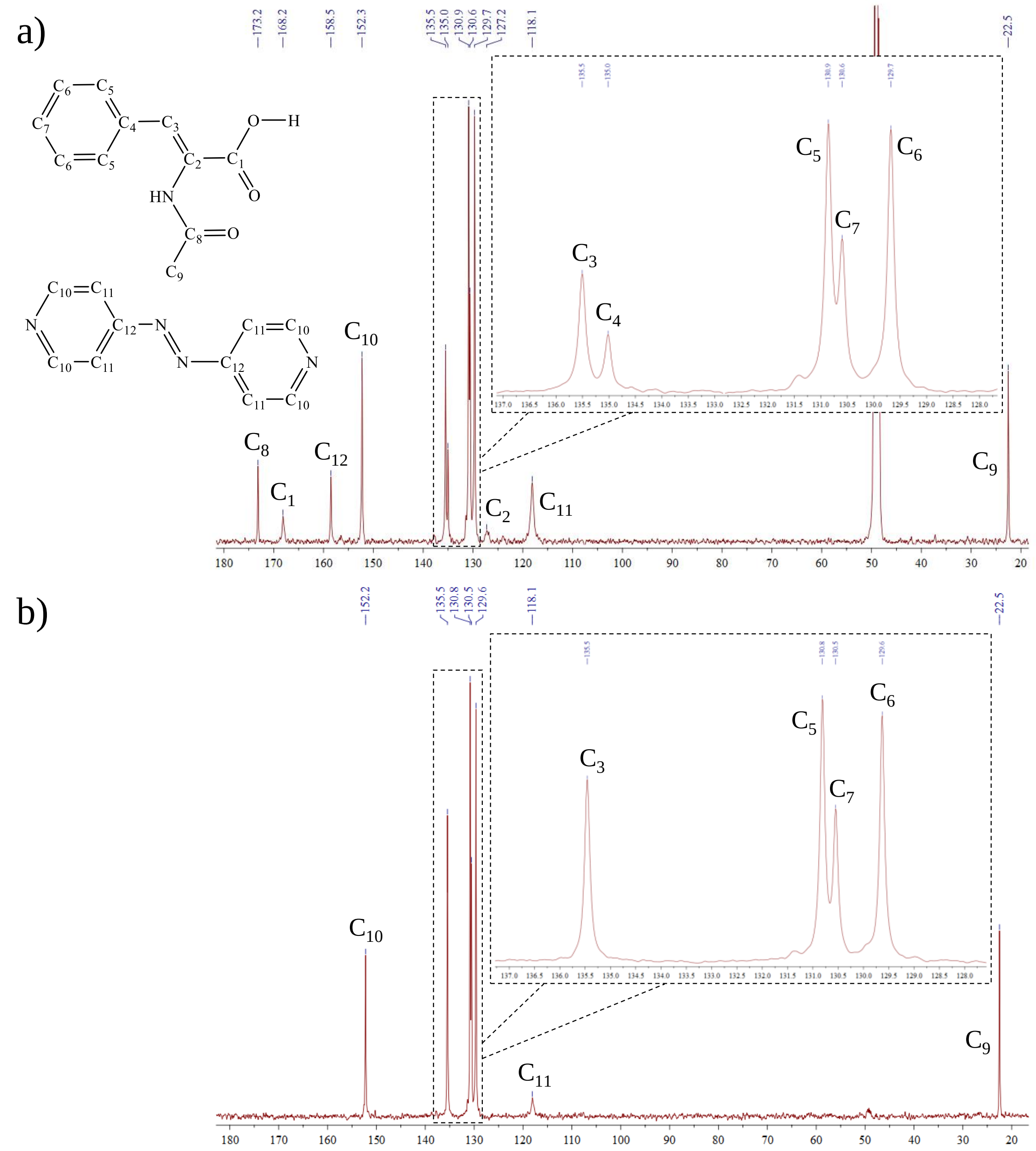

Figure S15. (a) $^{13}C\{^{1}H\}$ and (b) DEPT-135 NMR spectra of cocrystal $(HACA)_2(4,4'$-azpy) (**2**) in CD_3OD.

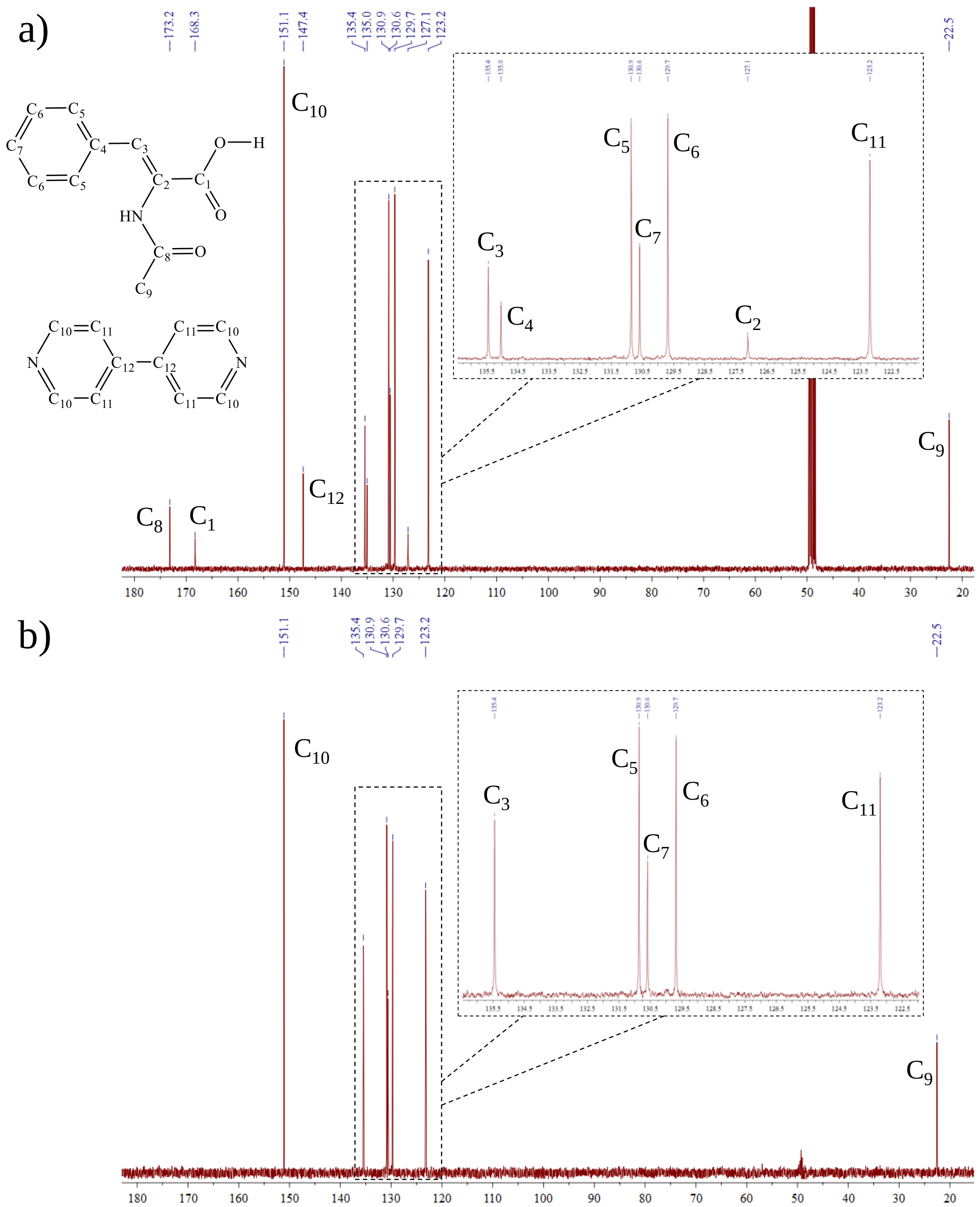

Figure S16. (a) $^{13}C\{^{1}H\}$ and (b) DEPT-135 NMR spectra of cocrystal $(HACA)_2(4,4'\text{-bipy})_3$ (**3**) in CD_3OD.

Structural descriptions and Hirshfeld Surface Analysis

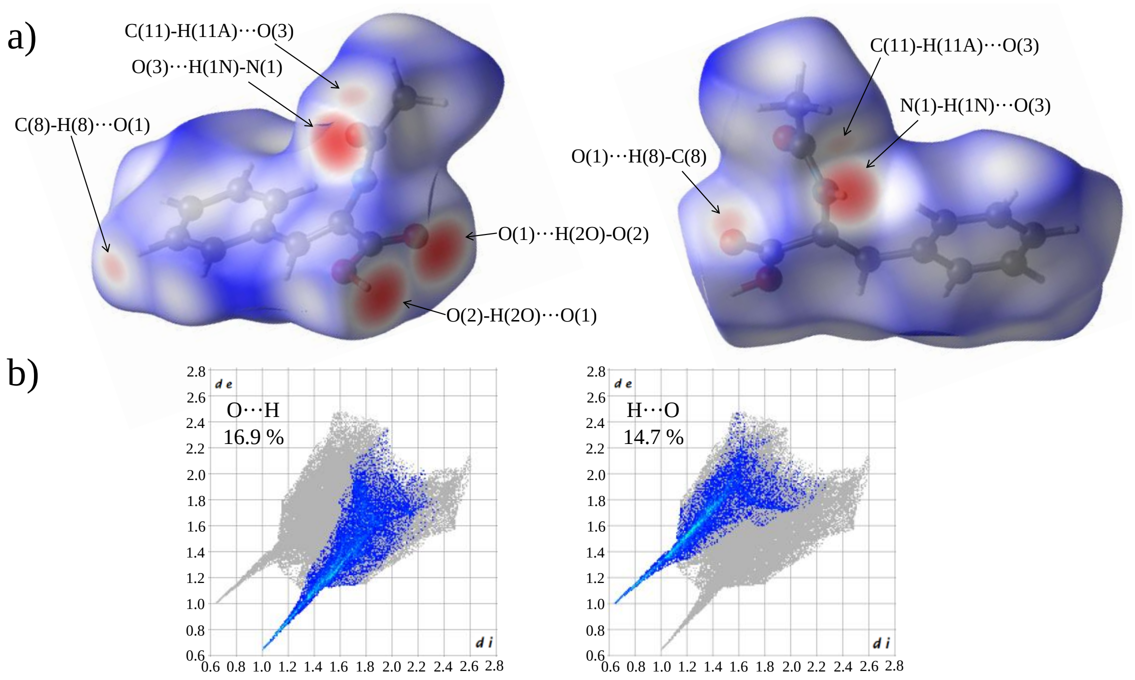

Figure S17. (a) Hirshfeld surfaces of HACA crystal structure mapped with d_{norm} representation. (b) 2D fingerprint plots of HACA crystal structure.

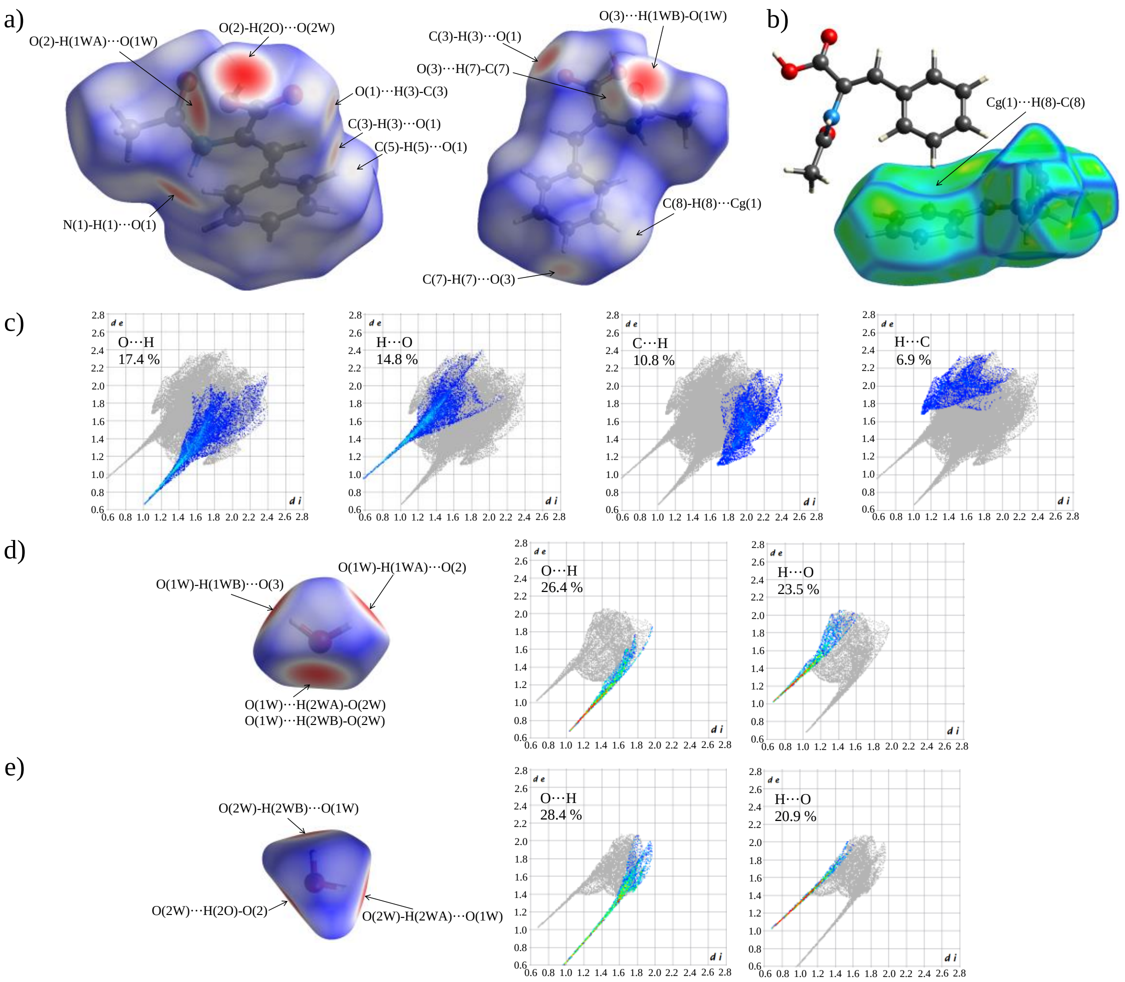

Figure S18. (a) Hirshfeld surfaces of HACA dihydrate crystal structure mapped with (a) d_{norm} and (b) curvedness representations. (c) 2D fingerprint plots of HACA single crystal. (d and e) Hirshfeld surfaces mapped with d_{norm} representation and 2D fingerprint plots of the water molecules of HACA dihydrate structure.

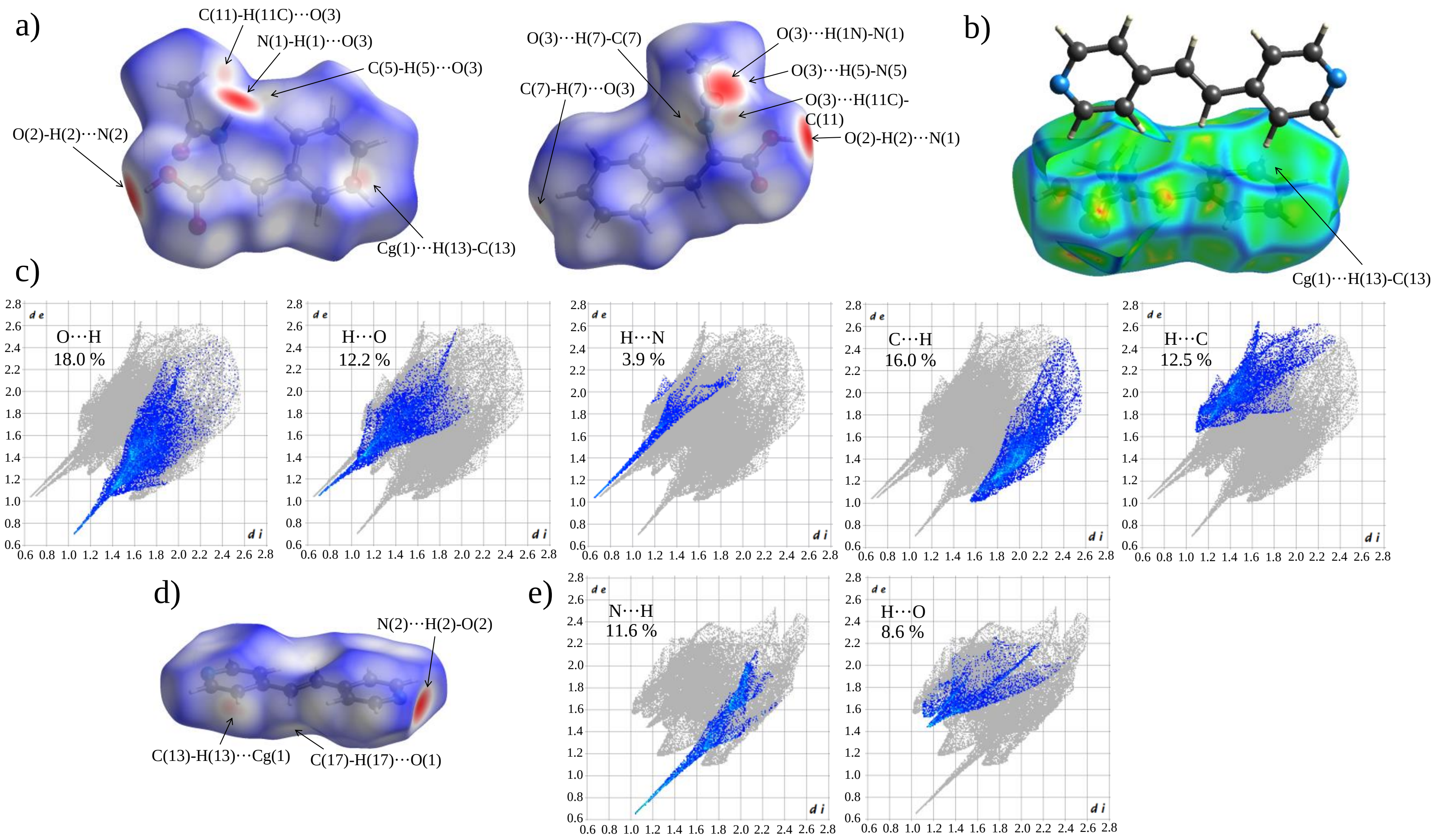

Figure S19. Hirshfeld surfaces of HACA in cocrystal **1** mapped with (a) d_{norm} and (b) curvedness representations. (c) 2D fingerprint plots of HACA in cocrystal **1**. (d) Hirshfeld surface of 1,2-bpe in cocrystal **1** mapped with d_{norm} representation. (e) 2D fingerprint plots of 1,2-bpe in cocrystal **1**.

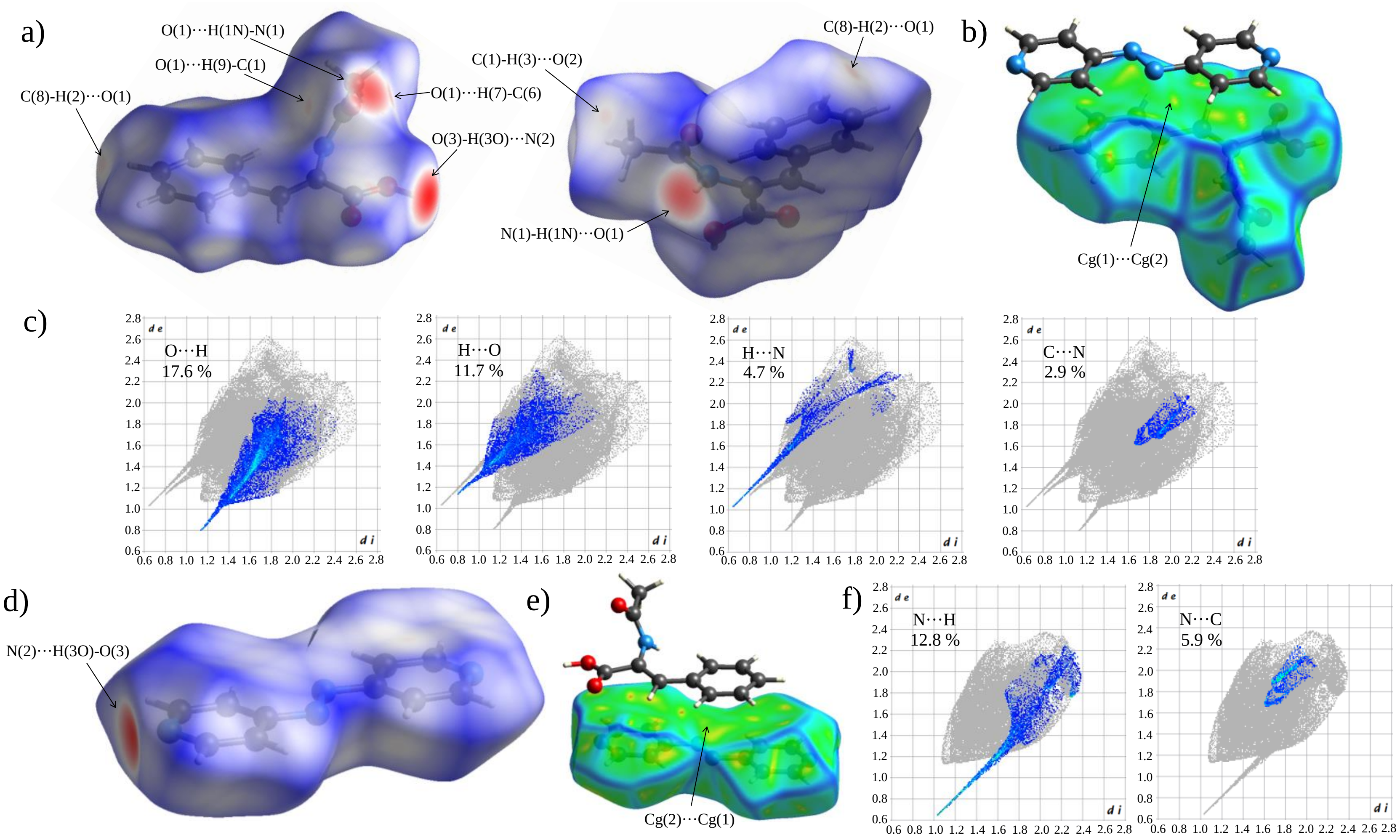

Figure S20. Hirshfeld surfaces of HACA in cocrystal **2** mapped with (a) d_{norm} and (b) curvedness representations. (c) 2D fingerprint plots of HACA in cocrystal **2**. Hirshfeld surface of 4,4'-azpy in cocrystal **2** mapped with (d) d_{norm} and (e) curvedness representations. (f) 2D fingerprint plots of 4,4'-azpy in cocrystal **2**.

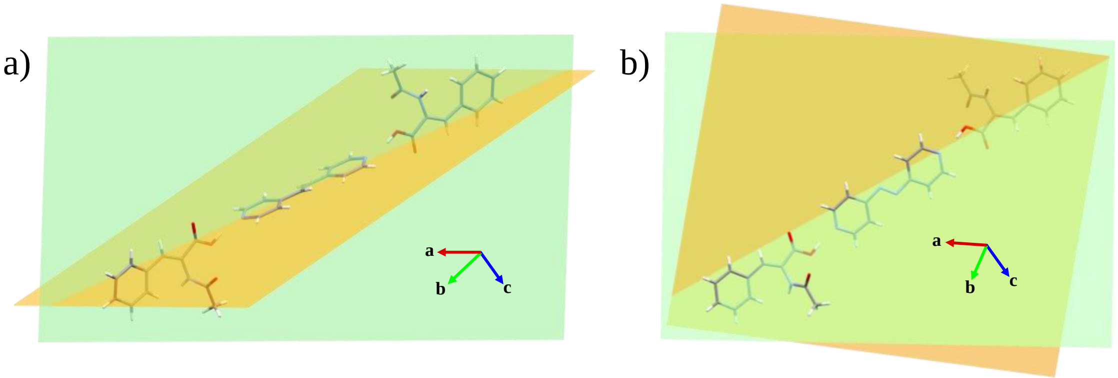

Figure S21. Representation of the angles between the HACA (green plane) and the dPy molecules (orange plane) in cocrystals (a) **1** (84.83º) and (b) **2** (22.11º).

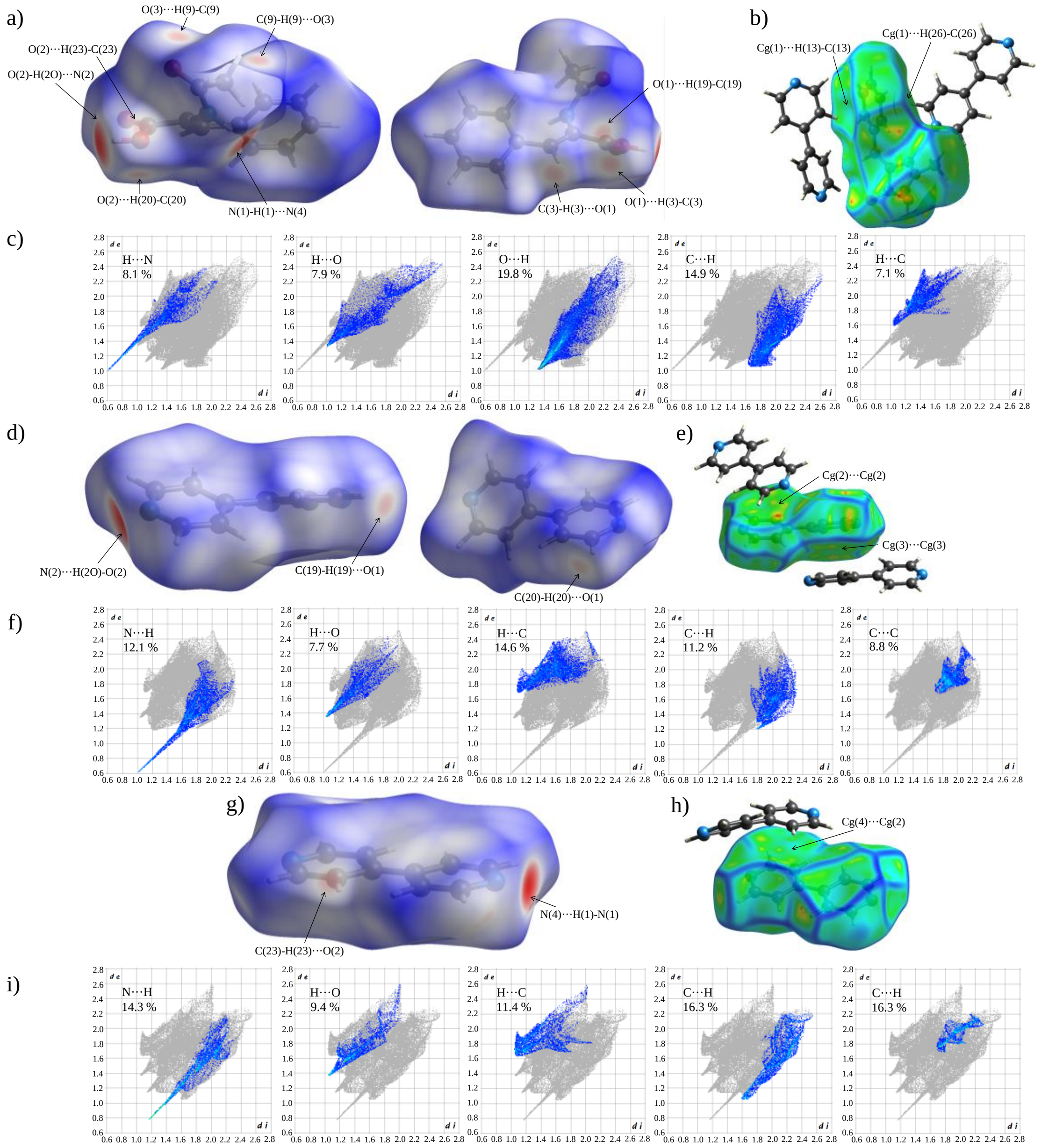

Figure S22. Hirshfeld surfaces of HACA in cocrystal **3** mapped with (a) d_{norm} and (b) curvedness representations. (c) 2D fingerprint plots of HACA in cocrystal **3**. Hirshfeld surface of 4,4'-bipy in cocrystal **3** mapped with (d and g) d_{norm} and (e and h) curvedness representations. (f and i) 2D fingerprint plots of 4,4'-bipy in cocrystal **3**.

Synthon competitivity between acid and amide groups in bipyridine based cocrystals

Table S1. CSD results of the cocrystal structures containing acid and amide groups with bipyridine-based coformers.

CSD code	Carboxylic Acid	Bipyridine-based coformer	Synthon outcome[a]	Ref.
Cocrystal **1**	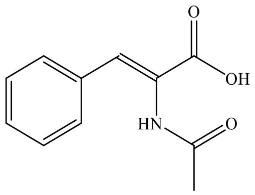	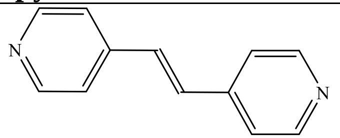 1,2-*bis*(4-pyridyl)ethylene	Types I and VI	This work
Cocrystal **2**		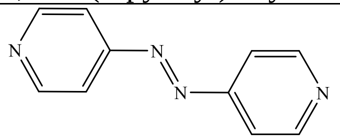 4,4’-azopyridine	Types I and VI	This work
Cocrystal **3**	α-acetamidocinnamic acid	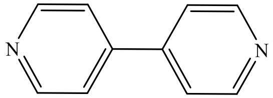 4,4’-bipyridine	Types I and VII	This work
CAZYUB	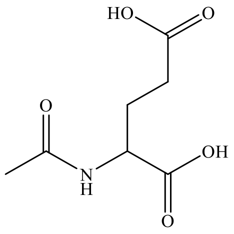	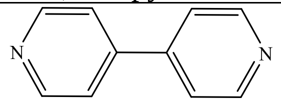 4,4’-bipyridine	Types I and VI	[1]
CEBBUK	N-Acetylglutamic acid	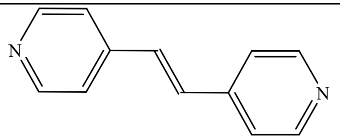 1,2-*bis*(4-pyridyl)ethylene	Types I and VI	[1]

[a] Synthon outcome types are specified in figure 7b of the manuscript.

Table S1. *Cont*.

CSD code	Carboxylic Acid	Bipyridine-based coformer	Synthon outcome[a]	Ref.
CEBBIY	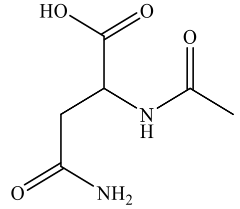 N-Acetylasparagine	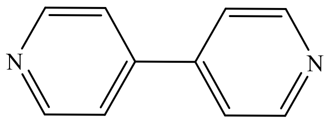 4,4'-bipyridine	Types I, III, IV, V and VI	[1]
CEBBOE	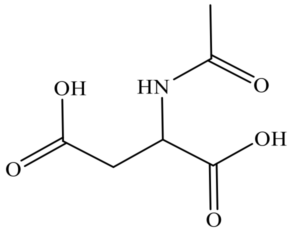 N-Acetylaspartic acid	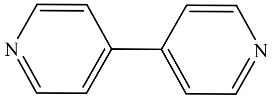 4,4'-bipyridine	Types I, VII, and VIII	[1]
CEBDAS		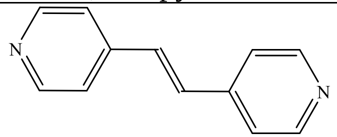 1,2-*bis*(4-pyridyl)ethylene	Types I and VII	[1]
CEBCIZ	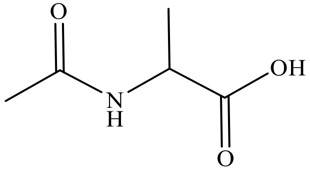 N-Acetylalanine	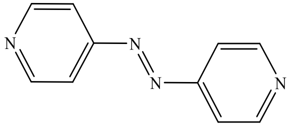 4,4'-azopyridine	Types I and IX	[1]
HEDPIT		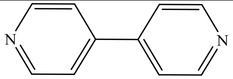 4,4'-bipyridine	Types I and IX	[2]
LEKQIF		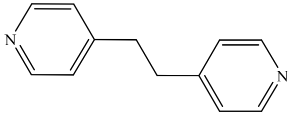 1,2-*bis*(4-pyridyl)ethane	Types I and IX	[3]

[a] Synthon outcome types are specified in figure 7b of the manuscript.

Table S1. *Cont.*

**CSD code**	**Carboxylic Acid**	**Bipyridine-based coformer**	**Synthon outcome[a]**	**Ref.**
JADBAU	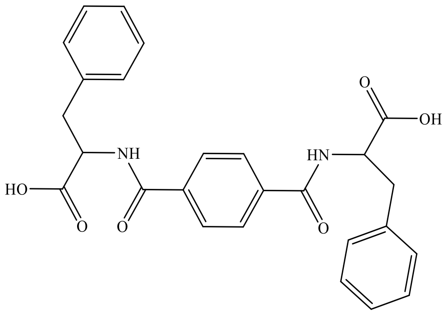 1,4-phenylenebis(carbonylimino))bis(3-phenylpropanoic acid)	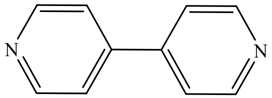 4,4'-bipyridine	Types I and IX	[4]
ROQYED	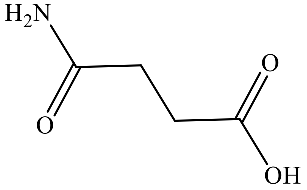 Succinamic acid	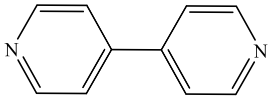 4,4'-bipyridine	Types I and II	[5]

[a] Synthon outcome types are specified in figure 7b of the manuscript.

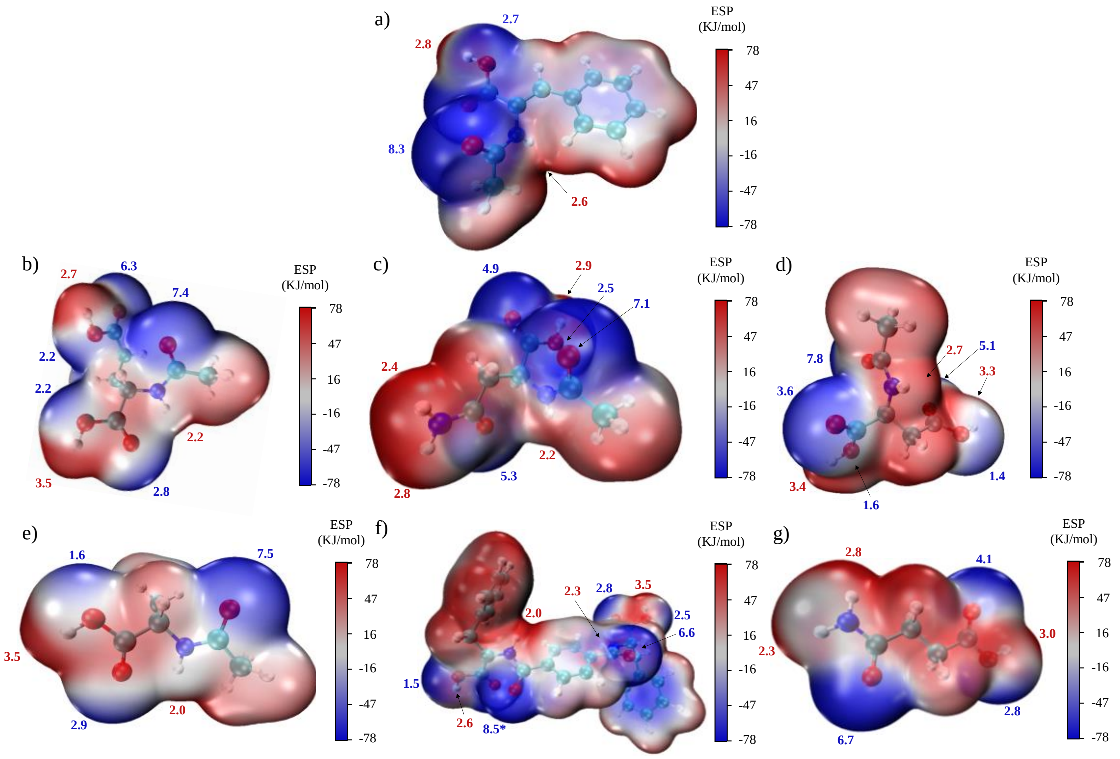

Figure S23. Main α and β values of the cocrystal formers containing and acid and an amide group within the same molecule found on the literature: (a) α-acetamidocinnamic acid, (b) N-acetylglutamic acid, (c) N-acetylasparagine, (d) N-acetylaspartic acid, (e) N-acetylalanine, (f) 1,4-phenylenebis(carbonylimino))bis(3-phenylpropanoic acid), and (g) succinamic acid.

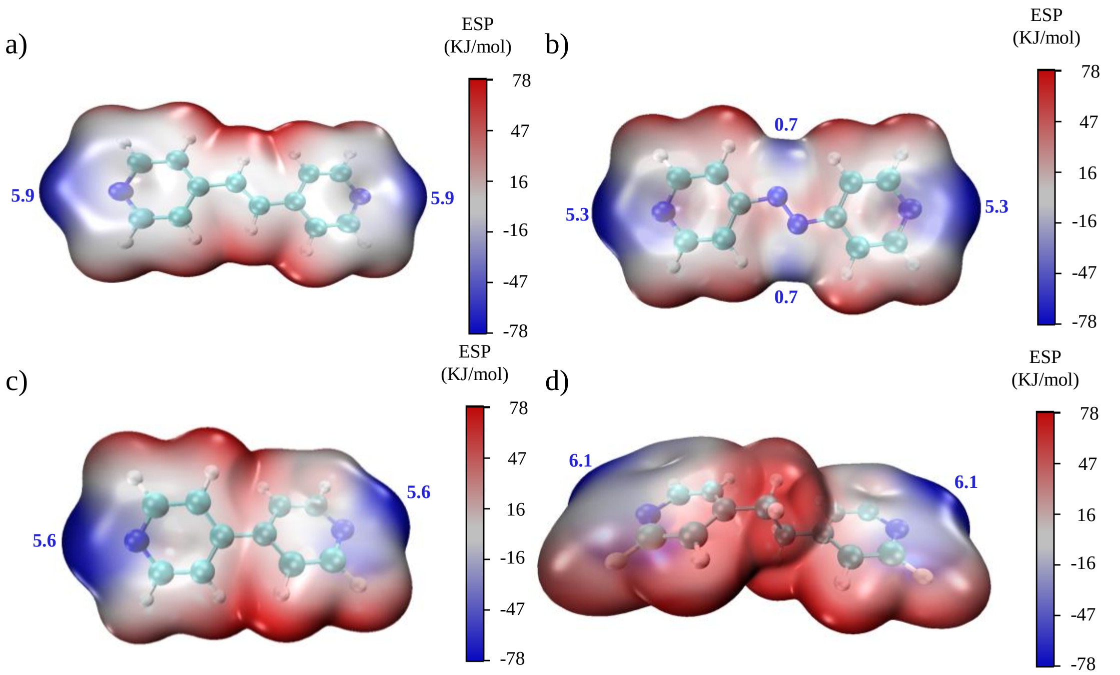

Figure S24. β values of the bipyridine coformers from the cocrystals containing and acid and an amide group within the same molecule found on the literature: (a) 1,2-*bis*(4-pyridyl)ethylene, (b) 4,4'-azopyridine, (c) 4,4'-bipyridine, and (d) 1,2-*bis*(4-pyridyl)ethane.

Thermal properties

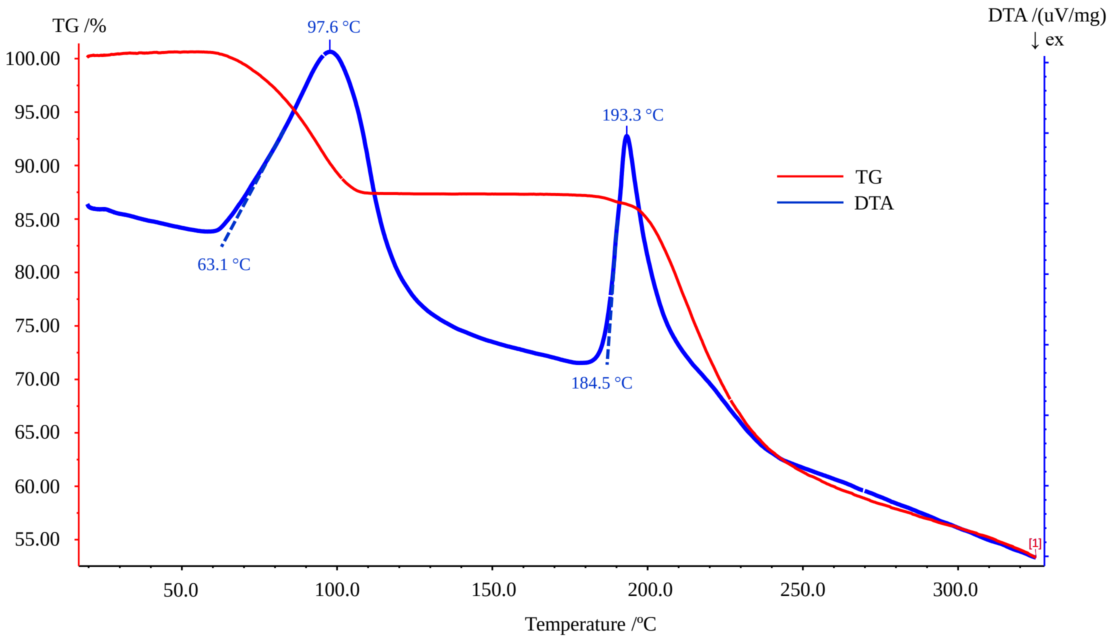

Figure S25. Simultaneous TG/DTA plots of HACA·$2H_2O$.

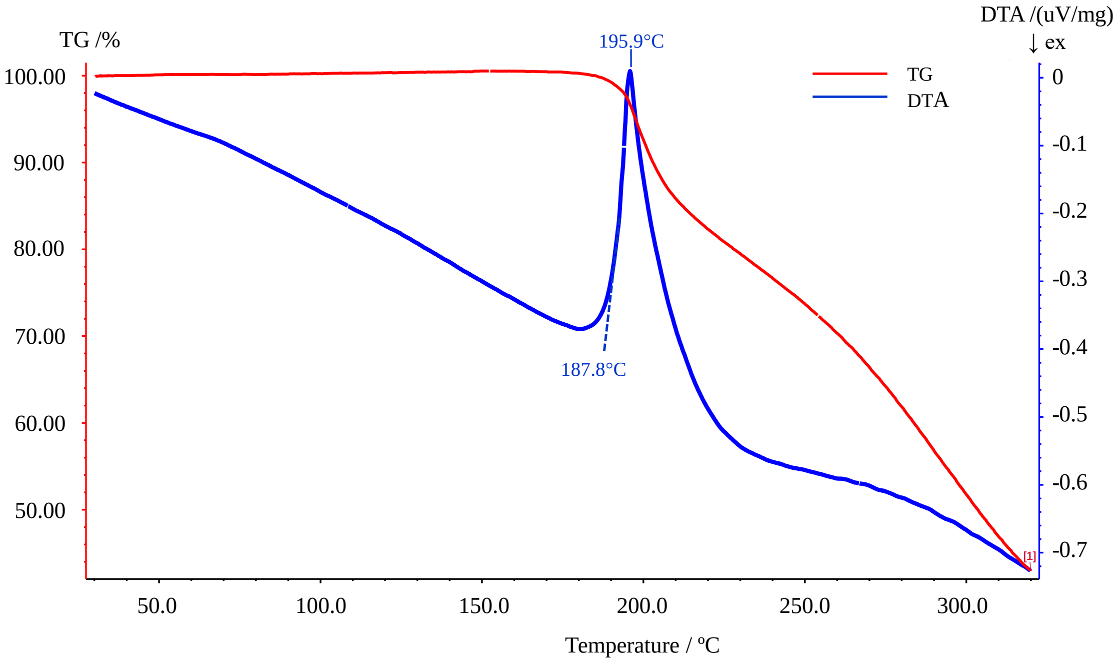

Figure S26. Simultaneous TG/DTA plots of cocrystal **1**.

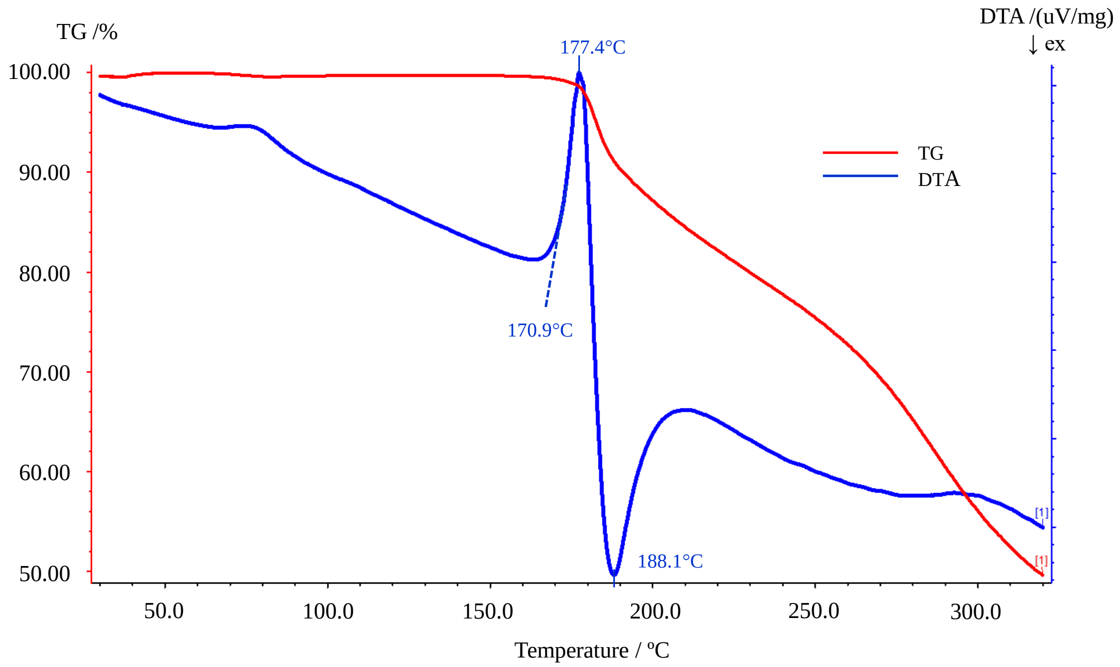

Figure S27. Simultaneous TG/DTA plots of cocrystal **2**.

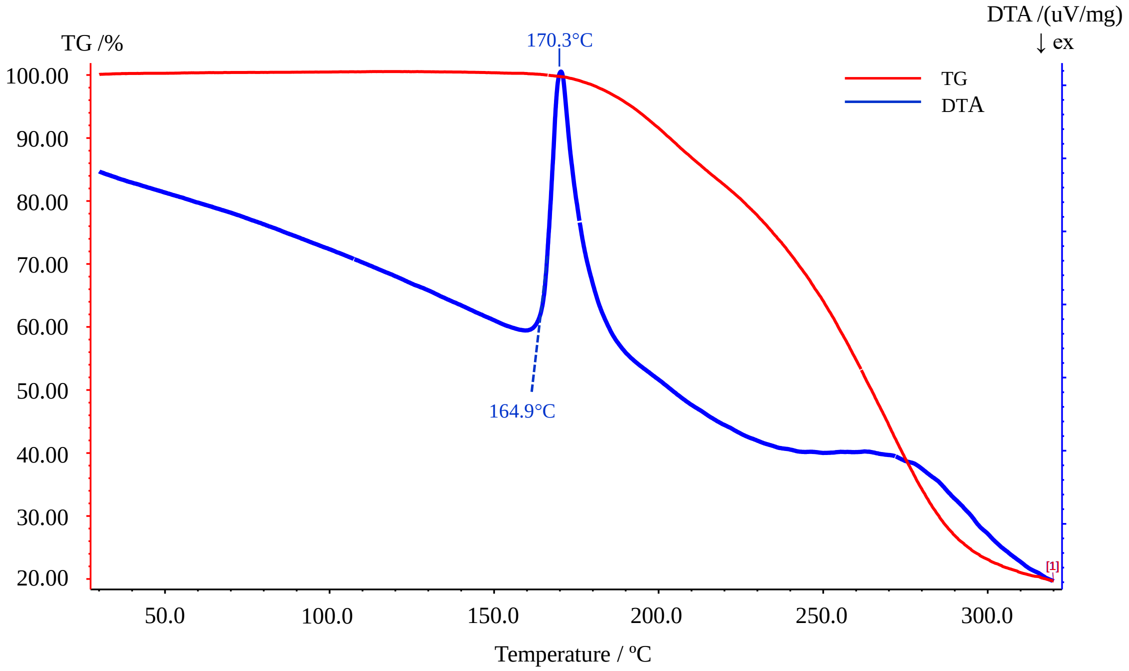

Figure S28. Simultaneous TG/DTA plots of cocrystal **3**.

Table S2. Melting point values of the utilized components and the resulting crystalline forms of this work.

Component	Melting Point (ºC)[a]
1,2-bpe	149-150
4,4’-azpy	106-107
4,4’-bipy	112-113
HACA	189-190
HACA·2H_2O	189-190
Cocrystal **1**	194-195
Cocrystal **2**	180-181
Cocrystal **3**	169-170

[a]All the melting points have been determined using the same apparatus detailed in the experimental section of the manuscript.

Table S3. Contribution and lattice energies of the crystal structures of HACA, HACA·2H_2O and cocrystals **1-3**. All the values have been obtained using CrystalExplorer 17.5 from the corresponding .cif files.[a]

Structure	Molecule	E_{ele}[b]	E_{pol}[c]	E_{dis}[d]	E_{rep}[e]	E_{tot}[f]	E_{latt}[g]
HACA		-287.8	-58.5	-217.1	229.8	-333.6	-166.8
HACA·2H_2O	HACA	-312.7	-59.6	-224.1	249.4	-347.0	-234.8
	H_2O[h]	-197.4	-32.3	-27.4	135.5	-121.6	
	H_2O[h]	-200.7	-31.0	-30.9	138.9	-123.7	
Cocrystal **1**	HACA	-249.7	-48.4	-215.1	221.1	-292.1	-275.7
	1,2-bpe	-215.6	-37.3	-203.8	197.4	-259.3	
Cocrystal **2**	HACA	-242.7	-46.8	-231.7	204.6	-316.6	-288.1
	4,4’-azpy	-212.7	-38.5	-218.8	210.4	-259.6	
Cocrystal **3**	HACA	-240.4	-46.9	-225.2	212.5	-300.3	-258.1
	4,4’-bipy[h]	-148.2	-25.3	-190.9	142.8	-221.6	
	4,4’-bipy[h]	-140.2	-25.0	-191.6	146.7	-210.1	

[a]All the energies are given in KJ/mol; [b]E_{ele} = electrostatic energy; [c]E_{pol} = electrostatic energy; [c]E_{dis} = dispersion energy; [d]E_{rep} = repulsion energy; [e]E_{tot} = total energy; [f]E_{latt} = lattice energy. [h]Rows with repeated components in each specific compound stands for different types of this molecule within the crystal structure.

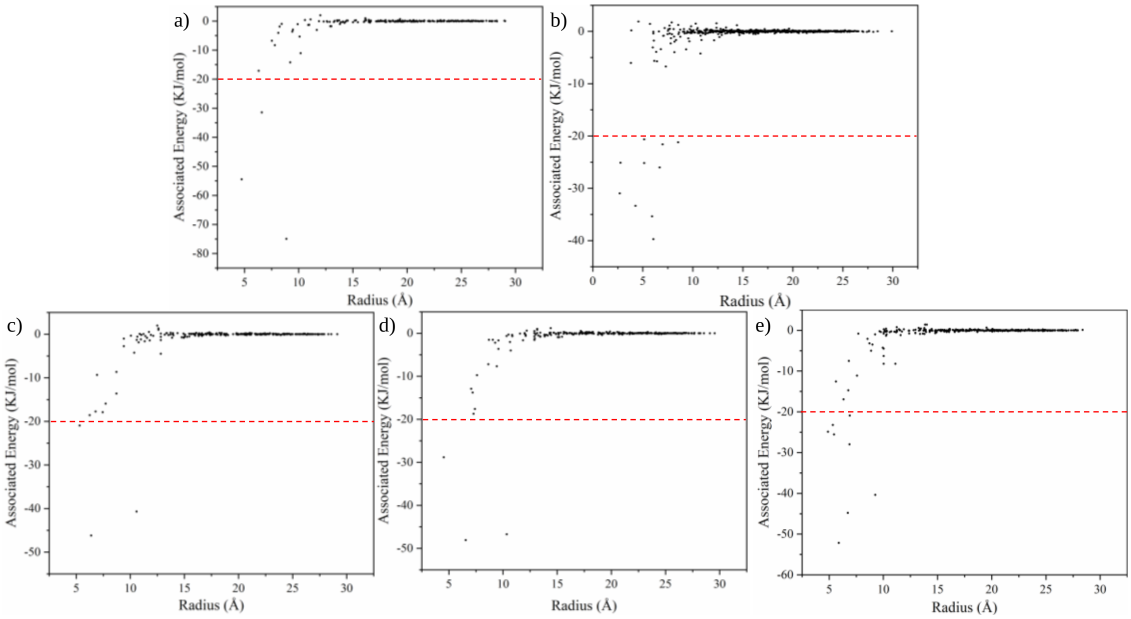

Figure S29. Relationship between the molecular pair radius and the associated interaction energies with the assigned energy threshold marked for (a) HACA, (b) HACA·$2H_2O$, and cocrystals (c) **1**, (d) **2**, and (e) **3**.

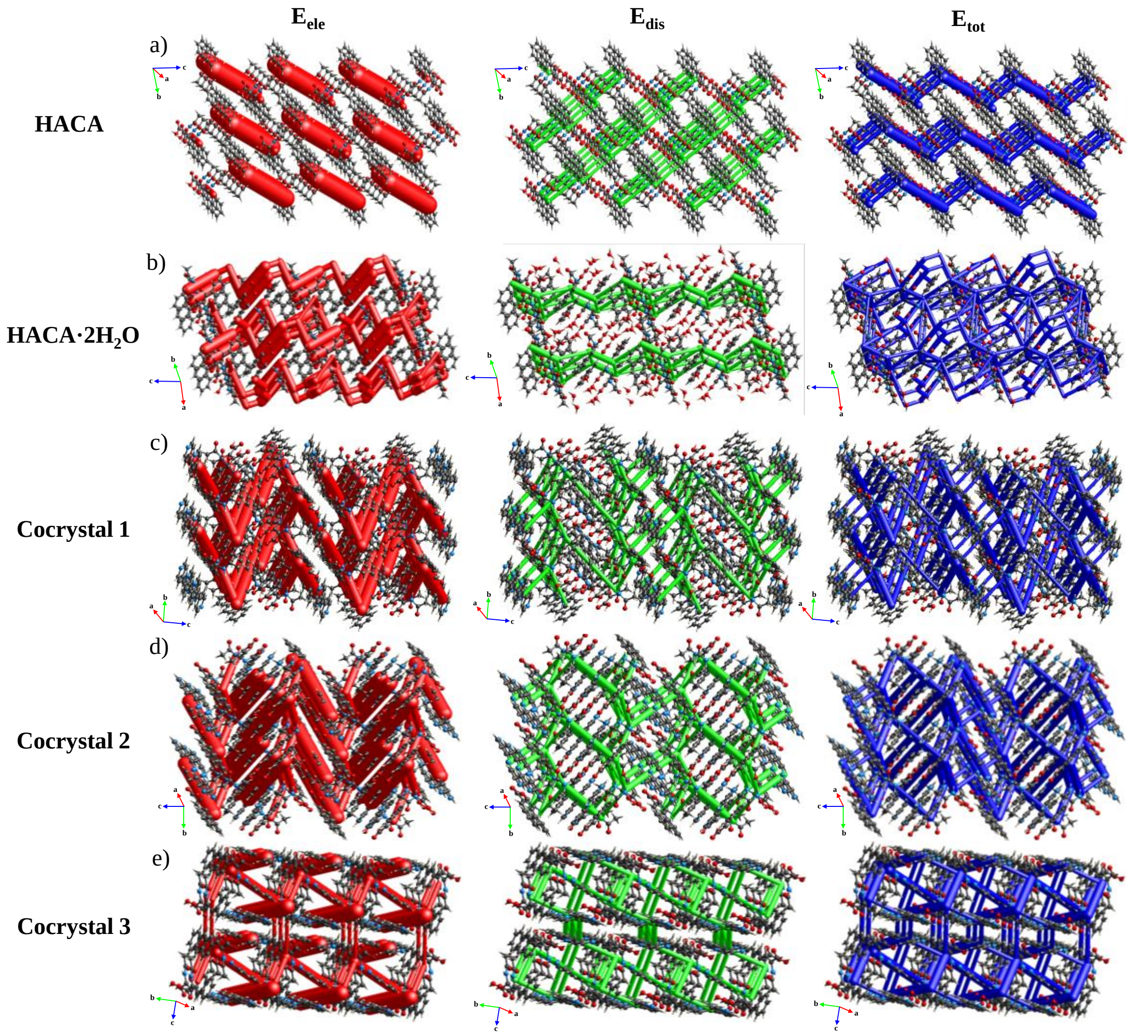

Figure S30. Energy frameworks (E_{ele}, E_{dis}, E_{tot}) for (a) HACA, (b) HACA·2H_2O, and cocrystals (c) **1**, (d) **2**, and (e) **3**. All the diagrams use the same energy cylinder scale factor of 120 and an energy cut-off of -20 KJ/mol within a 3×2×3 (HACA), 2×2×2 (HACA·2H_2O and cocrystals **1** and **2**), 2×3×2 (cocrystal **3**).

Photophysical properties

Table S4. Detailed parameters extracted from the photophysical properties of HACA, HACA·$2H_2O$, and cocrystals **1-3**.

Compound	$\lambda_{max-Abs}$ (nm)	λ_{exc} (nm)	Main λ_{max-em} (nm)	Stokes shift (cm^{-1})
HACA	246, 294, 390	250	420	16190
HACA·$2H_2O$	252, 324, 400			
Cocrystal **1**	252, 322			
Cocrystal **2**	259, 314, 390, 492			
Cocrystal **3**	286			

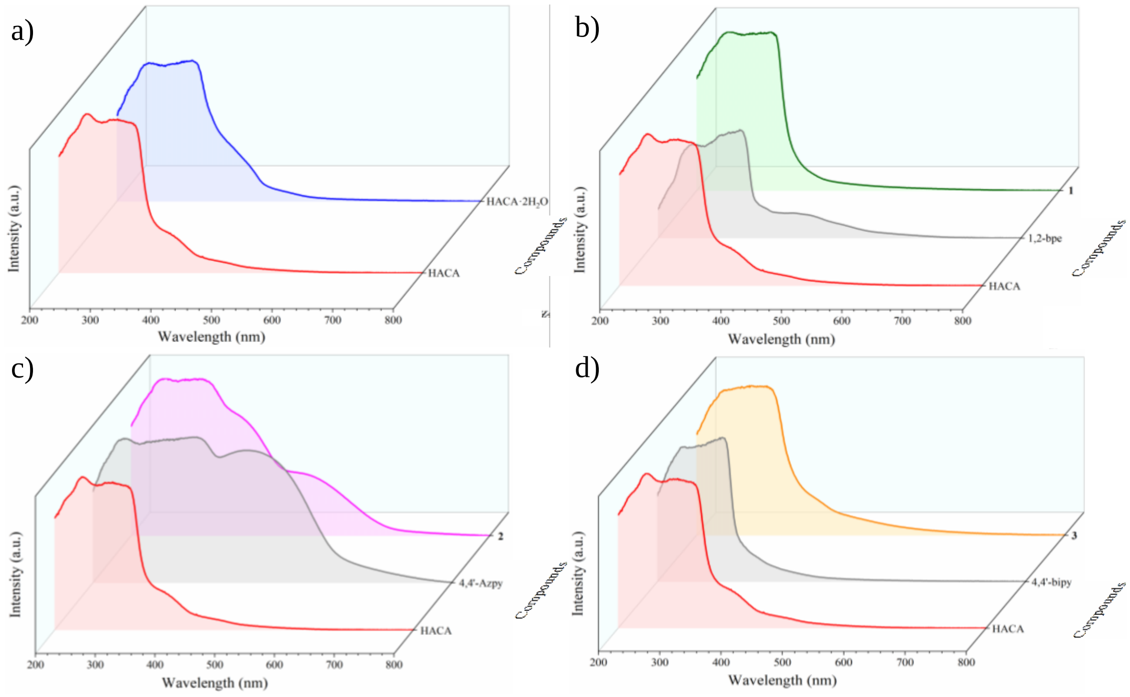

Figure S31. Comparison of the solid-state UV-Vis spectra of: (a) HACA and HACA·$2H_2O$, and (b-d) cocrystal **1-3** and its pure components.

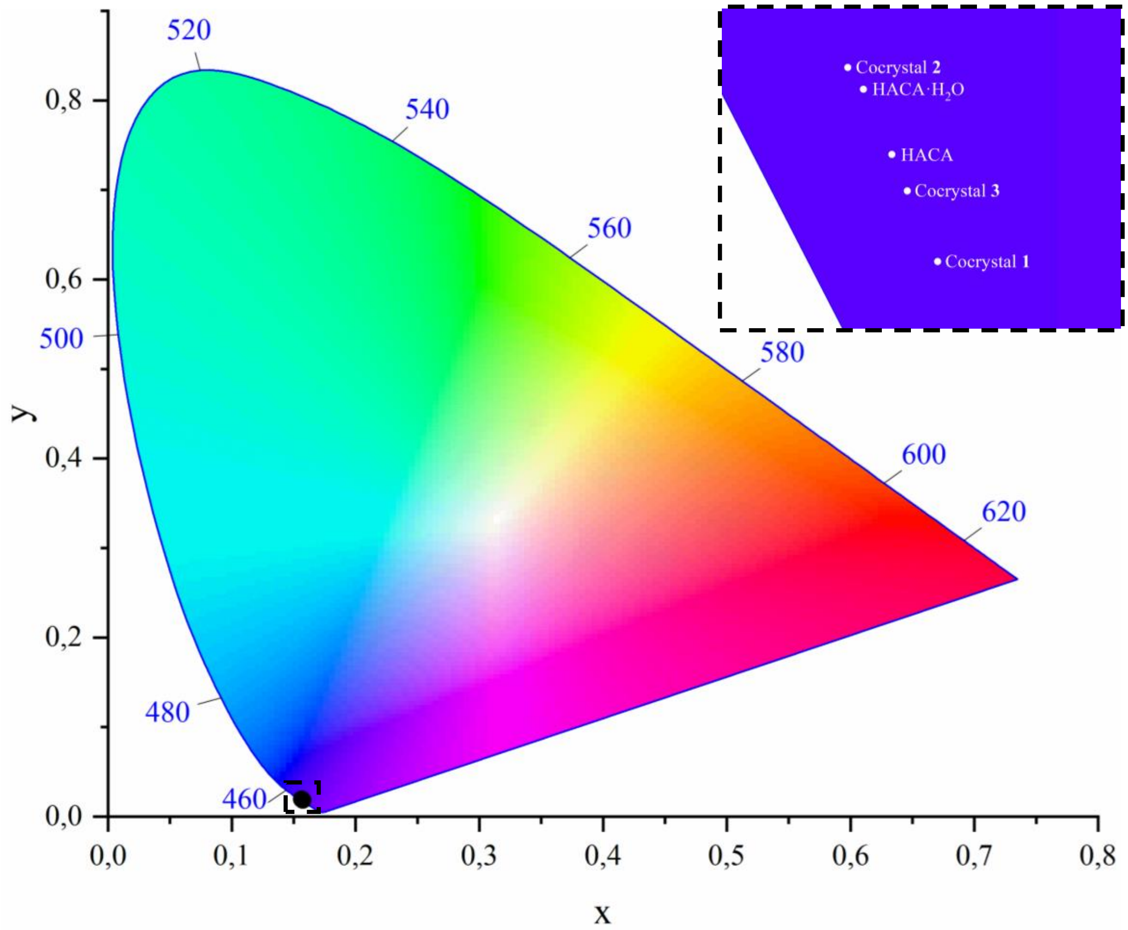

Figure S32. CIE 1931 chromaticity diagram of HACA, HACA·2H_2O, and cocrystals **1-3**. Color coordinates (x,y): HACA (0.1567, 0.0192), HACA·2H_2O (0.1564, 0.0196), cocrystal **1** (0.1573, 0.0186), cocrystal **2** (0.1562, 0.0197), and cocrystal **3** (0.1569, 0.0190).

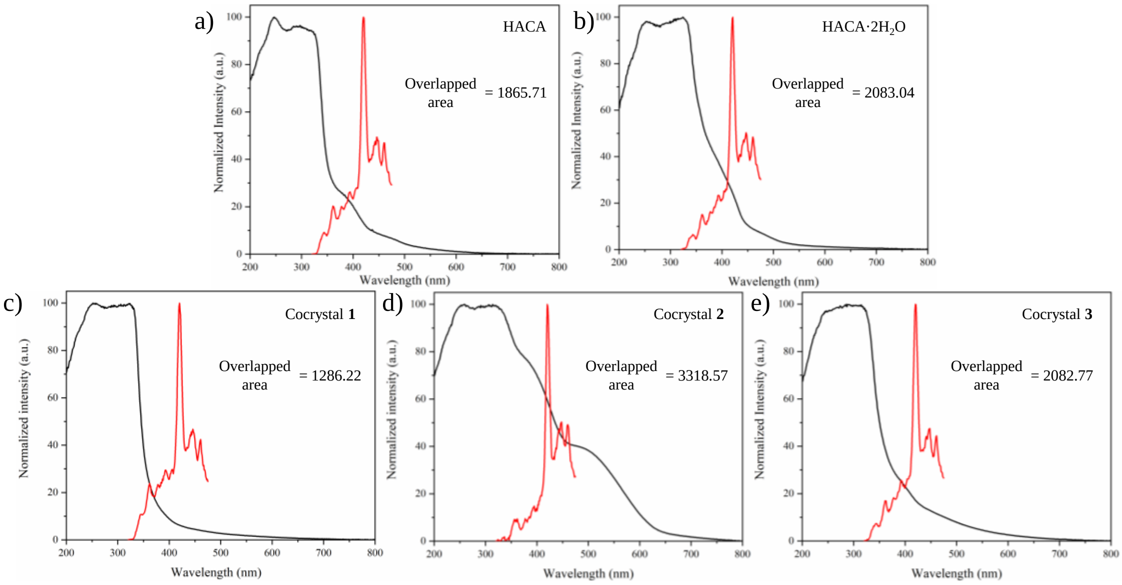

Figure S33. Normalized absorption and emission spectra of (a) HACA, (b) HACA·2H_2O, and cocrystals (c-e) **1-3** with their calculated overlapped areas.

References

(1) Ji, W.; Yuan, H.; Xue, B.; Guerin, S.; Li, H.; Zhang, L.; Liu, Y.; Shimon, L. J. W.; Si, M.; Cao, Y.; Wang, W.; Thompson, D.; Cai, K.; Yang, R.; Gazit, E. Co-Assembly Induced Solid-State Stacking Transformation in Amino Acid-Based Crystals with Enhanced Physical Properties. *Angew. Chem. Int. Ed.* **2022**, *61*, e202201234. https://doi.org/10.1002/anie.202201234.

(2) Ji, W.; Xue, B.; Bera, S.; Guerin, S.; Liu, Y.; Yuan, H.; Li, Q.; Yuan, C.; Shimon, L. J. W.; Ma, Q.; Kiely, E.; Tofail, S. A. M.; Si, M.; Yan, X.; Cao, Y.; Wang, W.; Yang, R.; Thompson, D.; Li, J.; Gazit, E. Tunable Mechanical and Optoelectronic Properties of Organic Cocrystals by Unexpected Stacking Transformation from H- to J- and X-Aggregation. *ACS Nano* **2020**, *14*, 10704–10715. https://doi.org/10.1021/acsnano.0c05367.

(3) Ji, W.; Xue, B.; Yin, Y.; Guerin, S.; Wang, Y.; Zhang, L.; Cheng, Y.; Shimon, L. J. W.; Chen, Y.; Thompson, D.; Yang, R.; Cao, Y.; Wang, W.; Cai, K.; Gazit, E. Modulating the Electromechanical Response of Bio-Inspired Amino Acid-Based Architectures through Supramolecular Co-Assembly. *J. Am. Chem. Soc.* **2022**, *144*, 18375–18386. https://doi.org/10.1021/jacs.2c06321.

(4) Liu, G.-F.; Zhu, L.-Y.; Ji, W.; Feng, C.-L.; Wei, Z.-X. Inversion of the Supramolecular Chirality of Nanofibrous Structures through Co-Assembly with Achiral Molecules. *Angew. Chem. Int. Ed.* **2016**, *55*, 2411–2415. https://doi.org/10.1002/anie.201510140.

(5) Cherukuvada, S.; Guru Row, T. N. Comprehending the Formation of Eutectics and Cocrystals in Terms of Design and Their Structural Interrelationships. *Cryst. Growth Des.* **2014**, *14*, 4187–4198. https://doi.org/10.1021/cg500790q.
